# Racial and economic disparities in coastal access and engagement mediate the ocean’s contribution to human wellbeing

**DOI:** 10.1038/s41467-026-75034-4

**Published:** 2026-07-13

**Authors:** Timothy H. Frawley, Jennifer C. Selgrath, Emma Kim Charlotte Gee, Taylor A. Triviño, Manuela Díaz, Jillian M. Lyles, Rachel Seary, Larry B. Crowder, Corey Garza, Elliott L. Hazen, Teresa Romero, Katherine L. Seto

**Affiliations:** 1 Institute of Marine Science, University of California Santa Cruz, Santa Cruz, CA USA; 2 California Marine Sanctuary Foundation, Santa Barbara, Monterey, CA USA; 3 Environmental Studies Department, University of California Santa Cruz, Santa Cruz, CA USA; 4 School of Aquatic and Fishery Sciences, University of Washington, Seattle, WA USA; 5 Bren School of Environmental Science and Management, University of California Santa Barbara, Santa Barbara, CA USA; 6 Emmett Interdisciplinary Program in Environment and Resources, Stanford University, Stanford, CA USA; 7 Hopkins Marine Station, Stanford Doerr School of Sustainability, Pacific Grove, CA USA; 8 Durrell Institute of Conservation and Ecology (DICE), School of Natural Sciences, University of Kent, Canterbury, UK; 9 Native Coast Action Network, Santa Ynez, Lompoc, CA USA

**Keywords:** Sustainability, Ocean sciences, Social sciences

## Abstract

Understanding what shapes ocean access and its contribution to human wellbeing is critical for equitable and sustainable marine resource management. Using a community-engaged research approach, we examine responses from 1691 surveys administered across central California (USA), an iconic coastline renowned for its biodiversity and cultural significance, to explore the factors mediating ocean use and benefits. We find that a) the ocean’s subjective and relational contributions to human wellbeing outweighed material contributions; and b) these benefits are constrained for vulnerable communities and underserved populations by personal (e.g., lack of interest or affordability), physical-environmental (e.g., lack of infrastructure or exposure to pollution), and knowledge (e.g., how to safely and legally engage) barriers. Our results highlight how structural exclusion from ocean spaces limits wellbeing for marginalized groups, a critical feedback loop that may perpetuate inequality and degrade public health. Though California considers itself a world leader in ocean management, our findings indicate that there is more work to be done and offer actionable insight for advancing more inclusive and equity-centered approaches to coastal and ocean governance.

## Abstract (web summary)

Community-engaged research in California shows that the ocean’s primary contributions to local wellbeing are in fostering connection, strengthening relationships and improving mental health, yet structural barriers limit these benefits for marginalized communities.

## Introduction

Oceans and coasts sustain the livelihoods, well-being, and cultural heritage of billions of people worldwide. Yet, access to these environments remains deeply unequal, shaped by historical policies and cultural processes driving economic disenfranchisement and social exclusion^1,2^. Geography, gender, class, and race (and the interactions among them) all function to constrain or enhance people’s relationship with the ocean^3^. Western governance and policy paradigms primarily frame the ocean as a biophysical resource^4,5^; yet Indigenous, Black, Brown, and (global) Southern intellectual traditions have long recognized the ocean as a source of kinship, connection, identity, cosmology, and spirituality^6–8^. Where Anglo-European management strategies conceptualize humans as separate from nature, whether prioritizing resource extraction for macroeconomic gain or exclusionary measures for conservation, they have often functioned to undermine local rights, relationships, and knowledge systems, and reinforce the historical legacy of colonialism^9,10^. Indeed, mounting evidence suggests that where socio-cultural dimensions are not considered alongside ecological and economic metrics, many policy approaches may serve only to reinforce inequality and the institutional and legislative barriers perpetuating cycles of exclusion and environmental decline^11–14^.

In recent years, marine social scientists and commons scholars have advanced and refined theoretical frameworks describing the fundamental links between equity (defined broadly as the fair and just conditions and treatment of people), governance, and ocean sustainability^2,3,15^. Despite increasing recognition of the importance of equity considerations in the durability, legitimacy, and efficacy of coastal management, their assessment and operationalization remain an enduring challenge^3^. Empirical, place-based investigations concerning the structural factors mediating who can access and benefit from ocean ecosystems (and why) remain scarce, particularly across highly-developed, urbanized, and/or multicultural coastlines characterized by complex and incompletely understood patterns of use and proximity.

Coastal California is uniquely suited to addressing this research gap. California’s coastal and marine zones are of fundamental importance to the environmental and socioeconomic sustainability of the state, with coastal counties home to approximately 68% of the state’s 38.9 million residents^16^. The region’s diverse demographics have been shaped over time by waves of migration, dispossession, and exclusion. During the Spanish, Mexican, and American colonial eras, Indigenous societies rooted in sophisticated and reciprocal marine and terrestrial stewardship ethics were disrupted and forcibly displaced^17,18^. The late 19th and early 20th centuries saw the arrival of Chinese, Japanese, Italian, and Portuguese communities responsible for the development of commercial fisheries^19,20^, followed by the Second Great Migration of the 1940s, where booming wartime shipyards drew thousands of African American families from the Jim Crow South (i.e., a system of legally codified racial segregation and disenfranchisement in the southern United States)^21,22^. Yet many of these populations were subsequently dispossessed or barred from coastal property ownership through treaty violations or discriminatory land-use policies and legislation^18,23–25^. Most recently, a shift in the focus of the coastal economy from maritime industries to high-end tourism, luxury real estate, and information technology has fueled rapid resettlement, establishing a coastal curtain where much of the shoreline is disproportionately White, wealthy, and older as compared to the state’s diverse inland population^1,26^.

In the past decade, warming oceans, rising sea levels, and increases in the frequency and severity of extreme weather events have had a pronounced impact on marine ecosystems, coastal infrastructure, and human communities across the state^16,27^. With new threats to biodiversity and ecosystem services emerging as climate change accelerates^28,29^, local and regional resource managers concerned with resilience and adaptation have affirmed that climate justice and frontline community impacts are an important consideration in planning for the future^30,31^. Yet there remains a paucity of information concerning the processes and mechanisms functioning to mediate regional ocean access and benefits. Despite the progressive mandate of the California Coastal Act (which secured legal protection for public access in 1976) and other recent legislation (i.e., the Marine Life Management and Marine Life Protection acts), the regional mosaic of ocean health, amenities, and engagement continues to be influenced by legacies of discriminatory policies, uneven regulatory enforcement, and ongoing coastal gentrification^1,32^. Without explicit attention to the racial and economic disparities shaping patterns of ocean use and benefits, and the historical and sociocultural context in which they are rooted, future interventions may only serve to reinforce the systemic inequalities driving uneven climate vulnerability and adaptive capacity^33,34^.

**Fig. 1:**
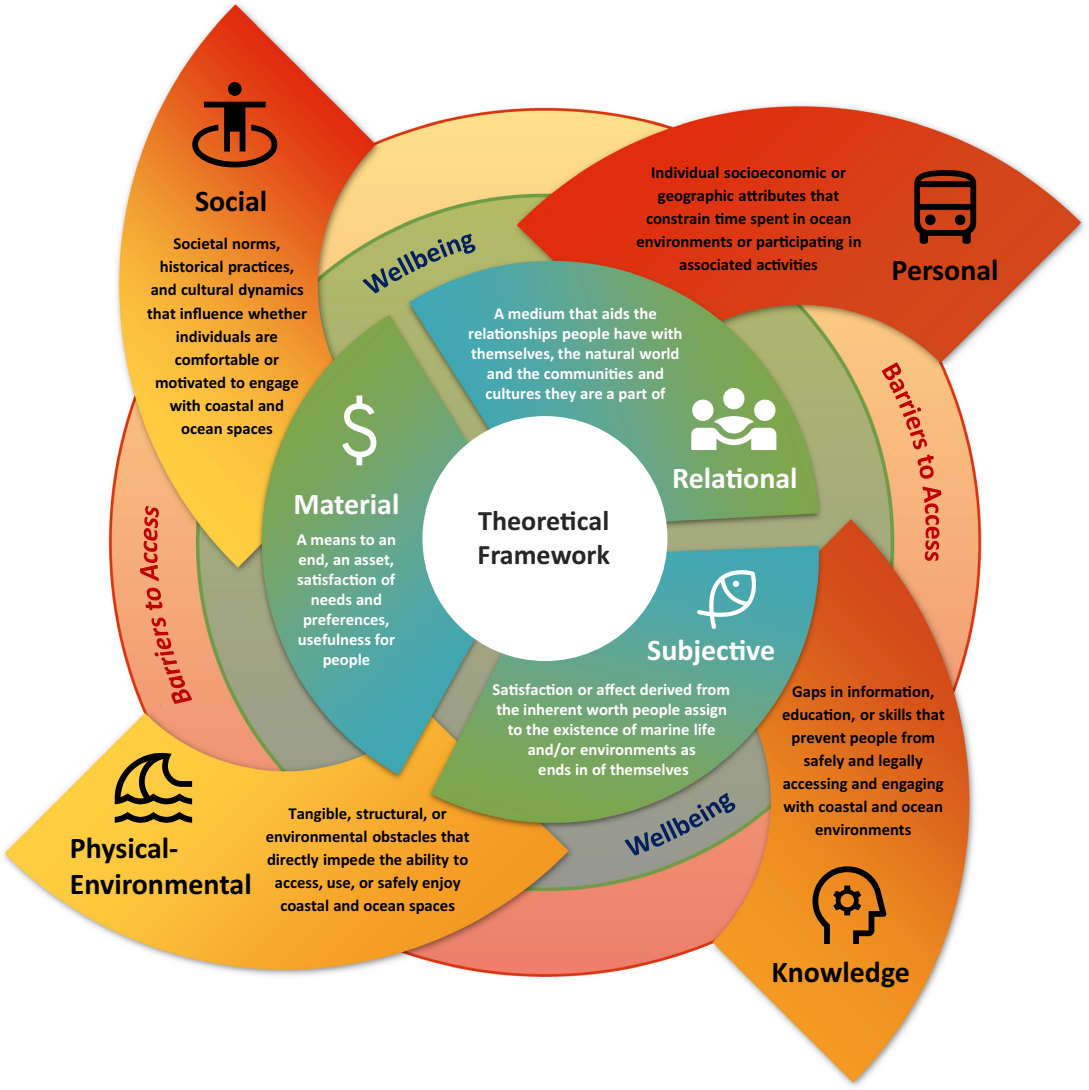
A summary of the synthetic, theoretical framework informing the study’s conceptualization of barriers to ocean access (see Wang et al., 2015; Byrne et al., 2009) and the ocean’s contributions to human wellbeing (see White et al., 2010; Weeratunge et al., 2014; Allison et al., 2023). Continuous color palettes are used to illustrate the interconnected, multidimensional nature of access barriers and well-being dimensions. A tabular version of this graphic, with exemplary sub-categories, is included as Supplementary Table 1 & 2. Icons from Google Material Icons (Apache License 2.0).

Marine conservation and governance scholarship has generally lagged behind its terrestrial counterparts in addressing issues of access, equity, and environmental justice^35–38^ and their examination through an intersectional lens of race, class, and gender^39,40^. Evolving from landmark research documenting the disproportionate toxic exposure of communities of color in the Southwest US^41^, environmental justice is a dynamic, overarching framework that advances a future where all people have the right to live in healthy and sustainable environments. This vision is realized through three interconnected components of equity: fair distribution of environmental benefits and burdens (distributional equity), meaningful participation in decision-making (procedural equity), and recognition of diverse social values, vulnerabilities, and capabilities (recognitional equity)^37,38,42^. Widespread acknowledgement of the value of such concepts in the ocean sciences is more recent, as scholarly debate concerning how they are best defined and assessed continues^3,14,15,43^. Building upon this evolving scholarship, our study examines two distinct but interrelated dimensions of the human-ocean relationship, which we argue are fundamental for understanding how ocean benefits are distributed across diverse coastal populations: the structural barriers that mediate access to ocean and coastal spaces and the ocean’s contributions to human wellbeing (Fig. 1).

Western governance models (i.e., the Public Trust Doctrine, the California Coastal Act) typically manage ocean and coastal spaces through a universalist lens, assuming that physical access to coastal spaces directly translates into societal benefits. Yet the right to enter an environment and the ability to derive benefits from it are not the same. Drawing on theories of access^44–46^, we suggest that the ability to benefit from ocean and coastal spaces is mediated by a bundle of powers influenced by physical-environmental, personal, social, and knowledge factors. Thus, we consider barriers to ocean access not as random inconveniences, but as structural processes (i.e., persistent institutional, economic, and social arrangements that shape individual agency) responsible for determining the distribution of ocean benefits across the California coast. Informed by previous literature^35,44–46^, we measure barriers to ocean access through nine Likert-scale survey prompts operationalized across four dimensions (see Supplementary Table 1): *personal* (e.g., transportation, proximity, affordability), *social* (e.g., welcomeness, belonging, social marginalization), *physical-environmental* (e.g., amenities, pollution, exposure to hazardous conditions), and *knowledge* (e.g., understanding of rules and regulations, possession of necessary skills, familiarity with access points).

Just as access cannot be reduced to the right to physical entry, considering the benefits provided by ocean and coastal spaces requires moving beyond the neoclassical economic indicators emphasized by many modern management frameworks. Global discourse surrounding the Blue Economy increasingly frames the ocean as a frontier for development, emphasizing material outputs, such as food security, energy, and employment^4,5^. Yet, as human relationships with the ocean are varied and complex, marine social scientists and development scholars have advocated for a broader conceptualization of wellbeing that embraces the holistic world-view of alternative epistemologies^7,8^ and considers subjective and relational benefits alongside material ones^47–49^. Drawing on theories of human wellbeing^47–49^, we measure the ocean’s perceived contribution to wellbeing through nine Likert-scale survey prompts operationalized across three dimensions (see Supplementary Table 2): *material* (e.g., food, income, and physical health), *relational* (e.g., community, cultural identity, intra-household relations), and *subjective* (e.g., mental health, spirituality, aesthetic experience). By coupling this multidimensional view of human wellbeing with a structural analysis of ocean access, this study aims to map how the ocean’s benefits vary across California’s diverse coastal populations.

This research was conducted by a multi-disciplinary collective of academic researchers and community practitioners who acknowledge that their perspectives are shaped by diverse backgrounds in marine social science, ecology, anthropology, Native American and Indigenous Studies, and sustainability science. The team, motivated to conduct place-based investigations concerning equity in ocean access and benefits, initially coalesced around a research call (i.e., public funds administered through the California Drought, Water, Parks, Climate, Coastal Protection, and Outdoor Access for All Act of 2018) with resources specifically allocated to address the needs of underserved communities. Here, we directly respond to the need to develop and test social indicators capable of monitoring and evaluating ocean equity^3,15,50^. Leveraging previous scholarship advanced by development studies, human geography, and marine social science^35,44,47,48^, we investigate the complex, multidimensional relationships between ocean access and benefits across California’s central coast, and identify variation in relevant patterns and processes across different demographic groups. Relying on research methods designed explicitly to engage communities designated as disadvantaged or severely disadvantaged by the California Environmental Protection Agency’s CalEnviroScreen index^51^, we use survey responses from 1691 individuals to address the following research questions: 1) How do socioeconomic, demographic, and geographic factors interact to shape ocean access? 2) What barriers pose the biggest obstacles in limiting different populations’ access to and engagement with ocean and coastal spaces? 3) How do the ocean’s contributions to human wellbeing vary across our study population, and how do different types of barriers amplify or diminish these contributions? By systematically identifying barriers to ocean access and evaluating their impacts on perceived contribution to human wellbeing, we hope to uncover the structural processes sustaining regional ocean inequality and identify specific leverage points for policy intervention.

## Results

**Fig. 2:**
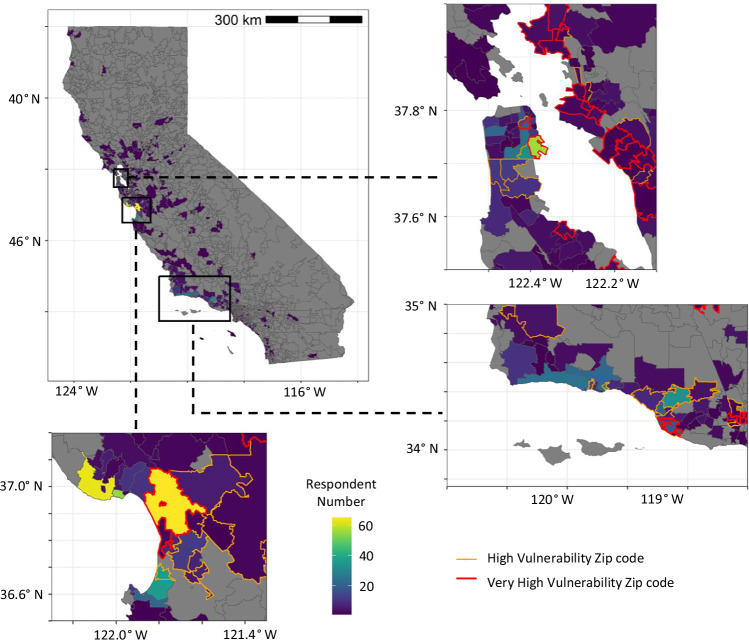
Map illustrating the distribution (by self-reported zip code of residence) of 1,691 survey responses used for analysis across California at-large, with insets highlighting the three focal regions where administration efforts were concentrated: Southeast San Francisco (most northern); the Greater Monterey Bay; and Santa Barbara, Oxnard, & Ventura (most southern). The zip codes assessed as having high and very high vulnerability (as based on CalEnviroScreen scores; see Methods) are outlined in thin orange (high vulnerability) and bold red (very high vulnerability) lines.

Racial and ethnic demographics of our survey sample were broadly representative of those across California. 40.3% of survey respondents identified as White (as compared to 34.7% of California residents providing information to the 2020 US Census), 29.1% identified as Hispanic or Latino (as compared to 39.4%), 20.3% Asian (as compared to 15.1%), 7.75% Black or African American (as compared to 5.4%), 3.7% American Indian or Alaskan Native (as compared to 0.4%), and 3.1% as Native Hawaiian or Pacific Islander (as compared to 0.4%) (Supplementary Fig. 1a). The survey sample was skewed towards younger individuals (51.1% <40 years old) from medium ($60,000–$119,999 annual earnings; 27%) and low (less than $59,999; 30.1%) income households (Supplementary Fig. 1b) that were frequent ocean users. The majority of respondents reported that they used ocean and coastal areas several times per month or several times per week (31.9% and 31.4%, respectively) (Supplementary Fig. 1c). When comparing the format of survey responses (in-person vs online administration) a comparatively larger proportion of White and Asian individuals from upper- and middle-income households provided information online (Supplementary Fig. 1a). Of the eight California zip codes most frequently reported as survey respondents’ primary residence; three were assessed as having high community vulnerability (Marina, *n* = 36; Santa Paula, *n* = 33; Visitacion Valley/Sunnydale, *n* = 33) and two were assessed as having very high community vulnerability (Watsonville, *n* = 65; Bayview-Hunter’s Point, *n* = 62) (Fig. 2).

### Demographic factors associated with ocean engagement

**Fig. 3:**
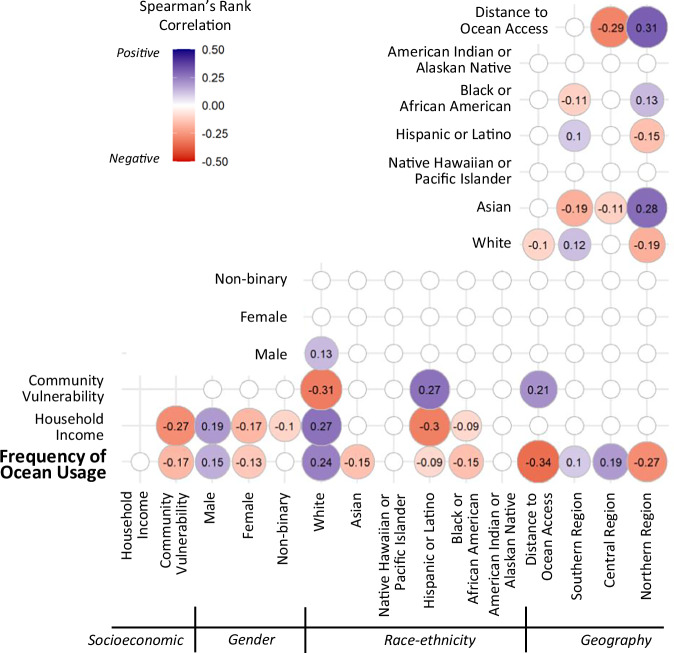
Correlation matrix displaying Spearman’s rank correlation values used to test for correlation between socioeconomic and geographic variables, gender, race-ethnicity, and frequency of ocean usage. Circles are sized and colored according to the magnitude and direction of the correlation; larger circles indicate stronger associations with purple circles representing positive correlations and red circles representing negative correlations. All correlation tests were two-sided. Only correlations significant at *p* < 0.05 following a Bonferroni correction to control for the family-wise error rate are displayed, with correlations within racial-ethnic, gender, and geographical categories (where variables contained redundant information, i.e., Male: Female) omitted to reduce the total number of comparisons. Geographic variables (distance to ocean access, region) and Community Vulnerability (as inferred by CalEnviroScreen score) were derived from the self-reported zip code of primary residence, while all other information was obtained using the survey instrument. Household income, frequency of ocean usage, community vulnerability, and distance to ocean access variables were each transformed into a 5-point, ordinal scale before analysis (see Methods).

A Spearman’s Rank correlation matrix, used to examine the relationship between demographic and behavioral variables assessed by the survey instrument, revealed a number of significant associations within and among socioeconomic, demographic, and geographic variables (Fig. 3). Examining the strongest, significant associations (*r* ≥ 0.200 & *p* < 0.05), survey respondents from the Northern region of our study area had a longer distance to travel to access ocean and coastal areas (*r* = 0.311), particularly as compared to the travel distance of Central region respondents (*r* = −0.291). Respondents in the Northern region also more frequently identified as Asian (*r* = 0.276). Consistent with coastal gentrification, individuals having to travel longer distances to access the ocean were likely to reside in more vulnerable communities (*r* = 0.213), as were individuals who self-described as Hispanic or Latino (*r* = 0.273). Identifying as Hispanic or Latino was additionally negatively correlated with household income (*r* = −0.297), while identifying as White was negatively correlated with community vulnerability (*r* = −0.312) and positively correlated with household income (*r* = 0.273). Overall, community vulnerability and household income were negatively correlated (*r* = −0.265). Looking at the relationships with reported frequency of ocean usage, the strongest negative correlations were with distance to the ocean (*r* = −0.344) and the Northern region (*r* = −0.268), with a somewhat weaker correlation (*r* = −0.175) with community vulnerability. The strongest positive correlation with frequency of ocean usage was with those survey respondents identifying as White (*r* = 0.239).

In addition to informing frequency of ocean usage, socioeconomic and demographic variables had a significant relationship with the type of activity that survey respondents valued most (Supplementary Fig. 2). Adventure water sports like surfing, snorkeling/scuba diving, and/or swimming or body surfing were comparatively more popular among individuals identifying as White with elevated household incomes that resided in communities with low vulnerability scores. In contrast, group or family gatherings, beach games or sports, and contemplative activities (meditation or appreciating views/sunsets) were comparatively more popular for low-income households and individuals from communities with high vulnerability scores. A strong, statistically significant divide was evident in those choosing fishing or collecting food as their most valued activity (the most popular activity overall for survey respondents; Supplementary Fig. 3): 35 and 36% of individuals from the two upper household income categories chose fishing as most valued (alongside 31% of individuals identifying as White) while only 13% of individuals from the lowest household income category chose fishing (alongside 11% and 14% of those individuals identifying as Black/African American or Hispanic/Latino). Looking at how activity profiles clustered across categories, the two lowest household income categories (annual earnings of $59,999 or less, and $60,000–$119,999) were grouped independently of the upper income households (Supplementary Fig. 2a). A parallel division existed in separating individuals according to community status, with individuals residing in communities of moderate-very high vulnerability clustered independent of those residing in communities with low or very low vulnerability scores (Supplementary Fig. 2c). With respect to racial-ethnic identities, those self-describing as Black or African American, Hispanic or Latino, and/or Asian (all of which were negatively, though weakly, correlated with frequency of ocean usage, see Fig. 3) were grouped independently of those identifying as Native Hawaiian or Pacific Islander, White, or American Indian or Alaskan Native (Supplementary Fig. 2b).

### Structural barriers to ocean access

**Fig. 4:**
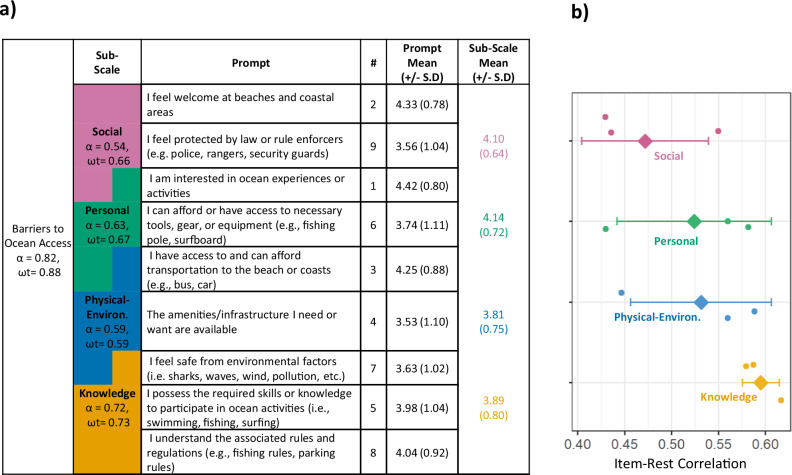
Consistency and quality of survey prompts used to measure the composite scale of barriers to ocean access and its social (pink), personal (green), physical-environmental (blue), and knowledge (yellow) dimensions. All prompts were measured using a 5-point Likert scale that was subsequently transformed into numerical values (i.e., strongly disagree = 1, strongly agree = 5). **a** presents the i) Cronbach’s alpha (α) and McDonald’s omega (ωt) metrics that were used to measure the internal consistency of the entire scale (and each dimensional sub-scale; ii) the individual survey prompts contributing the composite scale (*n* = 9) and each sub-scale (*n* = 4; with color coding meant to indicate where prompts were considered in multiple sub-scales) and iii) the mean scale value (i.e., score) calculated for each prompt and sub-scale (*n* = 1600). Given the framing of the prompts, smaller mean values are associated with greater reported barriers. **b** summarizes the Item-Rest correlation values for the prompts comprising each sub-scale (*n* = 3 prompts per sub-scale), illustrating both the values of individual prompts (points) and sub-scale means ± S.D (diamonds). Larger Item-Rest correlation values indicate dimensions that were most effective at measuring the composite scale’s core construct. Individual Item-Total & Item-Rest correlation values measuring the quality of each prompt in measuring the composite barrier scale and sub-scales can be found in Supplementary Table 4.

In considering a composite scale designed to measure barriers to ocean access and dimensional sub-scales composed of personal, social, physical-environmental, and knowledge factors, physical-environmental factors were assessed as representing the largest barriers to ocean access, while personal factors were the smallest (i.e., sub-scale means in Fig. 4a, where smaller means represent larger barriers). The results of a repeated measures ANOVA indicated significant differences between sub-scale scores (*p* < 0.001), though a post-hoc Tukey Honestly Significant Difference (HSD) test revealed no significant differences between social and personal factors. However, mean item-rest correlation values (i.e., the correlation between an item and the rest of the scale excluding that item, used here to evaluate which items were most effective in measuring the composite scale’s core construct, see Methods) suggest that knowledge factors (0.59 ± 0.02), accounted for the greatest differences in the overall barriers score (Fig. 4b), with the knowledge sub-scale being the most reliable and consistent sub-scale according to reliability metrics (α = 0.72, ωt = 0.73). Indeed, across all nine prompts, the first and second highest item-rest correlation values (i.e., understanding rules and regulations (0.61) & feeling safe from environmental factors (0.59); see Supplementary Table 4) were associated with knowledge dimension prompts.

**Fig. 5:**
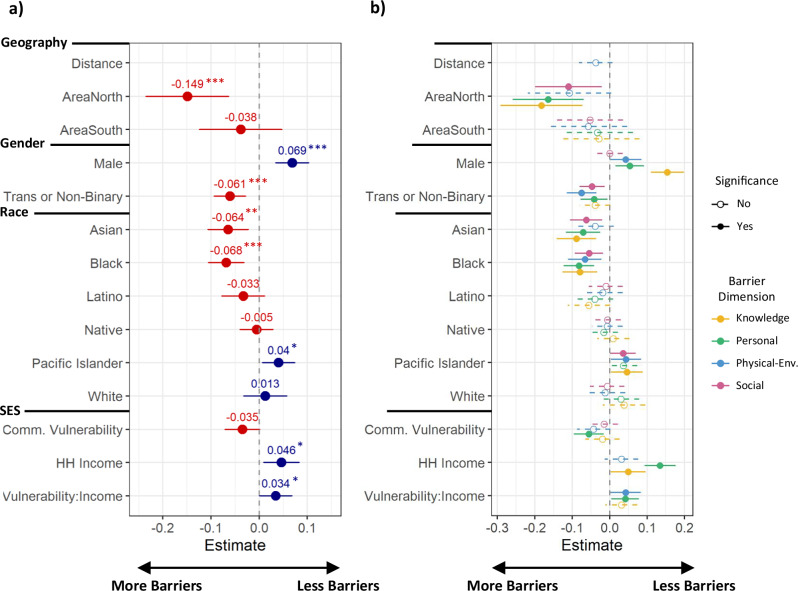
Perceived barriers to ocean and coastal zone access as a function of geographic, gender, race, and socioeconomic (SES) variables as quantified using ordinary least squares regression. In both panels, points represent the standardized regression coefficient estimate for each predictor (the measure of center), and error bars represent the 95% confidence interval around the estimate. Statistical significance for each coefficient was assessed using two-sided *t*-tests with no adjustment made for multiple comparisons (see Methods). Coefficient estimates, standard errors, and exact p-values for all model coefficients are provided in the complete regression tables linked in the Data Availability section. Results are shown for a barrier scale composed of all nine prompts (**a**) as well as sub-models where knowledge (yellow), personal (green), physical-environmental (blue), and social (pink) dimensional scales (**b**) were derived from a subset of those prompts (see Fig. 4a). An alternate version of this figure, in which Female is included in the model rather than Male, can be found in the supplement (Supplementary Fig. 4). Points in panel A are colored according to coefficient sign (red = negative estimate, blue = positive estimate) and labeled according to significance (* *p* < 0.05, ** *p* < 0.01, *** *p* < 0.001). Points in panel B are colored according to the dimensional scale to which they correspond with solid lines and points corresponding to significant coefficients (*p* < 0.05). The sample for these regression analyses (*n* = 1255) was limited to survey respondents providing complete answers to all relevant survey questions. Note that not all models include the same predictors (e.g., Distance was not included in the composite model illustrated in (**a**) as the structure of each was determined by forward, stepwise model selection using AIC criteria (see Methods*)*. A redundancy analysis, which explores the degree to which geographic, demographic, and socioeconomic variables explain variation in reported barriers (as specified at the level of individual prompts), is shown in Supplementary Fig. 6 & 6.

Ordinary Least Squares (OLS) regression was used to examine the geographic, gender, racial, and socioeconomic predictors of barriers to ocean access (i.e., the composite scale). When tested as individual predictors) Residence in the Northern region and identifying as Asian or Black/African American, were each significantly associated with reporting more barriers (Fig. 5a). The most significant and constituent contributors to these trends were knowledge and personal factors (Fig. 5b), with knowledge factors also contributing to substantial (though non-significant) barriers for Latino respondents. Conversely, self-describing as male, Native Hawaiian/Pacific Islander, or reporting elevated household income were each significantly associated with reporting fewer barriers to ocean access (Fig. 5a). Knowledge factors contributed most strongly to this result for male respondents while personal factors contributed most strongly for individuals with elevated household income (Fig. 5b). While community vulnerability was not a significant predictor on its own, the interaction with household income was significant (Fig. 5a, *p* < 0.05), indicating that low income households in vulnerable areas may face unique and/or more pronounced personal and physical-environmental barriers (Fig. 5b) when accessing ocean and coastal spaces. In looking at variability in model construction (i.e., predictors), i) distance to ocean access only improved model fit when evaluating physical-environmental barriers; and ii) socioeconomic factors (i.e., household income, community vulnerability) did not improve model fit when evaluating social barriers.

### Distribution of wellbeing benefits

**Fig. 6:**
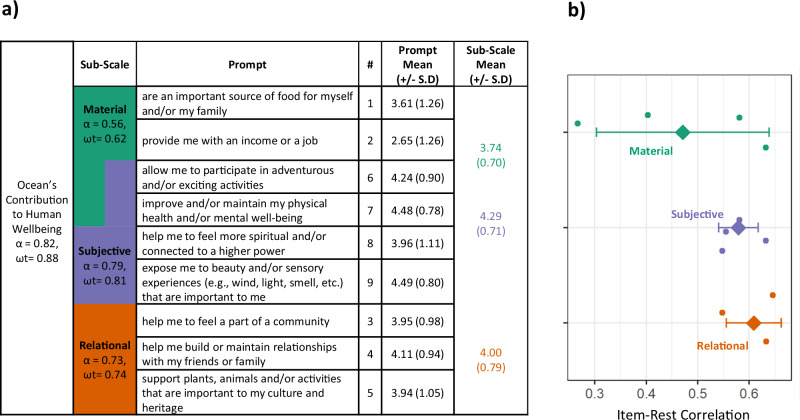
Consistency and quality of survey prompts used to measure the composite scale of perceived contribution of ocean and coastal spaces to human wellbeing and its material (teal), subjective (purple), and relational (orange) dimensions. All prompts were measured using a 5-point Likert scale that was subsequently transformed into numerical values (i.e., strongly disagree = 1, strongly agree = 5). **a** presents the i) Cronbach’s alpha (α) and McDonald’s omega (ωt) metrics that were used to measure the internal consistency of the entire scale and each dimensional sub-scale; ii) the individual survey prompts contributing the composite scale (*n* = 9) and each sub-scale (*n* = 3 or 4) with color coding meant to indicate where prompts were considered in multiple sub-scales; and iii) the mean scale value (i.e., score) calculated for each prompt and sub-scale (n = 1568). Given the framing of the prompts, larger mean values are associated with greater perceived contribution to human wellbeing. **b** summarizes the Item-Rest correlation values for the prompts comprising each sub-scale (*n* = 3 or 4 prompts per sub-scale), illustrating both the values of individual prompts (points) and sub-scale means ± S.D (diamonds). Larger Item-Rest correlation values indicate prompts and/or sub-scales that were most effective at measuring the composite scale’s core construct. Individual Item-Rest and Item Total correlation values measuring the quality of individual prompts in measuring the composite wellbeing scale and sub-scales can be found in Supplementary Table 5.

**Fig. 7:**
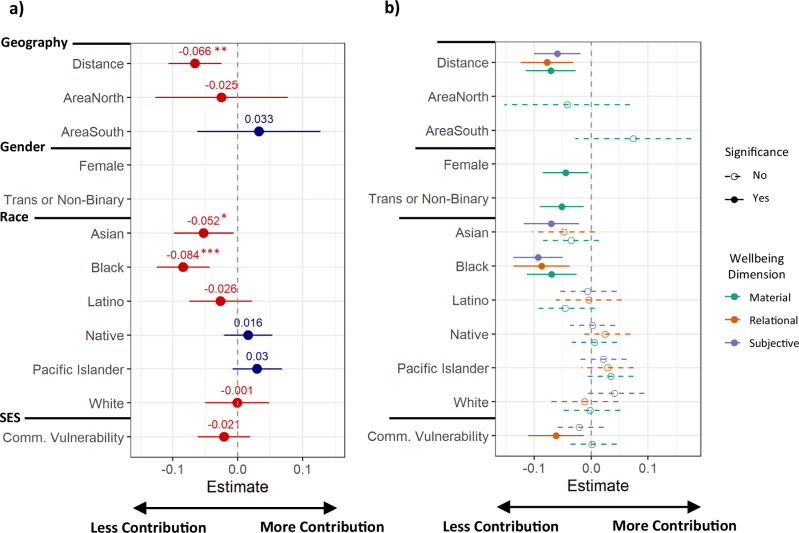
Perceived contribution of ocean and coastal zones to human wellbeing as a function of geographic, gender, race, and socioeconomic (SES) variables as quantified by ordinary least squares regression. In both panels, points represent the standardized regression coefficient estimate for each predictor (the measure of center), and error bars represent the 95% confidence interval around the estimate. Statistical significance for each coefficient was assessed using two-sided *t*-tests with no adjustment made for multiple comparisons (see *Methods*). Coefficient estimates, standard errors, and exact p-values for all model coefficients are provided in the complete regression tables linked in the Data Availability section. Results are shown for a wellbeing scale composed of all nine prompts (**a**) as well as sub-models where material (teal), relational (orange), subjective (purple) dimensional scales (**b**) were derived from a subset of those prompts (see Fig. 6a). An alternate version of this figure, in which Male is included in the model rather than Female, can be found in the supplement (Supplementary Fig. 5). Points in (**a**) are colored according to coefficient sign (red = negative estimate, blue = positive estimate) and labeled according to significance (* *p* < 0.05, ** *p* < 0.01, *** *p* < 0.001). Points in panel B are colored according to the dimensional scale to which they correspond with solid lines and points corresponding to significant coefficients (*p* < 0.05). The sample for these regression analyses (*n* = 1237) was limited to survey respondents providing complete answers to all relevant survey questions. Note that not all models include the same predictors (e.g., Female was not included in the composite model illustrated in (**A**) as the structure of each was determined by forward, stepwise model selection using AIC criteria (see Methods).

We designed a parallel analysis to measure the contribution of ocean and coastal spaces to human wellbeing (Figs. 6, 7). We found that material wellbeing factors (e.g., income, employment, food security, etc.), often prioritized by management frameworks reliant upon neoclassical economic indicators and quantitative output targets, scored lower than subjective and relational wellbeing factors (i.e., sub-scale means in Fig. 6a, where larger values represent greater perceived contribution to human wellbeing). A repeated measures ANOVA and post-hoc Tukey HSD tests revealed significant differences between all sub-scales (Figure 6a). However, the material sub-scale was less reliable (α = 0.56, ωt = 0.62) than the subjective (α = 0.79, ωt = 0.81) and relational (α = 0.73, ωt = 0.74) sub-scales primarily responsible for determining the overall difference in wellbeing scores (relational mean item-rest correlation = 0.61 ± 0.05; subjective mean item-rest correlation = 0.58 ± 0.04; Fig. 6b). Across all nine prompts, two of the three prompts with the highest item-rest correlation values (i.e., help me to feel part of a community (0.646), help me build relationships with friends and family (0.632)) were associated with the relational dimension (Supplementary Table 5).

OLS regression used to examine the geographic, gender, race, and socioeconomic predictors of the ocean’s perceived contribution to human wellbeing found that larger geographic distances to the ocean and respondents self-describing as Asian or Black/African American were associated with significantly less ocean wellbeing (Fig. 7). Neither income nor gender variables improved model fit when considering the total wellbeing scale. In considering variation across dimensional sub-scales, self-describing as Black/African American was associated with significantly lower contributions to wellbeing across all three dimensions (i.e., material, subjective, and relational) while only subjective wellbeing was significantly lower for respondents self-describing as Asian. Community vulnerability (conceptualized here as the product of environmental exposure to pollution and socioeconomic sensitivity; see Methods), the only socioeconomic variable included in the aggregate model, was significantly associated only with lower relational well-being. In terms of variation in model construction, gender variables were useful in improving model fit for the material sub-scale, as individuals self-describing as male reported significantly more material benefits.

### Processes and pathways shaping the distribution of ocean benefits

**Fig. 8:**
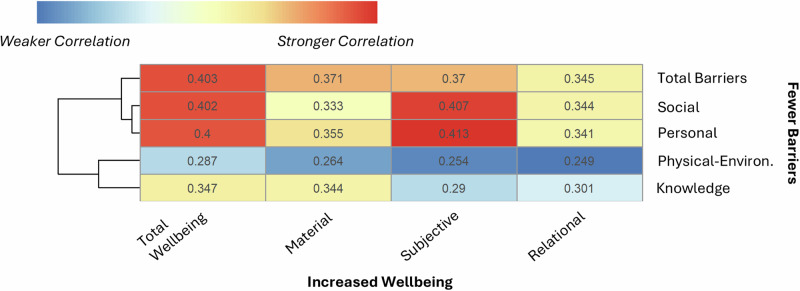
Heatmap comparing the strength of association between the Barriers scale (and its dimensional sub-scales) and the Wellbeing scale (and its dimensional subscales). Numerical labels represent the Spearman rank correlation coefficient values that were the basis for comparison with red values representing stronger correlations and blue values representing weaker correlations. All tests were two-sided, and all correlations remained highly significant (*p* < 0.01) following the application of a Bonferroni adjustment to correct for the family-wise error rate.

The ocean barrier index and the ocean wellbeing index were significantly correlated (*r* = 0.403, *p* < 0.0001), meaning that individuals who reported more barriers to ocean access reported diminished contributions of the ocean to their wellbeing (Fig. 8). Social (*r* = 0.401, *p* < 0.0001) and personal (*r* = 0.400, *p* < 0.0001) barriers played a comparatively stronger role in defining this association compared to physical-environmental barriers (*r* = 0.287, *p* < 0.0001) and knowledge barriers (*r* = 0.347, <0.0001), with subscale correlations the highest between diminished subjective wellbeing and social (*r* = 0.407, <0.0001) and personal (*r* = 0.413, <0.0001) barriers. Disaggregated analysis (Supplementary Fig. 8) used to examine the relationship between individual prompts suggests this relationship was largely driven by variable interest in ocean activities (a prompt considered in both social and personal categories) across survey respondents. The three highest correlations in examining pairwise prompt associations were found between the barrier prompt designed to assess ocean interest and the three subjective well-being prompts. An additional relationship worth noting was the strong correspondence between lack of required skills and abilities (a knowledge barrier) and diminished subjective and relational benefits. Overall, differences in reported interest, skills & knowledge across respondents were most strongly associated with income and community status, as well as race (with Black or African American, Asian, and Latino respondents driving the most variation; see Supplementary Fig. 6, Supplementary Fig. 7).

## Discussion

Our findings reveal how racial identity, socioeconomic status, gender, and geography interact to shape ocean access and wellbeing in ways that reflect historical legacies of discrimination and dispossession rather than neutral differences in preference. Though the California Coastal Act establishes the principle of universal public coastal access, legal rights and physical access alone have failed to translate into meaningful outcomes for underserved and historically marginalized communities. Interpreted through the lens of intersectional environmental studies^39,40^, significant barriers reported by Black and Asian American respondents, particularly knowledge (e.g., understanding rules and possessing necessary skills) and personal (e.g., lack of interest or affordability) barriers, reflect structural processes embedded in racialized histories. Existing literature cautions that what may appear as disinterest among such communities often functions as protective disengagement from hostile spaces where they have experienced discrimination or marginalization^52–54^. Following 19th-century fragmentation and removal of tribal communities^18^, the first half of the 20th century saw the continuation of state-sponsored policy through which people of color were systematically excluded from coastal spaces via destruction of Asian American fishing villages^20^, the forced displacement of Black coastal communities, and the segregation of public beach areas^25,55^. More recently, restrictive housing covenants and lending practices^56^, uneven investment in community infrastructure and environmental education^57^, and accelerating coastal gentrification^1,58^ continue to shape who can access and benefit from ocean areas across the state. Even where historically discriminated and/or underserved communities persist along the coast, legacies of pollution and environmental injustice (particularly across urban and agricultural shorelines) limit direct engagement with and benefits from the ocean^22^.

As demonstrated by the significant interaction between household income and community vulnerability in our analysis of barriers to ocean access, our results demonstrate that an intersectional lens is critical to understanding the compounding effects of exclusion and discrimination for those who hold multiple marginalized identities. This is particularly evident with respect to gender; although the gendered dimensions of ocean access and wellbeing have received less research attention compared to terrestrial conservation and agricultural sectors^59,60^, our results support the assertion that gender is a critical determinant of how people differentially access and benefit from ocean ecosystems^61,62^. Gender emerged from our analysis as one of the strongest predictors of barriers to ocean access, with females reporting significantly more barriers and fewer material benefits than male respondents. Structural processes associated with political relations of ownership and control, differentiated social vulnerability, and gendered household roles^62,63^ all likely interact in shaping the varying levels of ocean access and activity driving this result. Note that while strong associations were also observed among transgender and non-binary respondents, we hesitate to draw broad inferences due to the limited (n = 42) and geographically constrained sample of this demographic.

While California’s coastal management often prioritizes extractive uses and material outcomes (i.e., employment, food security, and economic growth)^5,64^, our findings suggest that relational and subjective benefits are far greater for many individuals, particularly those from low-income households and/or vulnerable communities. Across our study population, individuals associated with such demographics expressed a consistent preference for engaging in social and/or contemplative activities (i.e., group and family gatherings, informal beach sports, reflection and meditation, etc.) when interacting with oceans and coasts. While recreational fishing emerged as one of the most important ocean uses in our survey, previous literature suggests that this activity is often motivated by a desire to spend time in and connect with nature rather than purely subsistence goals^65,66^. Our findings suggest that the narrow focus of blue economy initiatives (and the Western management frameworks in which they are embedded) overlooks important wellness benefits and constrains the ocean’s benefits to society. This assertion is supported by an expanding body of public health^67,68^, marine social science^10,48,69^, and ocean governance^2,43^ literature, which suggests that broader and more inclusive management strategies are required to elevate the diverse forms of ocean engagement that support relational and subjective wellbeing.

The therapeutic effects of marine environments, often referred to as the blue mind phenomenon, have been linked to reduced stress, improved mood, enhanced social relationships, and even clinical improvements in symptoms of depression and severe mental illness^70–72^. Activities, such as open water swimming, beach walking, and surfing, have shown promise not only as recreational practices supporting essential physical activity but also as low-cost, community-embedded mental health interventions^73–75^. Yet, as we demonstrate, such benefits are not broadly understood nor equally distributed. Despite valuing the ocean as an opportunity for connection, expression, contemplation, and healing, the subjective and relational benefits may remain unrealized for vulnerable communities and underserved demographics due to the existence of personal, physical-environmental, and knowledge barriers. By impeding ocean access to those individuals who might stand to benefit the most (in body and mind) from subjective and relational benefits, this disconnect may reinforce public health disparities at a substantial ethical and economic cost^68^. Indeed, mental ill-health is projected to account for more than half of the global economic burden attributable to non-communicable diseases by 2030^76^. By drawing upon environmental justice frameworks that prioritize recognition and relational values^11,42,77^, coastal management approaches designed to mitigate climate impacts or redress environmental degradation should move beyond efforts to provide physical infrastructure and/or economic opportunity to explicitly consider the social, cultural, and emotional pathways through which the ocean supports human wellbeing.

It is important that our findings be interpreted in light of several limitations. Despite our intentional sampling efforts to reach disadvantaged and underrepresented communities in coastal counties across central California, the composition of our final sample resulted in the overrepresentation of some demographics (i.e., Asian & Native Hawaiian or Pacific Islander) and the underrepresentation of others (i.e., Hispanics and Latinos) as compared to California at large. Likewise, though we attempted to mitigate such dynamics by disseminating the survey online and sampling inland community hubs (i.e., laundromats, community events, markets, etc.) in addition to coastal access points, field work focused on disadvantaged zip codes within coastal counties likely failed to capture many of the dynamics most relevant to California’s diverse (and by many metrics, most vulnerable) inland population. In addition, though we believe our research design was robust (as grounded both in relevant literature and the lived experience of the communities that provided data; see Methods) we acknowledge that a) our assessment of many nuanced and multidimensional constructs was ultimately constrained by the number, detail and presentation of the underlying prompts; and b) a degree of simplification was required to accommodate the breadth of the complete survey instrument. Finally, though our analysis of cross-sectional survey data serves as a valuable baseline description of current conditions, additional research is required to definitively determine and evaluate causal pathways. Future research designed to address such aims would be well-served in considering longitudinal study design and/or experimental interventions, objective (rather than self-reported) measurements, and increased integration with qualitative inquiry^78^.

California considers itself a world leader in protecting and managing ocean environments for public benefit, yet our results add to a growing body of literature that suggests that there is more work to be done to achieve the equitable distribution of ocean benefits^26^. Despite the progressive mandate of policy to enhance opportunities for recreation, education, and wellbeing through equitable ocean access across the state (e.g., bills, such as the California Coastal Act, the Marine Life Protection Act, the National Marine Sanctuary Act, and Expanding Access to California’s Local City and County Parks & Beaches), such benefits remain disproportionately available to affluent, white communities located in close proximity to the coast. Other demographics–particularly Black and Asian Americans, low-income households, environmentally vulnerable communities, and/or women and non-binary people–continue to face persistent and compounding barriers limiting ocean access and use.

To advance ocean equity, governments, funders, and practitioners must invest in creating, restoring, and/or sustaining meaningful connections to ocean spaces (Table 1). More specifically, our finding that knowledge represents one of the most consistent and influential barriers to ocean access suggests that targeted (and subsidized) Education and Outreach efforts should be prioritized. Compared to green space, the impact of knowledge on ocean access may be comparatively more pronounced as many forms of engagement and participation require ocean literacy and water competence, a specific set of physical skills and safety knowledge often informally transmitted and unequally distributed across society^10,52^. While diverse forms of local ecological knowledge helped support resilient human-ocean relationships in California’s not-too-distant past^17,20^, knowledge systems are subject to degradation when exposed to rapid and/or compounding processes of social and environmental change^8,79^. Efforts to build ocean literacy across underserved communities should be cognizant of such dynamics and seek to promote diverse, co-created and user accessible knowledge systems rather than reinforcing existing power dynamics by exclusively relying upon Western language and science^69^. Likewise, recruiting enforcement and management staff that reflect community demographics and providing cultural competency training through Workforce Development initiatives may help ensure that rules, regulations, and safety information are communicated effectively and inclusively.

**Table 1:** Policy recommendations to reduce barriers to ocean access and promote equitable distribution of ocean benefits

Intervention Target	Examples	Expected Outcome
Education & Outreach	• Partner with established local NGOs to host inclusive, community-focused coastal events • Provide priority access (and/or reduced fee) days for underserved communities • Offer free or low-cost swimming lessons and/or equipment rental programs • Launch public outreach campaigns to raise awareness of local ocean areas, wildlife, and recreation opportunities • Create multilingual resources and signage that clearly describe local rules, safety information, and environmental hazards	Increased sense of safety, belonging, and confidence across diverse communities, improved ocean literacy
Workforce Development	• Provide cultural competency training for coastal public safety and resource protection staff; prioritize hiring and recruiting local personnel reflective of community demographics. • Create additional or expanded roles to advance environmental education and support sustainable use	Increased trust between communities and coastal agencies; improved equity and effectiveness of staffing
Coastal Infrastructure & Environmental Management	• Reduce parking fees or provide community vouchers or free lots • Improve affordable and accessible public transit to beaches and coastal areas • Build and maintain amenities that facilitate access and low-impact use for non-residents and people of all ages and abilities (i.e., trash receptacles, bathrooms, bike racks, picnic areas, ramps and pathways, etc.). • Mitigate run-off impacting urban, industrial, and/or agricultural coastal areas • Increase beach clean-ups, litter control, and habitat restoration efforts in neglected coastal areas	Increased physical access for all communities and reduced financial barriers; cleaner, healthier coastal environments
Participatory Governance	• Emphasize and broaden community consultation when designing, implementing, or modifying regulations affecting coastal use and access • Support community-based environmental sampling and monitoring programs • Facilitate Tribal co-stewardship of coastal areas and resources, including streamlined permits for ceremonial practices.	Increased regulatory awareness and compliance; prioritization of policy that addresses local needs and values
Funding & Grants	• Conduct equity audits and develop resource reallocation plans to increase investment in neglected coastal areas • Create or increase grant opportunities to facilitate coastal access, education, and engagement for underserved communities • Promote and/or subsidize affordable, high-density coastal housing options	More equitable distribution of resources to underserved communities

More broadly, our findings suggest that the primary crisis of ocean equity may no longer be solely about subsistence and economic rights, but the fundamental human need to connect to, belong in, and access restorative blue spaces^67^. This aligns with the concept of ‘realized access’ which suggests that socio-personal dimensions of accessibility are as important as legal rights^39^ and that cultural identity, social relationships, and personal resources fundamentally mediate the ability to derive benefits from ocean resources^46^. As compared to other regional research documenting barriers to urban greenspace access for historically marginalized and low-income populations^80,81^, fear of crime may not be a primary limiting factor in coastal access (i.e., most survey respondents reported feeling welcome in ocean and coastal spaces, though Supplementary Figs. 6 & 7 suggest this was somewhat less true for respondents of color) yet personal barriers related to transportation access and geographic distance appeared comparably acute. Consequently, if the primary value of the coast for vulnerable communities lies in social cohesion, mental health, and spiritual renewal, then management bodies must invest in Coastal Infrastructure & Environmental Management, which makes the use of local ocean areas for such purposes feasible, attractive, and affordable. In California specifically, agencies must prioritize more frequent and better integrated public transportation to pierce the coastal curtain and bridge the gap between isolated and/or urban communities and the coast. Where proximate shorelines are degraded or polluted, creative strategies may be required to remediate development, restore functional habitat, and transform neglected coastal fringes into community assets^80^.

In recent years many regional and national resource management agencies have made substantial (if episodic) progress in advancing recognitional equity by acknowledging historical injustice and the plurality of knowledge and value systems relevant to ocean management. Yet, distributional outcomes are likely to remain elusive without corresponding interventions designed to enhance procedural equity via Funding & Grants and Participatory Governance. Equitable ocean outcomes require moving beyond consultation models that treat community input as a social license toward embracing efforts to redistribute resources and decentralize decision-making power^2,3^. While the emerging focus on ocean equity is a positive development, environmental justice scholarship suggests that a sustainable and equitable future may ultimately require reckoning with the market-liberal logics and hierarchies through which bourgeois environmental organizations and the global ocean governance system derive their present authority^77,82^. Though social and environmental goals have traditionally been pursued in isolation, integrated approaches can help address the root causes, simultaneously fueling class exploitation, social inequality, and environmental degradation^83^. In this respect, global environmental change presents not only an existential threat for vulnerable coastal populations but also a rare opportunity for transformative change. With intentional design, coastal adaptation planning can be used as a lever to redress (rather than reproduce or exacerbate) historical inequalities^34^ and help build a future based upon universal ocean access, belonging, and wellbeing.

## Methods

All Human Subjects research was conducted following review and approval by the University of California, Santa Cruz Institutional Review Board (protocol #HS-FY2023-193). This study used a cross-sectional, community-engaged survey design; no statistical method was used to predetermine sample size. Overall, 2191 surveys were administered across California, including 1343 surveys administered in person (via paper or tablet) and 848 surveys administered online (via personal computer or cellphone). In-person survey respondents (engaged through tabling at community events or approached via intercept at coastal access points, laundromats, parks, and other public spaces) were compensated $20, while those taking the survey online (recruited through fliers, social media posts, and/or email lists) were entered in a lottery prize drawing. Using purposive sampling designed to ensure the inclusion of individuals who to-date have been underrepresented in ocean and coastal management processes, in-person survey efforts targeted disadvantaged and/or severely disadvantaged communities as defined by the California Environmental Protection Agency in three focal areas: Southeast San Francisco, the greater Monterey Bay, and Ventura & Oxnard counties (referred to here in aggregate as central California). To increase accessibility and broaden participation, the final survey instrument (iterated following an initial round of piloting) was translated and made available in Spanish and Chinese (simplified) in addition to English. Though not discussed in-depth in the context of the presented analysis, the survey instrument was developed and administered as part of a community-engaged research approach in which the research team partnered with local non-profit organizations (n = 5), dedicated to advancing coastal access and engagement and/or environmental justice to obtain feedback on survey content and design, engage respondents, contextualize and interpret research results, and co-develop management recommendations (via focus group discussions; n = 7). As this study is observational, the experiments were not randomized, and the Investigators were not blinded to allocation during experiments and outcome assessment. The complete, English-language version of the administered survey instrument is provided in the publicly accessible data and code repository^84^.

The sample used for analysis (*n* = 1691), was obtained following the sequential application of quality control measures to remove surveys that were: less than 60% complete (*n* = 262), completed in less than 400 seconds (*n* = 93, with this threshold determined as respondents who took the survey faster than 95% of other respondents), associated with respondents who did not live in California (*n* = 52), and those that did not meet a minimum response variability threshold (*n* = 86). The minimum variability threshold was assessed based on the five long-format (i.e., each question contained nine or more prompts), Likert-scale matrix questions contained within the survey. Responses from individuals selecting the same response for all the prompts contained within a question for 4 or more of the long-format questions did not meet the minimum response variability threshold. This procedure was developed upon observing in the field that a limited number of individuals, due to survey fatigue or lack of interest, appeared primarily motivated to complete the survey (and collect the incentive) and did not thoughtfully engage. Of the 1691 surveys included in the final analysis, the mean survey duration (i.e., the time that it took to complete the survey) was 19.2 min (median duration = 16.5 min).

All data processing, transformation, and exploration were conducted in R (version 4.4.1)^85^ using the ‘tidyverse packages’^86^, along with ‘sf’^87^ for the handling and visualizing spatial data, ‘tigris’^88^ for public Census Bureau base maps, and ‘rstatix’ for correlation testing^89^. Data obtained from demographic questions asking respondents to report race was transformed into a series of binary dummy variables to accommodate “Select all that apply” responses in which more than one category was chosen (i.e., a unique variable for ‘Hispanic or Latino’, in which responses including this category were marked as 1 and responses not including this category were marked as 0). Gender was assessed and coded similarly, though in this instance, related variables were mutually exclusive (following the logic that individuals who selected more than one category could, by definition, be characterized as non-binary). Categorical household income and frequency of ocean usage data were transformed into a 5-point ordinal scale with 1 representing the lowest income ($59,999 or less) and most infrequent usage (Less than once a year), and 5 representing the highest income ($240,000 or more) and most frequent usage.

Self-reported zip code of residence was used to derive 3 different geographic variables: community vulnerability, distance to ocean access, and region. Community vulnerability was assessed using CalEnviroScreen data^51^, a combined measure that employs a cumulative impact model in assessing community vulnerability as the product of environmental exposure and socioeconomic sensitivity. More specifically, a score assessed for each California census tract is based on a combination of Pollution Burden (i.e., Pesticide Use, Ozone, Hazardous Waste, Traffic Impacts, etc.) and Population Characteristics (i.e., Poverty, Unemployment, Housing Burden, Cardiovascular Disease, etc.) metrics. In our analysis, CalEnviroScreen census tract scores were aggregated at the zip code level (the lowest spatial resolution of the survey data), using population (i.e., # of total residents) weighted means. To match the format of other demographic variables, and account for the fact that the zipcodes with the highest CalEnviroScreen scores were agricultural areas far removed from the coast (i.e., the Central Valley), zip code-level scores were converted into an equal-interval (i.e., choosing breaks so that the total number of survey responses that fell into each category was approximately equal) 5 point ordinal scale (1 = least vulnerable; 5 = most vulnerable). Continuous distance to ocean access was calculated by using the shortest distance (in kilometers) between the geographic centroid of each ZIP code and the nearest Coastal Access Point (CAP), using ESRI ArcPro’s Closest Facility and Online Routing Services tool (as configured to perform road network analysis rather than Euclidean (straight-line) distance). CAPs included marine protected areas (MPAs), fishing piers and jetties, national marine sanctuaries (NMSs), and all designated coastal access points. The resulting continuous travel distance measurements were binned into a five-point ordinal scale (1 = least travel distance; 5 = most travel distance) consistent with the approach described above. Region was inferred based on the latitude of the geographic centroid associated with each ZIP code as parsed by the following cut-off values: latitude ≥ 37.25° = Northern Region; latitude <37.25° & > 35.8° = Central Region; and latitude ≤ 35.8° = Southern Region. For the correlation (i.e., Fig. 3) and ordinary least squares regression analyses (i.e., Fig. 5 & Fig. 7) this parameter was transformed into a series of dummy variables to match the format of other data (see above) and facilitate scaling enabling relative comparison.

Long-format, Likert-scale matrix questions (each containing 9 prompts with which respondents were asked to indicate their level of agreement using the (5) categories Strongly Disagree, Disagree, Neither agree nor disagree, Agree, or Strongly Agree) were used to assess barriers to ocean access and the contribution of the ocean to human wellbeing. For each parameter (i.e., Barriers and Ocean Wellbeing) a composite index was derived by finding the mean response value per respondent following the transformation of the Likert-scale into numerical values (i.e., Strongly Agree = 5, Agree = 4, etc.). Dimensional subscales for each parameter were subsequently constructed following the same procedure. The prompts included in each scale and sub-scale were iteratively developed and assigned using a) a review of relevant literature (see Supplementary Table 1 and Supplementary Table 2), b) initial scoping discussions, designed to explore issues and dynamics of local relevance across our study area, conducted with community partner organizations and other local leaders (*n* = 10); and, c) feedback obtained during survey piloting. As many aspects of ocean access and wellbeing are best considered as part of a multidimensional continuum (rather than as discrete categories)^39^, in certain instances, responses to individual prompts were considered in more than one sub-scale

Differences between sub-scales were assessed by grouping individual, complete responses (*n* = 1600 for Barriers prompts; n = 1568 for Wellbeing prompts), using a repeated measures ANOVA (which tests for the significant differences between the means of three or more groups, where the same subjects are measured under each condition) and a post-hoc Tukey HSD test (performed to identify exactly which pairs are different while controlling for the family-wise error rate)^90^. Reliability of each scale and subscale was assessed using both Cronbach’s Alpha (α) and McDonald’s Omega (ω_t_). Cronbach’s alpha was assessed as:

$$\alpha=\frac{k}{k-1}\left(1-\frac{{\sum }_{i=1}^{k}{\sigma }_{i}^{2}}{{\sigma }_{{{\rm{total}}}}^{2}}\right)$$

where $k$ is the number of items contributing to the scale, ${\sigma }_{i}^{2}$ is the variance of item $i$, and ${\sigma }_{{{\rm{total}}}}^{2}$ is the variance of the total scale score. Generally speaking, values ≥ 0.80 indicate a scale has very good reliability, values < 0.8 and ≥ 0.7 indicate good reliability, values < 0.7 and ≥ 0.6 indicate acceptable (but weak) reliability, values < 0.6 and ≥ 0.5 indicate questionable reliability (which may be acceptable in exploratory research), and values < 0.5 indicate poor reliability (i.e. the scale does not measure a coherent construct). McDonald’s omega was assessed as:

$${\omega }_{t}=\frac{{\left(\mathop{\sum }_{i=1}^{k}{\lambda }_{i}\right)}^{2}}{{\left(\mathop{\sum }_{i=1}^{k}{\lambda }_{i}\right)}^{2}+\mathop{\sum }_{i=1}^{k}{\psi }_{i}}$$

where ${\lambda }_{i}$ is the factor loading of item $i$ on the general factor and ${\psi }_{i}$ is the corresponding unique (residual) variance. As compared to Cronbach’s alpha, McDonald’s omega is considered more accurate as applied to a limited number of scale items (i.e., dimensional sub-scales) and/or instances where factor loadings may be unequal. The quality of each prompt and subscale was assessed using Item-Total correlations (the correlation between an item’s score and the sum of all item scores, including the item itself), Item-Rest correlations (the correlation between the item and the total score of the scale excluding that item), and their corresponding means and standard deviations. These metrics are useful in providing measurements of discrimination as items and/or prompts with the highest mean values are not necessarily the most informative if they are measuring a general concept that everyone uniformly agrees with^91^. Reliability metrics (Cronbach’s α, McDonald’s ω) and item-level statistics (item-total and item-rest correlations) were computed using the R **‘**psych’ package^92^.

In order to examine the relationship between our derived indices and geographic (i.e., region and distance (km) to ocean access), gender, race, and socioeconomic (i.e., community vulnerability and household income) variables, we used Ordinary Least Squares (OLS) regression (a method to estimate the linear coefficients of our predictors by minimizing the sum of squared residuals between the observed and predicted values)^90^. For each respondent $i=1,\ldots,n$, the regression model takes the form:

$${Y}_{i}={\beta }_{0}+\mathop{\sum }_{j=1}^{k}{\beta }_{j}{X}_{{ij}}+{\varepsilon }_{i}$$

where ${Y}_{i}$ is the standardized scale score for respondent $i$, ${X}_{{ij}}$ is the value of predictor $j$ for respondent $i$, ${\beta }_{j}$ is the regression coefficient for predictor $j$, ${\beta }_{0}$ is the intercept, and ${\varepsilon }_{i}$ is the residual error. Before beginning modeling, we used a pairwise correlation matrix to identify multicollinearity. Given the strong negative correlation between the Male and Female (r = −0.93, p < 0.001) binary variables, the pair was assessed as providing redundant information, leading us to permute which of the two was included in each model and present the alternate version in the supplement. All independent variables were scaled prior to model construction in order to facilitate the comparison (i.e., standardization) of model coefficients. Models were assembled through forward, stepwise selection in which variables were added one at a time, and nested models were compared using Akaike Information Criteria (Supplementary Table 3). The final models were limited to data from those survey respondents who chose to provide complete demographic information and responded to all relevant prompts (i.e., *n* = 1237 for wellbeing models; *n* = 1255 for barriers models). We explored using linear mixed models (implemented using the R package ‘lme4’^93^) and representing region as a random effect, but in all instances, model fit was improved by including region as a categorical (i.e., factor) variable with the Central region serving as the reference level. Consistent with standard practice in regression-based analyses with a pre-specified set of theoretically motivated predictors, p-values for individual coefficients are reported without adjustment for multiple comparisons; coefficients within each model test conceptually distinct, non-redundant questions about partial effects^94^. Models were validated by graphically examining residuals using histograms to verify normality and standardized residuals vs fitted values plots to inspect variance structure. The final regression tables for all models and sub-models can be accessed online through the repository linked below in the Data and code availability section.

### Reporting summary

Further information on research design is available in the Nature Portfolio Reporting Summary linked to this article.

## Supplementary information

Supplementary Information

Reporting Summary

Transparent Peer Review file

## Data Availability

Data and metadata used to produce the primary analyses^84^ can be accessed from the following github repository: https://github.com/thfrawley/Ocean_Barriers_and_Wellbeing.

## Code availability

Original code used to produce the primary analyses^84^ can be accessed from the following github repository: https://github.com/thfrawley/Ocean_Barriers_and_Wellbeing.
