# Supplementary material for: Racial and economic disparities in coastal access and engagement mediate the ocean’s contribution to human wellbeing
Source: Nat Commun. 2026 Jul 13;17:5975. doi: 10.1038/s41467-026-75034-4 (PMC13365518; doi:10.1038/s41467-026-75034-4)
Supplement: Supplementary file 1 — Supplementary Information [file 41467_2026_75034_MOESM1_ESM.pdf]

Racial and Economic Disparities in Coastal Access and Engagement Mediate the Ocean's Contribution to Human Wellbeing

SUPPLEMENTARY INFORMATION

Supplementary Table 1. Summary of the theoretical frameworks informing the study's conceptualization of barriers to ocean access. Responses to survey prompts were measured on a 5-point likert scale (Strongly Disagree = 1; Disagree = 2; Neutral = 3; Agree = 4; Strongly Agree = 5).

	**Category**	**Definition**	**Sub-Categories**	**Survey Prompts**
Barriers to Ocean Access<br><br>Key References:<br><br>Wang et al., 2015<br>Byrne et al., 2009	Social	Societal norms, historical practices, and cultural dynamics that influence whether individuals are comfortable or motivated to engage with coastal and ocean spaces.	Feelings of Safety, Welcomeness, & Belonging; Existence of Shared Activities, Social Exclusion or Marginalization	• I am interested in ocean experiences or activities<br>• I feel welcome at beaches and coastal areas<br>• I feel protected by law or rule enforcers (e.g. police, rangers, security guards)
	Knowledge	Gaps in information, education, or skills that prevent people from safely and legally accessing and engaging with coastal and ocean environments	Understanding Rules & Regulations, Possessing Necessary Skills or Abilities, Awareness of and Familiarity with Access Points	• I understand the associated rules and regulations (e.g., fishing rules, parking rules)<br>• I feel safe from environmental factors (i.e. sharks, waves, wind, pollution, etc.)<br>• I possess the required skills or knowledge to participate in recreational activities in or by the ocean (i.e., swimming, fishing, surfing)
	Physical-Environmental	Tangible, structural, or environmental obstacles that directly impede the ability to access, use, or safely enjoy coastal and ocean spaces	Availability of Amenities and Infrastructure; Pollution and/or Environmental Degradation; Exposure to Hazardous Natural Conditions, Presence of Threatening Wildlife	• The amenities/infrastructure I need or want are available, clean, and in good condition (i.e., restrooms, wheelchair ramps, picnic tables, piers, etc.)<br>• I feel safe from environmental factors (i.e. sharks, waves, wind, pollution, etc.)<br>• I have access to and can afford transportation to the beach or coasts (e.g., bus, car)
	Personal	Individual socioeconomic or geographic attributes that constrain time spent in ocean environments or participating in associated activities	Transport, Proximity, Affordability, Availability of Leisure Time, Interest & Lifestyle	• I am interested in ocean experiences or activities<br>• I can afford or have access to necessary tools, gear, or equipment (e.g., fishing pole, surfboard).<br>• I have access to and can afford transportation to the beach or coasts (e.g., bus, car)

Supplementary Table 2. Summary of the theoretical frameworks informing the study's conceptualization of the ocean's contribution to human well-being. Responses to survey prompts were measured on a 5-point likert scale (Strongly Disagree = 1; Disagree = 2; Neutral = 3; Agree = 4; Strongly Agree = 5).

	**Category**	**Definition**	**Sub-Categories**	**Survey Prompts**<br>(*California's oceans and coasts…*)
The Ocean's Contribution to Human Wellbeing<br><br><u>Key References</u>:<br><br>Allison et al., 2020<br>Weeratunge et al., 2014<br>White 2010	Material	The ocean as a means to an end, an asset, satisfaction of needs and preferences, usefulness for people	Income; Food, Energy & Materials; Ecosystem Creation & Maintenance, Physical Health	• are an important source of food for myself and/or my family<br>• provide me with income or a job<br>• improve and/or maintain my physical health and/or mental well-being<br>• allow me to participate in adventurous and exciting activities
	Relational	The ocean as a medium that aids the relationships people have with themselves, the natural world and communities and cultures of which they are a part	Group Identity & Social Cohesion, Intra-household and Intra-community Relations, Local Knowledge & Way of Life, Culture & Tradition	• help me to build or maintain relationships with my friends or family<br>• support plants, animals and/or activities that are important to my culture and heritage<br>• help me to feel a part of a community
	Subjective	Personal satisfaction or emotional affect derived from the ocean, the inherent worth people assign to the existence of marine life and/or coastal spaces and environments as ends in of themselves	Mental Health & Well-being, Individual Identity, Aesthetics & Awe, Spirituality & Symbolism; Biodiversity, Animal Rights & Welfare	• help me to feel more spiritual and/or connected to a higher power<br>• allow me to participate in adventurous and exciting activities<br>• improve and/or maintain my physical health and/or mental well-being<br>• expose me to beauty and/or sensory experiences (e.g., wind, light, smell, sound) that are important to me.

Supplementary Table 3. Example of forward, stepwise model selection procedure used to construct the OLS regression model where the Ocean Wellbeing Index served as the dependent variable. Nested models were compared using AIC criteria. The final model used in the analysis is shown in bold.

Model	AIC
WB_Index~C_Vulnerability	2485.3
WB_Index~C_Vulnerability + Income	2486.5
WB_Index~C_Vulnerability*Income	2488.5
WB_Index~Income	2465.1
WB_Index~C_Vulnerability+White+Asian+Black+Pacific_Islander+Native+Latino	2464.9
WB_Index~C_White+Asian+Black+Pacific_Islander+Native+Latino	2466.2
WB_Index~C_Vulnerability+White+Asian+Black+Pacific_Islander+Native+Latino + (1|Area)	2527.8
WB_Index~C_Vulnerability+White+Asian+Black+Pacific_Islander+Native+Latino + Area	2464.3
WB_Index~C_Vulnerability + White+Asian+Black+Pacific_Islander+Native+Latino + Area+ Male + Non-binary	2467.9
**WB_Index~C_Vulnerability + White+Asian+Black+Pacific_Islander+Native+Latino + Area + Distance**	**2456.1**

Supplementary Table 4. Item-Total Correlation and Item Rest-Correlation values quantifying the quality of each prompt in assessing the core construct measured by the composite barriers scale (black values) and each barrier sub-scale (colored values).

	Sub-Scale	Prompt	#	Item-Total Correlation (Composite Scale)	Item-Total Correlation		Item-Rest Correlation (Composite Scale	Item-Rest Correlation (Sub-Scale)	
Barriers to Ocean Access		I feel welcome at beaches and coastal areas	2	0.642	0.801		0.549		0.548
	Social	I feel protected by law or rule enforcers (e.g. police, rangers, security guards)	9	0.579	0.710		0.436		0.229
		I am interested in ocean experiences or activities	1	0.541	0.680	0.685	0.429	0.394	0.337
	Personal	I can afford or have access to necessary tools, gear, or equipment (e.g., fishing pole, surfboard)	6	0.704		0.794	0.582	0.412	
		I have access to and can afford transportation to the beach or coasts (e.g., bus, car)	3	0.662	0.682	0.802	0.560	0.547	0.365
	Physical-Environ.	The amenities/infrastructure I need or want are available	4	0.594	0.782		0.446		0.422
		I feel safe from environmental factors (i.e. sharks, waves, wind, pollution, etc.)	7	0.699	0.753	0.799	0.587	0.528	0.410
	Knowledge	I possess the required skills or knowledge to participate in ocean activities (i.e., swimming, fishing, surfing)	5	0.695		0.812	0.580	0.544	
		I understand the associated rules and regulations (e.g., fishing rules, parking rules)	8	0.712		0.801	0.617	0.575	

Supplementary Table 5. Item-Total Correlation and Item Rest-Correlation values quantifying the quality of each prompt in assessing the core construct measured by the composite wellbeing scale (black values) and each wellbeing sub-scale (colored values).

	Sub-Scale	Prompt	#	Item-Total Correlation (Composite Scale)	Item-Total Correlation (Sub-Scale)		Item-Rest Correlation (Composite Scale)	Item-Rest Correlation (Sub-Scale)	
Ocean's Contribution to Human Wellbeing	Material	are an important source of food for myself and/or my family	1	0.575	0.728		0.403	0.377	
	Material	provide me with an income or a job	2	0.460	0.652		0.267	0.261	
	Material / Subjective	allow me to participate in adventurous and/or exciting activities	6	0.678	0.661	0.758	0.581	0.413	0.561
	Material / Subjective	improve and/or maintain my physical health and/or mental well-being	7	0.709	0.604	0.839	0.632	0.376	0.720
	Subjective	help me to feel more spiritual and/or connected to a higher power	8	0.679		0.782	0.554		0.534
	Subjective	expose me to beauty and/or sensory experiences (e.g., wind, light, smell, etc.) that are important to me	9	0.640		0.797	0.548		0.652
	Relational	help me to feel a part of a community	3	0.739	0.836		0.646	0.612	
	Relational	help me build or maintain relationships with my friends or family	4	0.723	0.816		0.632	0.592	
	Relational	support plants, animals and/or activities that are important to my culture and heritage	5	0.667	0.764		0.547	0.449	

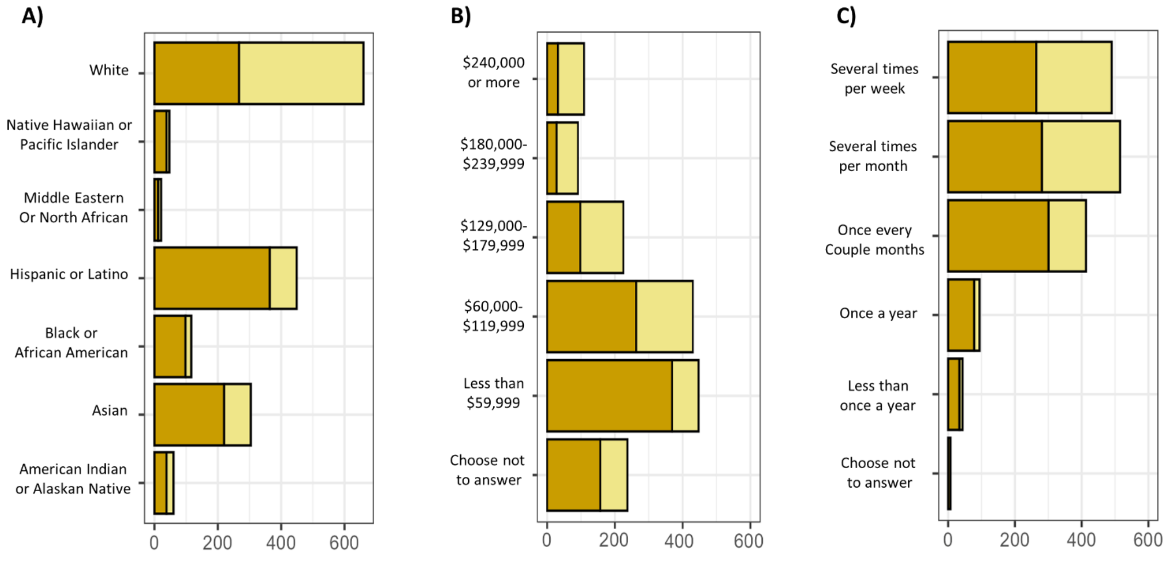

Supplementary Figure 1. Frequency-distribution barplots illustrate the distribution of survey respondents (n = 1691 California residents) by A) racial-ethnic categories, B) annual household income, and C) frequency of use of ocean and coastal areas. The y-axis in each panel shows the count of respondents in that category. Bars are color-coded according to the format of survey administration (dark orange = in-person administration, n=1074; yellow = online administration, n=617). Because each bar represents a count of respondents in a discrete category rather than an aggregate of replicate measurements, individual data points are not overlaid. No statistical comparisons are reported for these distribution plots.

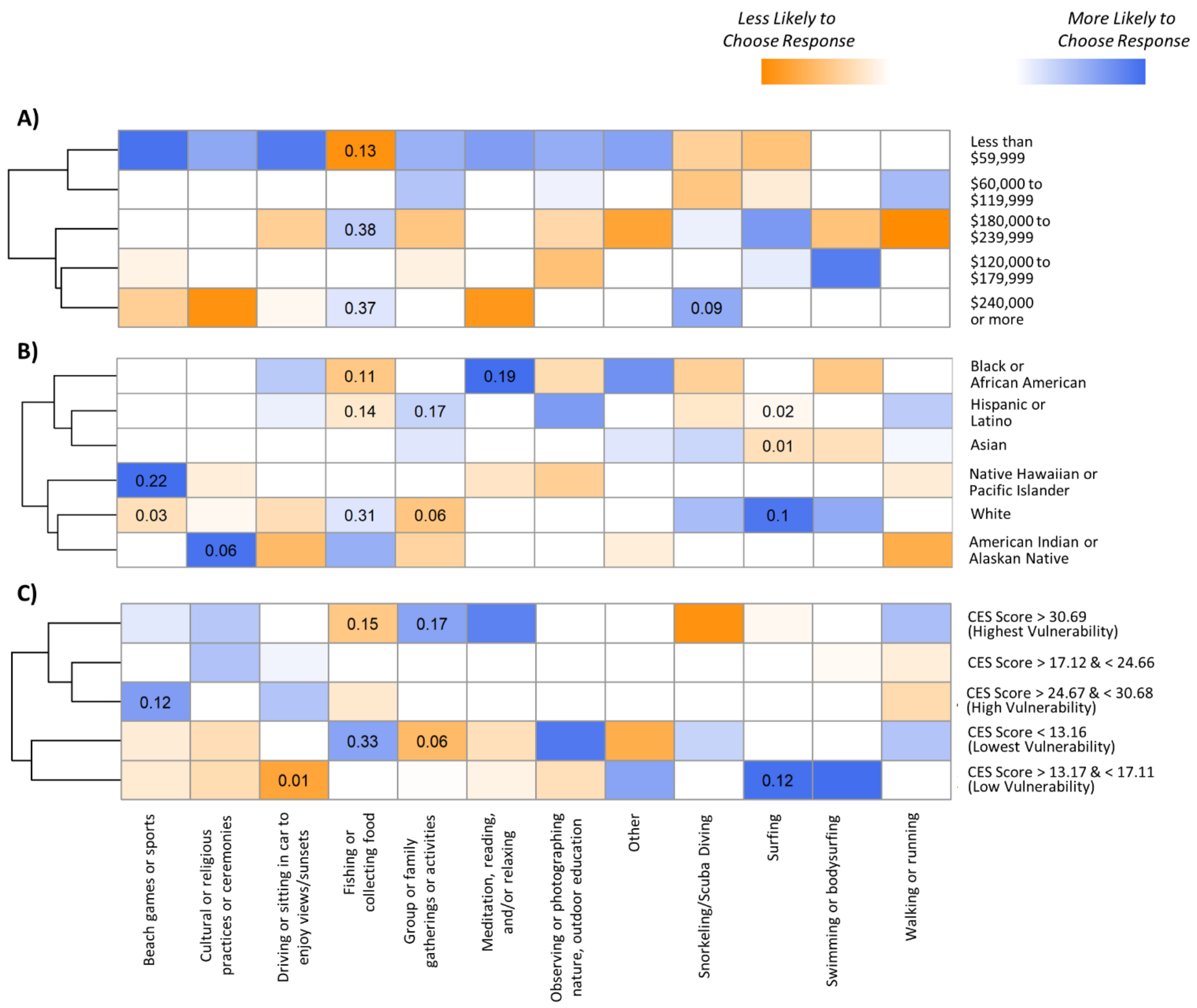

Supplementary Figure 2. Heatmaps illustrating the ocean activities that survey respondents (n=1691) choose as the most important to them, as compared across **A**) household income, **B**) race-ethnicity, and **C**) community vulnerability categories. Heatmaps were constructed from cells defined by proportions (i.e., # of respondents in category selecting activity/total number of respondents in category). Color scale: orange shading indicates proportions lower than the panel average; blue shading indicates proportions higher than panel average; intensity scales with the magnitude of deviation. Only those categorical proportions significantly different from the total proportion (respondents selecting activity/ total number of respondents), as determined by a two-sided z-test following a Bonferroni correction to control for the family-wise error rate, are labeled. All displayed labels correspond to $p < 0.05$ after Bonferroni adjustment. The rows of each panel are clustered using the complete linkage agglomeration method while the color-mapped values of each panel are scaled to facilitate relative comparison (i.e., those proportions higher or lower than average).

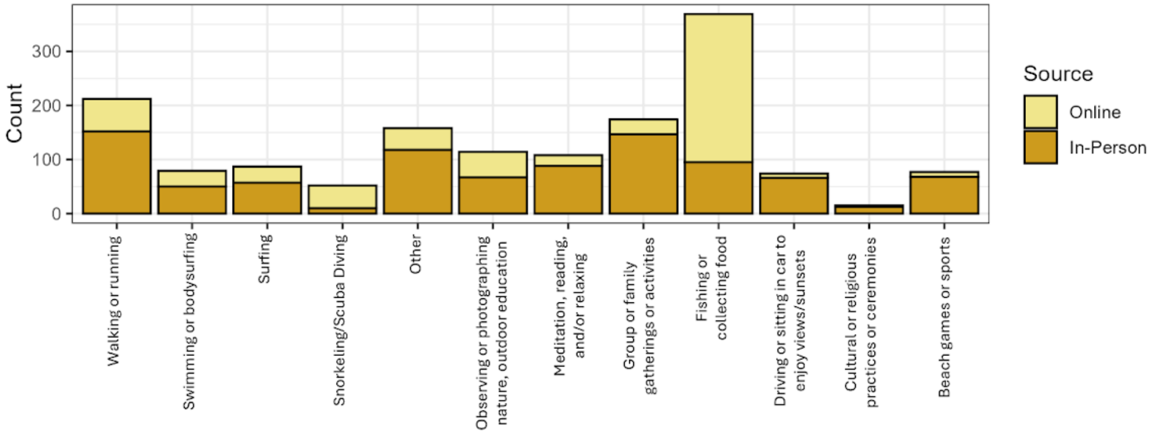

Supplementary Figure 3. Frequency-distribution barplot illustrating the distribution of survey respondents by the ocean activity they choose as most important to them. The y-axis shows the count of respondents. Bars are color-coded according to the format of survey administration (dark orange = in-person administration, n=1074; yellow = online administration, n=617). As each bar represents a count of respondents within a category rather than aggregated replicate measurements, individual data points are not overlaid. No statistical comparisons are reported for this distribution plot.

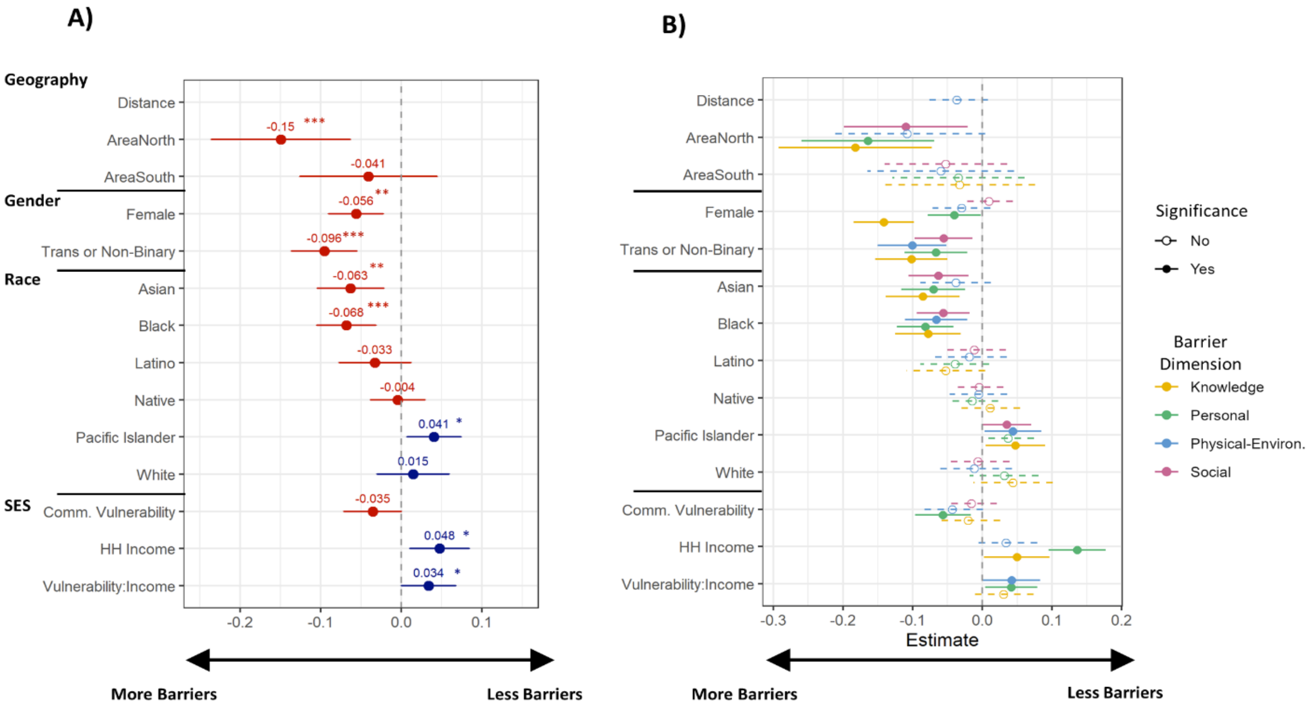

Supplementary Figure 4. Alternate version of main text Figure 5 in which one collinear variable (Male) is replaced by another (Female) in the final models. As in Figure 5, points represent the standardized regression coefficient estimate for each predictor (the measure of centre), and error bars represent the 95% confidence interval around the estimate. Statistical significance for each coefficient was assessed using two-sided t-tests with no adjustment for multiple comparisons (see Methods). Sample size: n = 1,255 respondents with complete demographic and barrier-prompt data. Coefficient estimates, standard errors, and exact p-values are provided in the regression tables linked in the Data Availability section.

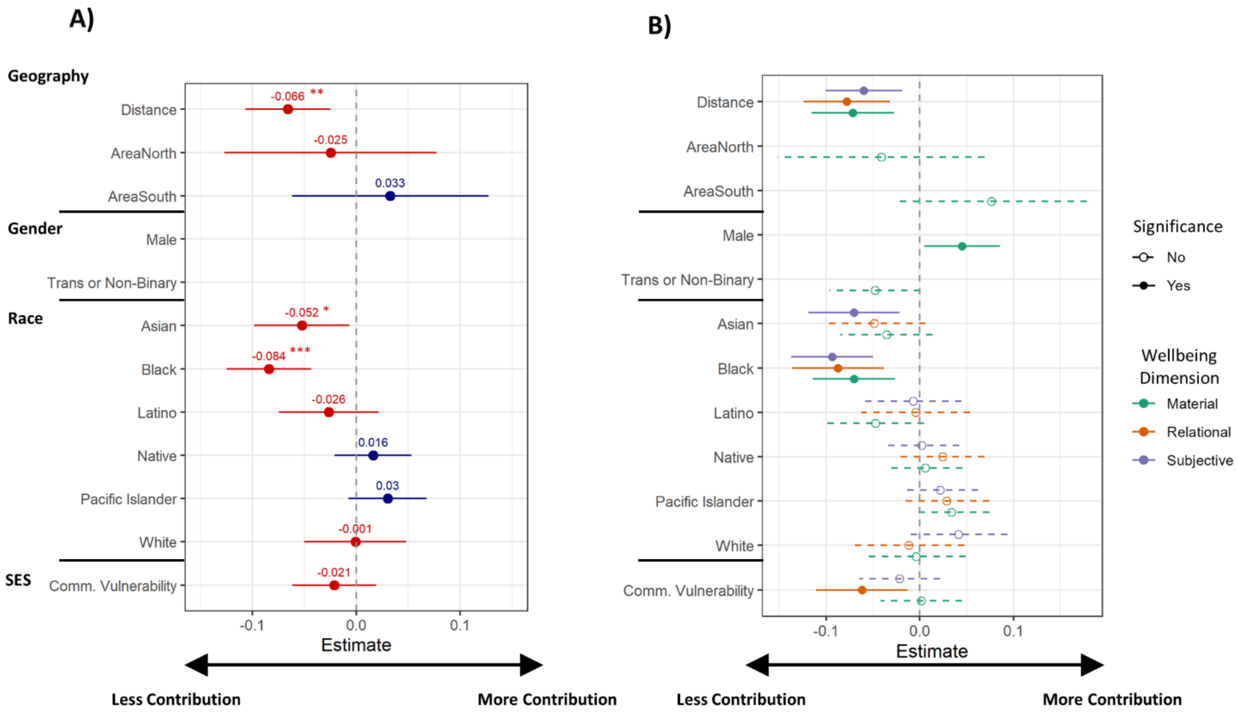

Supplementary Figure 5. Alternate version of main text Figure 7 in which one collinear variable (Female) is replaced by another (Male) in the final models. As in Figure 7, points represent the standardized regression coefficient estimate for each predictor (the measure of centre), and error bars represent the 95% confidence interval. Statistical significance was assessed using two-sided t-tests with no adjustment for multiple comparisons (see Methods). Sample size: n = 1,237 respondents with complete demographic and wellbeing-prompt data. Coefficient estimates, standard errors, and exact p-values are provided in the regression tables linked in the Data Availability section.

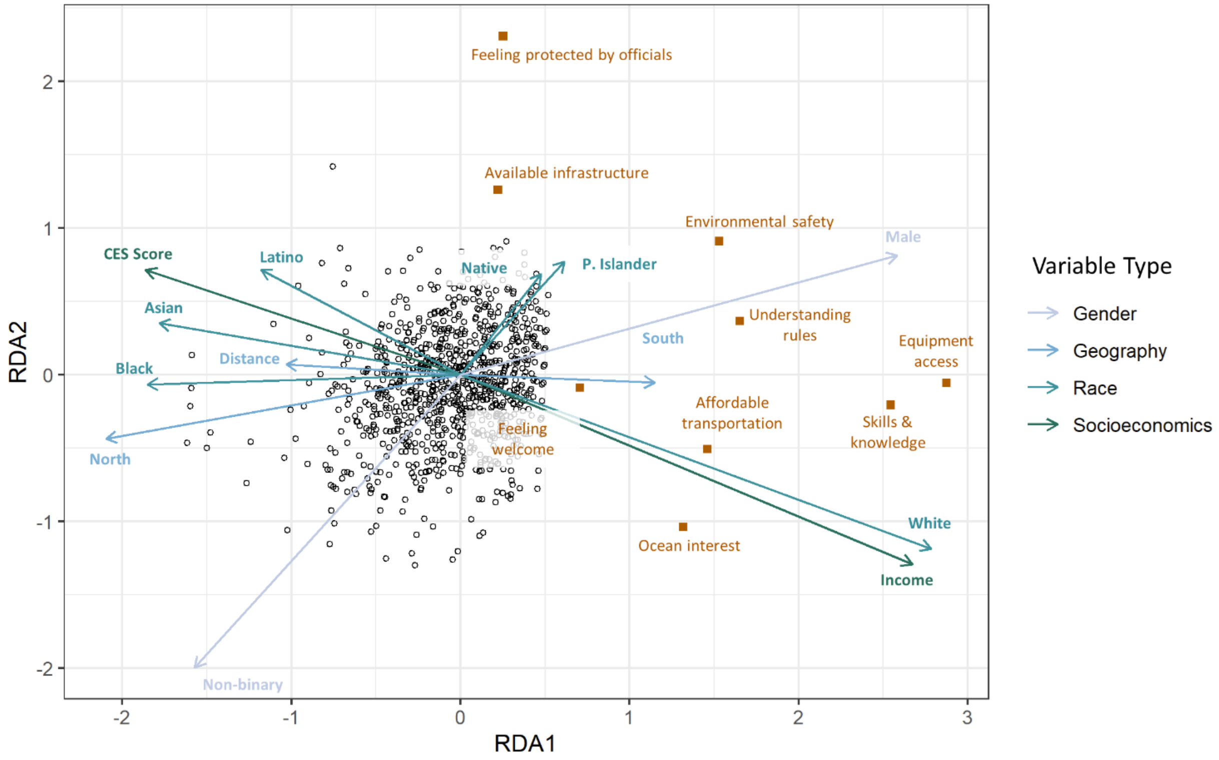

Supplementary Figure 6. Redundancy analysis used to explore the degree to which gender, race, socioeconomic and geographic variables explained variation in reported barriers (as specified at the level of individual prompts). Sample size: n = 1,255 respondents with complete data. The full RDA model was statistically significant ($F = 7.57$, df = 13 and 1,241, $p = 0.001$; 999 free permutations). Barrier prompts, which formed the basis of the response matrix, are shown in brown, while predictor variables are shown in blue-green (shaded according to variable type). Longer arrows indicate stronger association, with smaller angles between arrows (<90° or >270°) indicating positive correlation, right angles (90°) indicating little or no correlation, and opposite arrows (180° apart) indicative of negative correlation. The position of a point relative to an arrow shows how strongly the reported existence of a barrier is positively associated with a given variable; with barriers in the opposite quadrant of arrows negatively associated. Barriers located close to the origin are overall weakly associated with predictor variables; barriers (i.e., points) located in the same quadrant are associated with similar variables. Significant predictors ($p < 0.05$) identified by sequential (type I) permutation tests, listed in order of effect size: Income ($F = 25.99$, $p = 0.001$), Male gender ($F = 17.50$, $p = 0.001$), North region ($F = 13.90$, $p = 0.001$), Asian identity ($F = 7.51$, $p = 0.001$), Black identity ($F = 7.36$, $p = 0.001$), non-binary gender ($F = 7.30$, $p = 0.001$), Latino identity ($F = 5.78$, $p = 0.001$), community vulnerability (CES Score: $F = 5.67$, $p = 0.002$), and Pacific Islander identity ($F = 2.43$, $p = 0.044$).

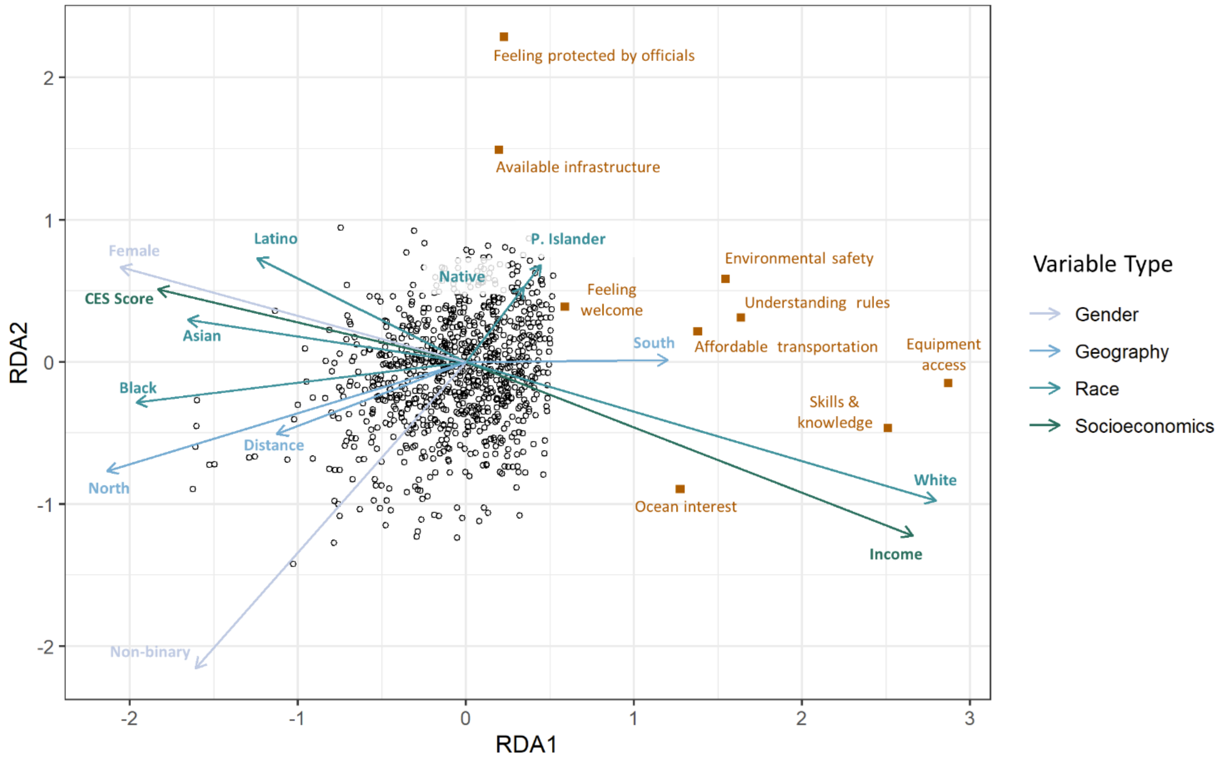

Supplementary Figure 7. Alternate version of Supplementary Figure 6 in which one collinear variable (Male) is replaced by another (Female) in the final model. Sample size: n = 1,255 respondents with complete data. The full RDA model was statistically significant (F = 7.42, df = 13 and 1,241, p = 0.001; 999 free permutations). Significant predictors ($p < 0.05$) identified by sequential (type I) permutation tests, listed in order of effect size: Income (F = 25.95, p = 0.001), North region (F = 13.88, p = 0.001), non-binary gender (F = 11.59, p = 0.001), Female gender (F = 11.37, p = 0.001), Asian identity (F = 7.49, p = 0.001), Black identity (F = 7.35, p = 0.001), Latino identity (F = 5.77, p = 0.001), community vulnerability (CES Score: F = 5.66, p = 0.001), and Pacific Islander identity (F = 2.43, p = 0.044).

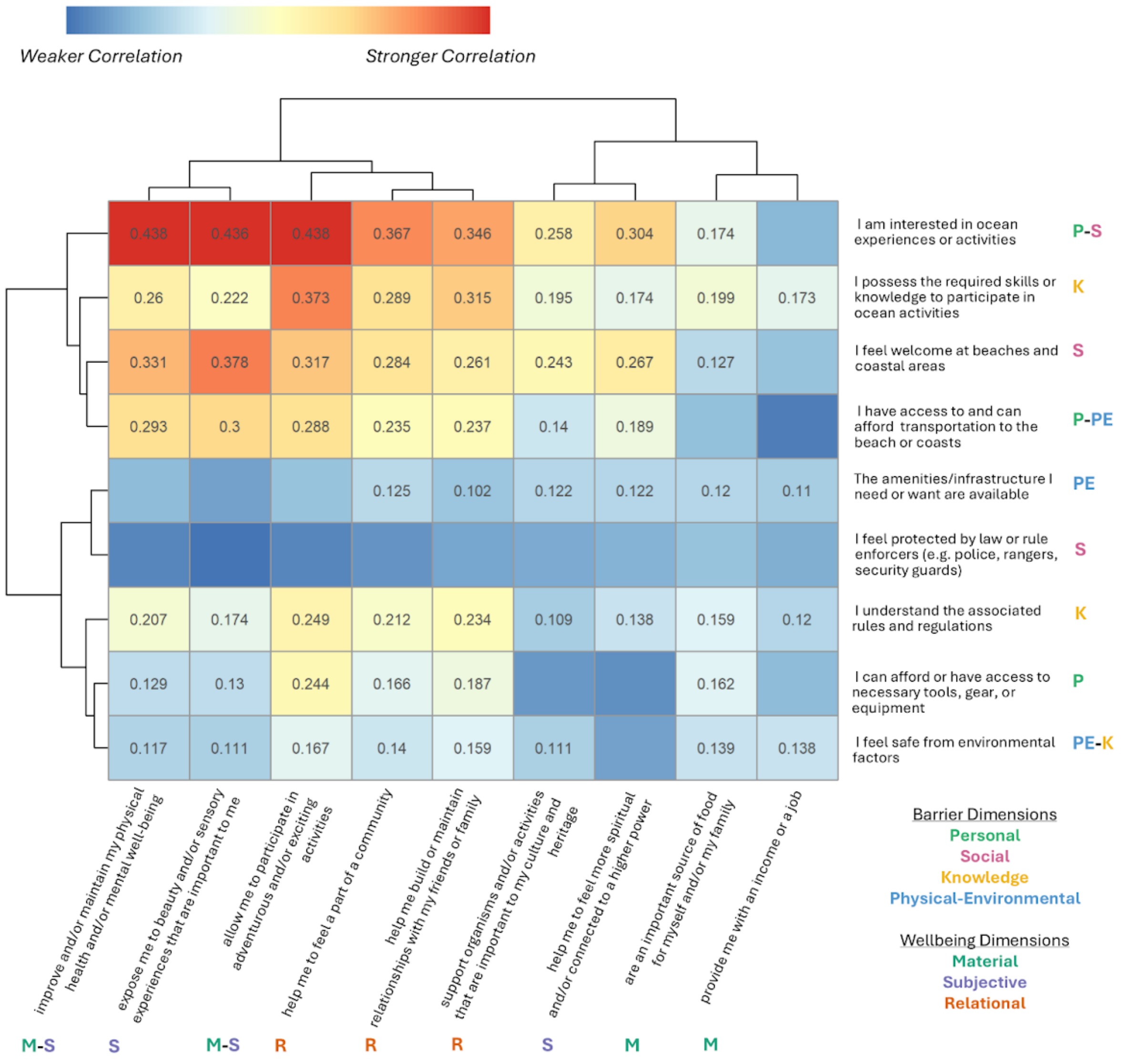

Supplementary Figure 8. Heatmap comparing the strength of association between the Barriers prompts and Wellbeing prompts (n=1691 survey respondents). Numerical labels represent the Spearman rank correlation coefficient values that were the basis for comparison, with only values corresponding to highly significant comparisons ($p < 0.01$, following the application of a Bonferroni adjustment to correct for the family-wise error rate) displayed. Rows and columns are independently ordered using complete-linkage hierarchical clustering on Euclidean distances of the correlation values. Colored letters below or to the side of each prompt are used to indicate the dimensional sub-scale to which each prompt corresponds. Red cell colors represent stronger correlations and blue cell colors represent weaker correlations. Generally speaking, red cells positioned at the top-left of the heatmap are indicative of strong relationships through which a specific barrier functions to limit a specific wellbeing attribute. Conversely, blue cells positioned to the bottom-right of the plot (or not displaying a correlation value due to lack of significance) are indicative of barriers that did not have a strong impact on specific wellbeing attributes.
