# Supplementary material for: Racial and economic disparities in coastal access and engagement mediate the ocean’s contribution to human wellbeing
Source: Nat Commun. 2026 Jul 13;17:5975. doi: 10.1038/s41467-026-75034-4 (PMC13365518; doi:10.1038/s41467-026-75034-4)
Supplement: Supplementary file 2 — Reporting Summary [file 41467_2026_75034_MOESM2_ESM.pdf]

nature portfolio

Corresponding author(s): Timothy Haight Frawley

Last updated by author(s): May 14, 2026

Reporting Summary

Nature Portfolio wishes to improve the reproducibility of the work that we publish. This form provides structure for consistency and transparency in reporting. For further information on Nature Portfolio policies, see our Editorial Policies and the Editorial Policy Checklist.

Statistics

For all statistical analyses, confirm that the following items are present in the figure legend, table legend, main text, or Methods section.

n/a	Confirmed	
☐	☒	The exact sample size (*n*) for each experimental group/condition, given as a discrete number and unit of measurement
☐	☒	A statement on whether measurements were taken from distinct samples or whether the same sample was measured repeatedly
☐	☒	The statistical test(s) used AND whether they are one- or two-sided<br>*Only common tests should be described solely by name; describe more complex techniques in the Methods section.*
☐	☒	A description of all covariates tested
☐	☒	A description of any assumptions or corrections, such as tests of normality and adjustment for multiple comparisons
☐	☒	A full description of the statistical parameters including central tendency (e.g. means) or other basic estimates (e.g. regression coefficient) AND variation (e.g. standard deviation) or associated estimates of uncertainty (e.g. confidence intervals)
☐	☒	For null hypothesis testing, the test statistic (e.g. *F*, *t*, *r*) with confidence intervals, effect sizes, degrees of freedom and *P* value noted<br>*Give P values as exact values whenever suitable.*
☒	☐	For Bayesian analysis, information on the choice of priors and Markov chain Monte Carlo settings
☒	☐	For hierarchical and complex designs, identification of the appropriate level for tests and full reporting of outcomes
☐	☒	Estimates of effect sizes (e.g. Cohen's *d*, Pearson's *r*), indicating how they were calculated

Our web collection on statistics for biologists contains articles on many of the points above.

Software and code

Policy information about availability of computer code

Data collection	Qualtrics software was used as an online survey tool to facilitate the distribution of surveys and the collection of responses.
Data analysis	All statistical analyses were conducted in R (version 4.4.1). Data processing, transformation, exploration, and visualization used the tidyverse packages; spatial data were handled with the sf package; correlation testing (including Bonferroni-corrected pairwise p-values) used rstatix; scale reliability metrics (Cronbach's α, McDonald's ω) and item-level statistics (item-total and item-rest correlations) were computed with the psych package; linear mixed-effects models examined during model selection were fit with the lme4 package. Distance from ZIP code centroids to the nearest coastal access point was computed in ESRI ArcPro using its Closest Facility tool, configured for road-network (not Euclidean) routing. All analytical R scripts and source data are publicly available at https://github.com/thfrawley/Ocean_Barriers_and_Wellbeing.

For manuscripts utilizing custom algorithms or software that are central to the research but not yet described in published literature, software must be made available to editors and reviewers. We strongly encourage code deposition in a community repository (e.g. GitHub). See the Nature Portfolio guidelines for submitting code & software for further information.

Data

Policy information about availability of data

All manuscripts must include a data availability statement. This statement should provide the following information, where applicable:

- Accession codes, unique identifiers, or web links for publicly available datasets
- A description of any restrictions on data availability
- For clinical datasets or third party data, please ensure that the statement adheres to our policy

Data and metadata used to produce the primary analyses can be accessed from the following github repository: https://github.com/thfrawley/Ocean_Barriers_and_Wellbeing

Research involving human participants, their data, or biological material

Policy information about studies with human participants or human data. See also policy information about sex, gender (identity/presentation), and sexual orientation and race, ethnicity and racism.

Reporting on sex and gender	Gender (as shaped by social and cultural circumstances) data collected and analyzed in our study was obtained via self-reported characterization recorded as a response to one of the demographic questions in the administered survey instrument. In Q25, respondents were asked to check all that apply and given the choices a) Male; b) Female; c) Transgender, non-binary, or another gender; or d) Choose Not to Answer. This information was coded into a series of dummy variables mutually exclusive dummy variables for Male, Female, and Transgender or Non-Binary (following the logic that individuals who selected more than one category could, by definition, be characterized as non-binary). This information was collected and analyzed with written consent (all survey respondents were required to complete a consent form prior to the imitation of survey administration and where given the freedom to opt out of any questions they did not feel comfortable answering following procedures outlined in the UC Santa Cruz Institutional Review Board approved Human Subjects Research protocol; protocol #HS-FY2023-193).
Reporting on race, ethnicity, or other socially relevant groupings	Data obtained from demographic questions asking respondents to report race was transformed into a series of binary dummy variables to accommodate "Select all that apply" responses in which more than one category was chosen (i.e., a unique variable for 'Hispanic or Latino", in which responses including this category were marked as 1 and responses not including this category were marked as 0). Categorical household income was based self-reported information as assessed through another demographic questions. Range values (see survey instrument attached as Supplemental Information) were transformed into a 5 point ordinal scale with 1 representing the lowest income ($59,999 or less) bracket and 5 representing the highest income ($240,000 or more) bracket. Community Vulnerability Status was assessed by linking self-reported Zipcode of primary residence (as assessed by a demographic survey question) with CalEnviroScreen scores associated with that Zipcode through previously established research methodology (see Methods text for additional information).
Population characteristics	See below; detailed population characteristics including racial/ethnic composition, gender distribution, age, household income, and community-vulnerability designation are provided in the Behavioural & social sciences study design (Research sample) and in the manuscript main text (Results, opening paragraph; Supplementary Figure 1).
Recruitment	In-person survey respondents (engaged through tabling at community events or approached via intercept at coastal access points, laundromats, parks, and other public spaces) were compensated $20, while those taking the survey online (recruited through fliers, social media posts, and email lists) were entered in a lottery prize drawing. This recruitment approach introduces several potential biases: voluntary participation may over-represent individuals with stronger pre-existing engagement with ocean topics, in-person intercept recruitment may over-represent those active in community spaces, and online recruitment is subject to digital-divide bias. These biases are partially mitigated by the multi-mode administration approach, purposive sampling of historically underrepresented communities, and community-partner recruitment. Findings characterize an engaged sample rather than the California general population.
Ethics oversight	All Human Subjects research was conducted following review and approval by the University of California Santa Cruz Institutional Review Board (protocol #HS-FY2023-193). Informed consent was obtained from all survey respondents prior to participation; respondents reviewed and signed a written consent form (available in English, Spanish, and Chinese simplified) describing the study's purpose, procedures, risks, benefits, voluntary nature, and confidentiality protections before beginning the survey.

Note that full information on the approval of the study protocol must also be provided in the manuscript.

Field-specific reporting

Please select the one below that is the best fit for your research. If you are not sure, read the appropriate sections before making your selection.

☐ Life sciences ☒ Behavioural & social sciences ☐ Ecological, evolutionary & environmental sciences

For a reference copy of the document with all sections, see nature.com/documents/nr-reporting-summary-flat.pdf

Behavioural & social sciences study design

All studies must disclose on these points even when the disclosure is negative.

Study description	Cross-sectional, community-engaged survey study (n = 1,691 California residents). Data are predominantly quantitative (5-point Likert-scale responses and demographic/geographic variables) with qualitative inputs from community-partner focus groups used to inform survey design and to help contextualize and interpret research findings but not entering the analyses reported here. All reported analyses are quantitative.
Research sample	The research sample comprised n = 1,691 adult California residents who completed the survey and met quality control criteria (see Data exclusions). Recruitment was concentrated in three coastal regions selected to capture geographic and demographic diversity along the California coast: Southeast San Francisco, the greater Monterey Bay, and Ventura/Oxnard counties. Demographic composition of the final analytical sample: 40.3% identified as White, 29.1% as Hispanic or Latino, 20.3% as Asian, 7.75% as Black or African American, 3.7% as American Indian or Alaskan Native, and 3.1% as Native Hawaiian or Pacific Islander. The sample skewed younger, with 51.1% under 40 years of age. Household income distribution was weighted toward lower brackets: 30.1% reported annual earnings below $59,999 and 27% reported $60,000–$119,999. Gender distribution was approximately balanced: 50.0% male, 43.5% female, and 2.5% transgender or non-binary; the remaining 4.1% declined to provide gender information. The sample's racial and ethnic composition is broadly representative of California's general population (per 2020 US Census data), with the notable intentional over-representation of American Indian/Alaskan Native and Native Hawaiian/Pacific Islander respondents (3.7% and 3.1% of the sample versus 0.4% each of California census respondents), reflecting the study's purposive sampling design. In-person recruitment explicitly targeted communities designated as disadvantaged or severely disadvantaged by the California Environmental Protection Agency's CalEnviroScreen tool to amplify voices typically absent from coastal-policy stakeholder engagement, and the survey was offered in English, Spanish, and Chinese (simplified) to broaden participation. This sampling design directly supports the manuscript's central research question of identifying disparities in coastal access and engagement that conventional probability samples typically under-represent.
Sampling strategy	The study employed purposive (non-probability) sampling explicitly designed to engage communities historically underrepresented in ocean and coastal management, with in-person recruitment targeting CalEnviroScreen-designated disadvantaged and severely disadvantaged communities across three focal coastal regions. The survey was offered in English, Spanish, and Chinese (simplified). No formal sample-size calculation was performed; recruitment continued until project resources were exhausted. The resulting analytical sample (n = 1,691) sufficient for the correlation, ANOVA, and OLS regression analyses reported here and supports stratified comparisons across demographic and geographic subgroups.
Data collection	The study utilized a mixed-mode survey administration approach. In-Person participants (n=1343) were recruited via tabling at community events or intercepted at coastal access points, laundromats, parks, and other public spaces. They were compensated with $20. Online (n=848): Participants were recruited through fliers, social media posts, and email lists. They were entered into a lottery prize drawing. In-Person: Data were recorded using paper or tablet. Online: Data were recorded using the respondent's personal computer or cellphone. The survey was administered in collective settings (though discussion between respondents was limited by procedure) by the research team in partnership with staff from five local non-profit organizations (who were typically present but not actively engaged during individual administrations). Blinding is not applicable; this is an observational, cross-sectional survey study with no experimental conditions, and the standardized instrument was applied identically to all respondents
Timing	Survey administration took place between July of 2024 and February of 2025.
Data exclusions	From an initial total of 2,191 surveys, the final sample of 1,691 was determined by excluding surveys that were less than 60% complete, completed in less than 400 seconds, associated with non-residents, or failed a response variability threshold (indicating a lack of thoughtful engagement as quantified statistically through a procedure described in the Methods text.).
Non-participation	Systematic refusal-rate tracking was not feasible for intercept-based recruitment in which respondents were approached opportunistically at community events and public spaces; declined participation and reasons for non-completion were not enumerated. The closest analog to dropout was n = 262 surveys removed for less than 60% completion.
Randomization	Participants were not allocated into experimental groups. The study utilized a cross-sectional survey design to analyze existing conditions rather than an experimental intervention. Since random allocation was not applicable, the authors controlled for covariates and confounding variables statistically during the analysis.

Reporting for specific materials, systems and methods

We require information from authors about some types of materials, experimental systems and methods used in many studies. Here, indicate whether each material, system or method listed is relevant to your study. If you are not sure if a list item applies to your research, read the appropriate section before selecting a response.

Materials & experimental systems

n/a	Involved in the study
☒	☐ Antibodies
☒	☐ Eukaryotic cell lines
☒	☐ Palaeontology and archaeology
☒	☐ Animals and other organisms
☒	☐ Clinical data
☒	☐ Dual use research of concern
☒	☐ Plants

Methods

n/a	Involved in the study
☒	☐ ChIP-seq
☒	☐ Flow cytometry
☒	☐ MRI-based neuroimaging

Plants

Seed stocks

Report on the source of all seed stocks or other plant material used. If applicable, state the seed stock centre and catalogue number. If plant specimens were collected from the field, describe the collection location, date and sampling procedures.

Novel plant genotypes

Describe the methods by which all novel plant genotypes were produced. This includes those generated by transgenic approaches, gene editing, chemical/radiation-based mutagenesis and hybridization. For transgenic lines, describe the transformation method, the number of independent lines analyzed and the generation upon which experiments were performed. For gene-edited lines, describe the editor used, the endogenous sequence targeted for editing, the targeting guide RNA sequence (if applicable) and how the editor was applied.

Authentication

Describe any authentication procedures for each seed stock used or novel genotype generated. Describe any experiments used to assess the effect of a mutation and, where applicable, how potential secondary effects (e.g. second site T-DNA insertions, mosiacism, off-target gene editing) were examined.
