# Supplementary material for: Racial and economic disparities in coastal access and engagement mediate the ocean’s contribution to human wellbeing
Source: Nat Commun. 2026 Jul 13;17:5975. doi: 10.1038/s41467-026-75034-4 (PMC13365518; doi:10.1038/s41467-026-75034-4)
Supplement: Supplementary file 3 — Transparent Peer Review file [file 41467_2026_75034_MOESM3_ESM.pdf]

nature portfolio

Peer Review File

Racial and Economic Disparities in Coastal Access and Engagement Mediate the Ocean's Contribution to Human Wellbeing

Corresponding Author: Dr Timothy Frawley

This file contains all reviewer reports in order by version, followed by all author rebuttals in order by version.

Version 0:

Reviewer comments:

Reviewer #1

(Remarks to the Author)
Thank you for the opportunity to review the paper "Racial and Economic Disparities in Coastal Access and Engagement Mediate the Ocean's Contribution to Human Wellbeing", which provides an important review of people in California's access to the ocean and its benefits.

Overall, I think the paper reads well and the results are significant for publication. I also think the paper needs some significant revisions before it is ready to be published, which I have further outlined below.

Firstly, the paper seems to make some significant statements that should be better underpinned by references, and/or explanations. Whilst some statements, such as that "California is often considered a world leader in protecting and managing ocean environments" would need further support, other statements, such as "knowledge gaps" in "who is able to access and benefit from ocean ecosystems" and who "have a voice in associated decision-making processes" - seems to be contextual or only grounded in one field of the literature, as this is well-established in the broader marine social sciences (i.e., Saunders et al. 2021 https://doi.org/10.3390/su12062560; Okafor-Yarwood et al. 2020 https://doi.org/10.3389/fmars.2020.00586; Bennett et al. 2021 https://doi.org/10.3389/fmars.2021.711538; Strand et al. 2024 https://doi.org/10.69902/8f1075e8; Lau et al. 2021 https://doi.org/10.1016/j.gloenvcha.2020.102208; Vierros et al. 2020 https://doi.org/10.1016/j.marpol.2020.104039; Rivers et al. 2023 https://doi.org/10.3389/fmars.2022.1084674; Bennett 2022 https://doi.org/10.3389/fmars.2022.873572; Crosman et al. 2022 https://doi.org/10.1038/s44183-022-00001-7; Flannery et al. 2018 https://doi.org/10.1016/j.marpol.2017.11.001)

Following from this, I would argue that there is in fact a lot of studies and literature on the social and cultural benefits to human relationships with the ocean and how this is for many interchangeable with cultural and social identities, spirituality, Indigenous knowledge systems, mental health (Lobo and Parsons https://doi.org/10.1177/27539687231179231, Poe et al. https://doi.org/10.1111/conl.12068, Gee et al., https://doi.org/10.1016/j.ocecoaman.2016.11.026, Fleming et al. https://doi.org/10.69902/8b06940f, Hau'ofa "We are the ocean", Ingersoll "Waves of knowing: a seascape epistemology", Strand et al. https://doi.org/10.3389/fmars.2022.886632; https://doi.org/10.1163/15718182-3104000, McKinley "socio-cultural dimensions of marine spatial planning", to name a few). In your statement that "we demonstrate, such benefits are not broadly understood" you should specify where and by whom. Several authors and people have expanded on this for a long time (i.e., Ingersoll, Hau'ofa).

This is also the case when it comes to the statement about environmental justice (415-419). Please specify whether you are referring to specific US policies or approaches here? I would argue that most environmental justice literature and frameworks specifically consider socio-cultural aspects of human wellbeing. Some concepts, such as "ocean justice" and "equity-centred approaches" should be defined or elaborated on when first mentioned, as there are several varying conceptualisations of this.

The paper would benefit from further information about the authors and their motivations for doing this study - preferably in the Introduction section.

I am missing a section discussing the implications of the results on ocean management and policies - and also how this relates back to theories of ocean equity, environmental justice, access and human wellbeing. How you are applying these theories should be further expanded on in the theoretical framework, and then brought back when you discuss your important findings.

Context matters. The introduction of “historical legacies of discrimination and dispossession" shaping ocean access, as well as the information introduced in 362-368 such as the forced displacement of Black coastal communities, should be introduced up front in the paper, and not only in the discussion. Please expand on how these structural barriers were (or were not) informing this study. Furthermore, at the end of your Discussion, where you argue that “coastal adaptation planning can be used as a lever to redress historical inequalities”, please expand on how and talk to the aspects only outlined in Table 3 at this stage. It would be great to see some specific recommendations following from your review in the main text and study and have a brief discussion attached to each.

The results and methods would also benefit from further analysis of the limitations of the research approach, for example expanding on what you did to better access inland areas and how histories of gentrification/increase in prices, forced removals, barriers to ocean access would further impact people’s access to live by the ocean and how this would have impacted your study “sample”. It would also be good to know why the specific survey prompts were used, and who these related to the theoretical framework.

Line-by-line comments

68-69 - include reference here

72 - relationship with “and access to”?

78-81 - good!

94 - it would be good to elaborate a bit more on the California Coastal Act for people who are not familiar - and also what the “recent progress” refers to.

91-99 - great!

119 - “modern development scholars” I am unsure these authors would characterise themselves as such - please explain what is meant here and why this is used. As mentioned above, I also think the social benefits are not necessarily poorly studied, although they are often overlooked and neglected.

176 - please expand a bit on why you think it is that individuals tavelling longer distances were likely to reside in more vulnerable communities. In the introduction it would be great with more detail on the context and who you reached (and perhaps did not reach) with this survey.

200 - the strongest correlation with frequency with people identifying as White - it would be good to hear more about what this means in the analysis - and the socio-economic and/or historical factors that might have impacted this

Table 1 - prompt 5. “I possess the required skills or knowledge” - firstly, how was this elaborated and conceptualised? It would be good to see how this was explained to respondents. Secondly, what about being able to contribute your own knowledge to ocean activities and/or decision-making, and feeling heard? It would be good to include a short paragraph on why these eight prompts were chosen.

246-248 - it might be good with a footnote briefly explaining ANOVA, post-hoc Turkey HSD and OLS

304-306 - again, it would be good to know up front a bit more about the demographics and historical context determining who might live near or far away from the coast

323 - perhaps in one sentence restate how community vulnerability was conceptualised here

356 - historical legacies of discrimination and dispossession - this should be considered in the beginning of the paper

362-368 - same as above, should be mentioned in the Introduction when introducing the specific context in which you are working in

370 - “may be” - I think it is, no?

374 - “may limit” - it is quite clear that it does?

377 - gender dimensions receiving less research attention - please be specific - less than what? Where? By whom?

378 - benefitting from ecosystems - but also ocean specifically?

382 - Again, the act should be further introduced when first mentioned

386 - please explain what is meant by recognitional justice here

402 - expanding body of literature - also in the marine social sciences, ocean governance +++ literature

407-408 - I do not think you are demonstrating that “such benefits are not broadly understood” - you seem to be overlooking a very broad set of literature and scholarship. See Ingersoll or other authors mentioned above.

411 - specify that this is in California. Important to reflect on where you did your survey and what it tells you. There is definitely lessons to be drawn more broadly, and it would be great if you could bring in some literature looking at similar aspects in various contexts.

415-419 - I would argue a lot of environmental justice approaches go beyond physical infrastructure and/or economic opportunity. Please provide references, and consider looking a bit deeper into the literature.
422-423 - California considered a world leader - According to whom and what metrics?

Table 3 - Please include these in the main text and speak to them in more detail. Has this been implemented to some extent already, and in that case by whom? Who should be doing this, and where should the budget come from? What are actions or recommendations at different levels (personal, community, city) or tiers of government?

434 - the survey results did not seem to speak much to women and non-binary people - can you expand here if they did?

435 - how do you define ocean justice?

442 - what do you mean by equity-centered approaches?

446 - could you be more specific? How can and should coastal management and policies redress racial disparities when it comes to ocean access and benefits?

454 - what might be a limitation of intercepting at coastal access points? Just thinking of access…

464-470 - it would be great to mention this up front in the paper

(Remarks on code availability)

Reviewer #2

(Remarks to the Author)
This paper examines the factors that mediate coastal access and the ocean's contributions to wellbeing with a focus on California. It is a well conceptualized study and well written paper that presents novel empirical results. The topic is important and will be of interest to a broad audience of sustainability and ocean professionals and researchers. Overall, I would recommend that this paper be accepted subject to minor revisions. Below are suggestions to improve the paper, noting that none of them require new analysis - just some additional content, restructuring of paragraphs, providing headers, potential visualization of results, and concluding with a strong focus on access and wellbeing.

Good introduction.

Section 2.0 - Theoretical Framework - at the end of the first paragraph it says that it draws on theories of ocean equity, access and environmental justice, and human wellbeing. Each of these needs to be unpacked a little more - explain a bit about each topic and how the study/framework links to them. Especially, ocean equity - which is a related topic, but I don't really see how this is integrated into the framework as it is not in the Supplemental Table 1. Also, regarding the framework - I can't see how it links to your survey methods - so would suggest to provide a table in the main manuscript that shows a) each main topic, b) the sub-attributes of that main topic that are covered in the survey, c) the survey question related to that attribute, and d) the scale used. Similar tables are provided in Hicks et al 2014 (Social, institutional, and knowledge mechanisms mediate diverse ecosystem service benefits from coral reefs) and Bennett et al 2020 (Social equity and marine protected areas: Perceptions of small-scale fishermen in the Mediterranean Sea). This provides a great quick reference.

For the results, it could really use sub-headers for each section within the results - to clearly separate the topics and make it easier for the reader to follow.
- Small edit, line 308-309 - "...was associated with significantly lower wellbeing..." should be "was associated with significant lower contributions to wellbeing..." (as the topic is "contributions to wellbeing", not just "wellbeing"). But, consider this elsewhere in the way the results are written - it is not wellbeing, but the level to which the ocean is contributing to wellbeing.
- Figure 4 - Is the result for 'Distance' missing from Figure 4a at top left?

For a couple of the results tables in the paper, I was wondering whether there was a better way to visualize them. For

consideration. owever, I do understand that a combination of figures and tables is good.

There are some long paragraphs throughout the results and elsewhere in the paper that are too long, and hard to follow because there is not clear topic sentence. Suggest to go through and break these up and give them more structure.

For the discussion, most of it is good - but it is really weird to have the last paragraph suddenly being about "ocean justice" in policy and practice. Why introduce this new jargon, it does not help - and instead confuses this reader. I would keep this focused on the central topic of this paper - ACCESS. So, I would suggest to reframe this section to be about "Advancing ocean access and human wellbeing through policy and practice" - and really dig in on the path to increase the ocean's contributions to the wellbeing of all citizens through a focus on access for all people. It has been very focused - finish strong!

(Remarks on code availability)
I am not qualified to review the code for this paper.

Reviewer #3

(Remarks to the Author)
Date: October 24, 2025
Review of: “Racial and Economic Disparities in Coastal Access and Engagement Mediate the Ocean’s Contribution to Human Wellbeing”
For: Nature Communications

Recommendation: Accept with minor revisions

General Comments:

Thanks for the opportunity to review “Racial and Economic Disparities in Coastal Access and Engagement Mediate the Ocean’s Contribution to Human Wellbeing”. This paper investigates equity aspects of ocean access in parts of California, using a definition of that term that expands beyond spatial/physical dimensions. The authors furthermore underpin their investigation with a framework for exploring barriers to accessibility, and their implications for human wellbeing.

I think this is a valuable contribution, providing a rich source of data that allows the authors to pick apart a wide variety of complex factors associated with ocean access and well-being. The conclusions reinforce and embellish a variety of observations about the structural factors leading to inequitable outcomes for low-income households and vulnerable communities. As elaborated below, I think the authors should do more to explain the specific implications of their analysis, both for the broader literature, and for policy makers.

A note about my own expertise. While I was largely able to follow the analytical steps outlined in this paper, I am not able to assess the soundness or validity of the statistical calculations reported in detail, and it is important that this be done by another reviewer.

I have a few high-level comments and questions, followed by some minor details that I noticed while reading.

Implications of the study. Overall, I think the authors could do more to explain how this study and analysis changes and/or advances our ability to actually address the systemic problems that they are reporting on. I appreciate that the paper lends analytical heft to existing criticisms of the material/economic framing of ocean benefits, but it would be great to see this unpacked in a couple of different ways:

1. Rather than just reporting how these results are consistent with other literature, the authors should explain what specific gaps or shortcomings in previous literature are addressed by this study’s results.

2. The policy recommendations in Table 3 are very straightforward, and would have seemed quite sensible and important if put forward on their own (i.e., without this study backing them up). Are there any new/different policy recommendations that this analysis points to, which might not have been on the table otherwise?

Finally, I’d like the authors to address the relevance of these results beyond California. Specifically, is there anything unique about California that would suggest caution in extending these results to other areas, or even extending this very same analytical approach? The importance of this question is perhaps reinforced by the major differences found between southern and northern locations.

Specific Comments/Edits

Line 199 -- Typo in “Norther”
Line 215 -- “Choose” should be “chose”
Line 296 -- Clarify what you mean by “which are the focus of most management and policy efforts”

Line 464 -- Missing word: in-depth “in” the context of…

(Remarks on code availability)

Reviewer #4

(Remarks to the Author)
I co-reviewed this manuscript with one of the reviewers who provided the listed reports. This is part of the Nature Communications initiative to facilitate training in peer review and to provide appropriate recognition for Early Career Researchers who co-review manuscripts.

(Remarks on code availability)

Reviewer #5

(Remarks to the Author)
The paper addresses a significant and important area of research and contributes novel findings. The work can feed into global debates on ocean access and wellbeing and provides important contributions to discussions of barriers to ocean use, having practical implications and potential for impacts to address inequalities. We congratulate the authors on identifying a key area for further research and using a large and diverse sample which pays attention to representation. The paper is generally well written and easy to follow.

The paper starts with a clear introduction to the knowledge gaps that exist on barriers that mediate access and benefits from ocean ecosystems and the need for further research in this area. Research to date has typically focused on the barriers to access and benefits related to green space and the intersectionality related to this, and so it is novel to see this research focused on oceans, which is particularly important as it has been demonstrated that access to oceans and seas can have significant benefits for wellbeing. Whilst the introduction is well written it would be good to acknowledge some of the research that has been done on green space, to highlight that access and barriers to environmental spaces are not felt evenly across publics and whilst there is increasing recognition of this for green space there has been little/no attention to this for oceans/seas. Furthermore, terms such as structural barriers, compounding, cascading, and justice/climate justice, environmental justice, recognitional justice, ocean justice - would benefit from clearer definitions, especially if they play a central role in the argument

The paper uses the state of California, USA as a case study. California is a state known for biodiversity, cultural significance and a leader in the protection and management of ocean environments, and so represents a very appropriate case study. The study administered 1691 survey to learn about the factors mediating ocean use and benefits with a focus on wellbeing. The work has paid attention to include typically underrepresented demographics, as would be expected from a study with these objectives, however we recognise the difficulties in doing this in practice and the authors should be congratulated for this. Those who identified as Hispanic or Latino seem slightly less represented than the California population (29.1% of the survey compared to compared to 39.4% within the population) and whilst I acknowledge the difficulties of ensuring proportional representation the difference should be acknowledged and discussed within the paper. There is no discussion of limitations of the work within the paper and this should be addressed.

The paper develops a framework to conceptualise the barriers to ocean access and the ocean’s contributions to human wellbeing. This, and how it relates to the survey instrument developed, is an important contribution, as this can be adapted for use in other regions and the significance and potential for transferability could be highlighted more within the paper, i.e. the results are of use for all, not just to California, and more discussion of potential implications outside of California would be good. Expanding the discussion on theories of ocean equity, access, environmental justice, and human wellbeing used in the framework (either in the theoretical framework section or the supplementary info if the word count does not allow) and moving figure 1 to within theoretical framework section would improve the paper.

The results of the survey are well analysed and generally clearly presented, demonstrating how geographic, gender, race, and socioeconomic predictors intersect to influence ocean access and contributed to human wellbeing which is segmented into material, subjective, and relational wellbeing. Figure 3, is well presented. However, is it striking that female has been removed (why not male!), I understand that that is because “variables contained redundant information”. Therefore, it would be more accurate to represent this as binary (male/female) and trans/non-binary, it would also save a redundant line in the figure. This description is also necessary for other figures when female has been omitted e.g. Fig 5 and Supplemental Figure 4. In Fig 3 Native Hawaiian or pacific islander has no results shown, is this because the sample is too small? If so this should be discussed and/or perhaps mention in the description.

Table 1 is very important for the paper’s findings and needs careful explanation. The paper states that knowledge factors are the “most influential in mediating differences in the overall barriers score”. However, the figure description states that “smaller mean values are associated with greater reported barriers”, yet physical environment barriers have a smaller mean (3.81 compared to 3.89). This needs checking or the information used for interpreting the figure clarified. The sentence

“mean item total correlation coefficients suggest that knowledge factors (0.7 ± 0.01), were most influential in mediating differences in the overall barriers score” is also confusing because it uses 0.7 ± 0.01 and my understanding is that this figure is only for prompt 8, if this is correct is should be explicitly stated, or the correct figure used. It would also be useful to highlight in the text the prompts which are contributing most significantly to the results as these are then referred to in the discussion. Similarly, for table 2.

More careful attention needs to be paid to the discussion and how this relates to the results presented. The opening line states “Our findings reveal how racial identity, socioeconomic status, gender, and geography interact to shape ocean access and wellbeing”. However, gender is not found to be significant in the results. Again it is stated “Although the gendered dimensions of ocean access and wellbeing have received less research attention, our results support the assertion that gender is a critical determinant of how people differentially benefit from ecosystems”, I should imagine gender would be an important determinant but I do not see this reflected in the results that are presented and if this is a key finding the results need to highlight this. Additionally, it is stated that “More recently, restrictive housing covenants and lending practices32, uneven investment in community infrastructure and environmental education33, and accelerating coastal gentrification1,34 continue to shape who feels safe, connected to, and welcome in ocean areas across the state”. The safe and connected part of this sentence needs a reference, particularly as the results presented here (table 1 and post-hoc Tukey HSD) show that social barriers were not significant. I don’t doubt that there has not been attention paid to social justice issues in housing practices etc. but more careful phrasing is needed to ensure distinction between suggestion for causes and clear results from the study.

You also state that significant barriers reported by Black and Asian American respondents, [..] are the result of structural processes embedded in racialized histories” – while this is highly likely to be true it is important to show how that was found based on the results, or to present this as a likely cause for the results you found. Greater engagement with existing literature on intersectionality in environmental studies could help here.

I enjoyed your discussion on overlooked benefits of relational and subjective ocean wellbeing. However, on line 411 it states, “By impeding ocean access”, it may be that there is not an intentional impediment but more because of a lack of consideration. I think this should also be discussed because typically environment and social goals have been considered in isolation and results, such as yours, demonstrate that unless understood as intersecting then action for admirable environmental goals may have adverse and unequal social implications. A short discussion of how your results demonstrate the need to consider environmental and social aspects together, especially in coastal areas that will need to adapt to rising sea levels and changing climates, would demonstrate more clearly the significance of your work outside of California.

Your concluding sentence “Climate change presents not only an existential threat for vulnerable coastal populations, but also a rare opportunity for transformative change. With intentional design, coastal adaptation planning can be used as a lever to redress (rather than reproduce or exacerbate) historical inequalities51 and help build a future based upon universal ocean access, belonging, and wellbeing” is excellent and I applaud you on the research you are doing to contribute to this.

In summary this paper addresses an important and novel area of research with a large data set of results. However, there are a few things to address before the paper is suitable for publication.

Specifically:

Line 1: Consider editing the title e.g. Uncovering racial and economic disparities in ocean access and contribution to wellbeing -this would highlight the transferability of the approach to other areas

Re-frame the introduction around the global significance of the work, i.e. need for greater research and investigation of coastal barrier as an environmental justice issue in general, then state the use California as a case study, i.e. the novelty and importance that these issues are researched and that California is a case study that will be used to do this but results are relevant for elsewhere to, e.g. the framework and survey instrument

Acknowledge past work on intersectionality even if this is only in alternative environments i.e. green space

Expanding the discussion on theories shaping the theoretical framework and demonstrate how this relates to the survey instrument

Fig 3, 5 and Supplemental Figure 4 change male to binary (female/male)

Check the description and interpretations used in table 1. Consider highlighting the most significant prompts in table 1 and 2

Improve the connection between the Results and Discussion sections to ensure clarity between key and novel findings and suggested causes for these to help guide the reader through the interpretation of the findings and highlight the contribution made by this work

Add discussion of limitations of the work and ideas for further work, e.g. other contexts, research to advance overcoming barriers etc.

A reminder that all error bars should be defined in the corresponding figure legends and should include a description of any error bars.

Minor comments:

Line 72: Add supporting reference to the following statement "Geography, gender, class, and race - and the interactions among them - all function to constrain or enhance people's relationship with the ocean".

Line 72-73: "Though recent research has advanced scientific understanding of the links between marine governance and environmental sustainability3" the link between this and the identified gap could be made more explicit.

Line 79: Suggest adding what are the major coastal management frameworks that prioritise ecological metrics over social indicators.

Line 99- consider adding, or introduce new inequalities

Line 102: Consider adding definition of key terminologies, in particular, ocean access and wellbeing, compounding, cascading, justice (climate justice, environmental justice, recognitional justice, ocean justice)

Line 146: Figure 1, given that the text refers to Figure 1 in the Theoretical Framework section and not mentioned in the Results section consider moving it

Lines 158: Figure 2, add scales to the maps

Lines 160-161: Figure2, move letter (A) "across California at-large (A) (remove here) and the three focal regions where administration efforts were concentrated: (A) (add here) the Greater Monterey Bay (B) […]

Line 199: typo NortherN region, missing N

229 physical environment repeated twice?

Line 246: typo subS-scales, remove S

Line 233: Table 1, it would benefit from a better explanation of the composite scale and how multiple dimensions were created.

Line 247: Tukey's Honestly Significant Difference (HSD) – could be spelled out since is the first time being mentioned

Line 313: Figure 5, where can we find the regression tables?

Line 361: in the statement "Significant barriers reported by Black and Asian American respondents, [..] are the result of structural processes embedded in racialized histories" – while one may agree please ensure careful phrasing to ensure distinction between suggestion for causes and clear results from the study.

Line 352: While one may infer the meaning of structural barriers and you provide examples a definition could guide the reader

Line 398: "previous literature suggests that […] subsistence goals" please provide references of previous studies.

Lines 718-719: Supplemental Table 1. It would be helpful to have the key references in relation to each definition and subcategory rather than "barriers to ocean" as a whole

Lines 718-719: Supplemental Table 1, unless in the authors to guidelines, move table captions to the top of the table

Lines 722-723: Supplemental Table 2, unless in the authors to guidelines, move table caption to the top of the table

Lines 731-732: order of bar plots is not mirroring what is in the caption. A) racial-ethic categories, B) annual household income, C) frequency of use of ocean and coastal areas as opposed to "A) annual household income, B) frequency of use of ocean and coastal areas, and C) racial-ethnic categories."

(Remarks on code availability)

Reviewer #6

(Remarks to the Author)
I co-reviewed this manuscript with one of the reviewers who provided the listed reports. This is part of the Nature Communications initiative to facilitate training in peer review and to provide appropriate recognition for Early Career Researchers who co-review manuscripts.

(Remarks on code availability)

Version 1:

Reviewer comments:

Reviewer #2

(Remarks to the Author)
Overall, the authors have done a fantastic job addressing all of the reviewer comments including those from me. I recommend it be accepted subject to a few minor revision below.

A couple of minor points that I think should still be addressed:

1) Ocean equity - While I do see the topic of access and human wellbeing related to ocean equity (and many of the other theories mentioned in the paper), I think these topics can be briefly introduced and the link made in the introductory text to situate the topic. But, it seems like a stretch to include "distributional ocean equity" in the theoretical framework for the paper (i.e., figure 1) as this topic is not really in the survey. The survey is about ocean access and human wellbeing. I would suggest to remove "distributional ocean equity" from the figure, it is a distraction from the main topics of the paper (i.e., access and wellbeing) which stand on their own.

2) Survey items - I appreciated that the authors have put the general survey items for each of ocean access and human wellbeing in the Supplemenatary materials. However, it would still be good to include a short introduction to the general survey items in the body of the text - alongside an introduction/references to the theory that they are meant to support. (E.g., Drawing on human well-being theory (ref, ref, ref), we developed survey indictors for .... the following aspects of human wellbeing ... which we asked participants to score as follows... Similarly, for access, we created indicators related to the X, Y, Z, mechanisms that mediate access...etc.) Or, you might use an abbreviated table to bring readers up to speed. Without introducing this, it is hard for the reader to follow the methods and theoretical framework that guides the analysis.

(Remarks on code availability)

Reviewer #3

(Remarks to the Author)
I appreciate the authors' thorough consideration and responses to the reviewer comments. For my part, the points I raised in my initial review have been addressed, and I have no remaining concerns about the manuscript.

(Remarks on code availability)

Reviewer #5

(Remarks to the Author)
Thank you to the authors for their careful and thorough consideration of our comments on the manuscript, I can see from the response document and the manuscript that these have been very well taken into consideration.

I particularly appreciate the effort taken with the introduction and theoretical framework to strengthen and contextualise the contribution, as well as the clarification of definitions that have been added.

I thank the authors for the clarifications on the presentation of the gender variables in the figures and appreciate the effort taken to provide more information for those readers wishing to consider the analysis through the lens of gender.

I also feel that the reformatting of table 1 as a figure has improved the presentation of the results.

There has been some softening of the language concerning causality between results and likely causes and the additional discussion of these is now excellent. The introduction of the Policy implictions of the work and the Study Limitations and Next Steps for Research section is also welcomed. I was very leased to see the introduction of a paragraph on the motivation for the study, although I think this would be better placed in the introduction, so that it can provide context for the reviewer

Overall, the manuscript has been improved by the responses to the reviewer comments and I only have a few remaining minor things to consider before publication. First, the mention of Jim Crow South may not make sense to those not familiar with US history, as Nature Communications has an international audience this should be re-written to explain e.g. systemic racial segregation enforced in the south of the country. Second, could a reference for California Environmental Protection Agency's CalEnviroScreen index be added? Finally, consider moving the motivation statement to the introduction

(Remarks on code availability)
All code is comprehensively provided

Open Access This Peer Review File is licensed under a Creative Commons Attribution 4.0 International License, which permits use, sharing, adaptation, distribution and reproduction in any medium or format, as long as you give appropriate credit to the original author(s) and the source, provide a link to the Creative Commons license, and indicate if changes were made.
In cases where reviewers are anonymous, credit should be given to 'Anonymous Referee' and the source.
The images or other third party material in this Peer Review File are included in the article's Creative Commons license, unless indicated otherwise in a credit line to the material. If material is not included in the article's Creative Commons license and your intended use is not permitted by statutory regulation or exceeds the permitted use, you will need to obtain permission directly from the copyright holder.
To view a copy of this license, visit https://creativecommons.org/licenses/by/4.0/

Dear Reviewers,

We very much appreciate the time and effort spent reviewing our manuscript and are grateful for the opportunity to improve it. We believe that our efforts to address the fair and insightful critiques of all six Reviewers have strengthened the manuscript significantly. While more detailed replies can be found below in response to individual comments, the focus of our efforts in undertaking this revision can be summarized as follows:

1) Substantial revisions to the Introduction, Theoretical Framework, and Discussion text to better engage with existing literature regarding ocean equity; the social and cultural benefits of human relationships with the ocean; and intersectional environmental studies concerning green space and terrestrial conservation. This text provides the explicit definitions requested by the Reviewers while trying to better describe the linkages between core concepts (i.e., access, wellbeing, equity, and environmental justice), the specific research gap the study is designed to address, and the historical context in which it is grounded.

2) Restructuring the format and presentation of key figures and results text to a) better highlight the study's topline findings (i.e., the identification of what dimensional sub-scales and survey prompts were most effective at measuring the core constructs of the composite barriers and wellbeing scales); b) present the analysis of gender variables and data in a more nuanced and comprehensive fashion.

3) Expanding and restructuring the Discussion text to a) address the limitations of the study and propose next steps for research; b) unpack the management recommendations provided in the final table and better ground them in specific, empirical results; and c) expand upon the implications of our analysis for study systems beyond California.

Reviewer #1

Thank you for the opportunity to review the paper "Racial and Economic Disparities in Coastal Access and Engagement Mediate the Ocean's Contribution to Human Wellbeing", which provides an important review of people in California's access to the ocean and its benefits.

Overall, I think the paper reads well and the results are significant for publication. I also think the paper needs some significant revisions before it is ready to be published, which I have further outlined below.

COMMENT: Firstly, the paper seems to make some significant statements that should be better underpinned by references, and/or explanations. Whilst some statements, such as that "California is often considered a world leader in protecting and managing ocean environments" would need further support, other statements, such as "knowledge gaps" in "who is able to access and benefit from ocean ecosystems" and who "have a voice in associated decision-making processes" - seems to be contextual or only grounded in one field of the literature, as this is well-established

in the broader marine social sciences (i.e., Saunders et al. 2021 https://doi.org/10.3390/su12062560; Okafor-Yarwood et al. 2020 https://doi.org/10.3389/fmars.2020.00586; Bennett et al. 2021 https://doi.org/10.3389/fmars.2021.711538; Strand et al. 2024 https://doi.org/10.69902/8f1075e8; Lau et al. 2021 https://doi.org/10.1016/j.gloenvcha.2020.102208; Vierros et al. 2020 https://doi.org/10.1016/j.marpol.2020.104039; Rivers et al. 2023 https://doi.org/10.3389/fmars.2022.1084674; Bennett 2022 https://doi.org/10.3389/fmars.2022.873572; Crosman et al. 2022 https://doi.org/10.1038/s44183-022-00001-7; Flannery et al. 2018 https://doi.org/10.1016/j.marpol.2017.11.001)

Following from this, I would argue that there is in fact a lot of studies and literature on the social and cultural benefits to human relationships with the ocean and how this is for many interchangeable with cultural and social identities, spirituality, Indigenous knowledge systems, mental health (Lobo and Parsons https://doi.org/10.1177/27539687231179231, Poe et al. https://doi.org/10.1111/conl.12068, Gee et al., https://doi.org/10.1016/j.ocecoaman.2016.11.026, Fleming et al. https://doi.org/10.69902/8b06940f, Hau'ofa "We are the ocean", Ingersoll "Waves of knowing: a seascape epistemology", Strand et al. https://doi.org/10.3389/fmars.2022.886632; https://doi.org/10.1163/15718182-3104000, McKinley "socio-cultural dimensions of marine spatial planning", to name a few). In your statement that "we demonstrate, such benefits are not broadly understood" you should specify where and by whom. Several authors and people have expanded on this for a long time (i.e., Ingersoll, Hau'ofa).

RESPONSE: We are thankful for the reviewer's insightful and constructive critique of the lens through which the study was previously framed and are grateful for the effort they have made in providing references to facilitate our efforts to address it. We agree that the previous version of the manuscript did not accurately a) reflect the existing literature concerning the social and cultural benefits of human relationships with the ocean (particularly that rooted in indigenous, black, brown, and (global) southern intellectual traditions); b) clearly articulate the specific research gap that the study was designed to address. In response, we have revised the first two paragraphs of the manuscript (i.e., Lines 19-43) so that they now read as follows:

Oceans and coasts sustain the livelihoods, wellbeing, and cultural heritage of billions of people worldwide. Yet, access to these environments remains deeply unequal, shaped by historical policies and cultural processes driving economic disenfranchisement and social exclusion (Reineman et al., 2016; Österblom et al., 2023). Geography, gender, class, and race - and the interactions among them - all function to constrain or enhance people's relationship with the ocean (Saunders et al., 2020; Crosman et al., 2022). Western governance and policy paradigms primarily frame the ocean as a biophysical resource (Costanza 1999, World Bank 2017); yet indigenous, black, brown, and (global) southern intellectual traditions have long recognized the ocean as a source of kinship, connection, identity, cosmology, and spirituality (Ingersoll, 2016; Lobo & Parsons, 2023; Teixidor-Toneu et al., 2025). Where management strategies conceptualize humans as separate from nature, whether prioritizing resource extraction for macroeconomic gain or exclusionary measures for conservation, they have often functioned to undermine local rights, relationships, and knowledge systems, and reinforce the historical legacy of colonialism (Okafor-Yarwood et al., 2020; Strand et al., 2024). Indeed,

mounting evidence suggests that where socio-cultural dimensions are not considered alongside ecological and economic metrics, many policy approaches may serve only to reinforce inequality and the institutional and legislative barriers perpetuating cycles of exclusion and environmental decline (Poe et al., 2014; Finkbeiner et al., 2017; Flannery et al., 2018; de Vos et al., 2023).

In recent years marine social scientists and commons scholars have advanced and refined theoretical frameworks describing the fundamental links between equity (defined broadly as the fair and just conditions and treatment of people), governance, and ocean sustainability (Crosman et al., 2022; Österblom et al., 2023; Bennett et al., 2025). Despite increasing recognition of the importance of equity considerations in the durability, legitimacy, and efficacy of coastal management, their assessment and operationalization remain an enduring challenge (Crosman et al., 2022). Empirical, place-based investigations concerning the structural factors mediating who is able to access and benefit from ocean ecosystems (and why) remain scarce, particularly across highly-developed, urbanized and/or multicultural coastlines characterized by complex and incompletely understood patterns of use and proximity.

COMMENT: This is also the case when it comes to the statement about environmental justice (415-419). Please specify whether you are referring to specific US policies or approaches here? I would argue that most environmental justice literature and frameworks specifically consider socio-cultural aspects of human wellbeing. Some concepts, such as “ocean justice” and “equity-centred approaches” should be defined or elaborated on when first mentioned, as there are several varying conceptualisations of this.

RESPONSE: In this revision we have expanded and rewritten the Theoretical Framework section to provide a) specific definitions of environmental justice, procedural equity, recognitional equity, distributional equity, access, wellbeing, and structural processes; b) better acknowledge the scholarly literature and traditions who helped develop and advance such concepts; and c) narrow our critique of existing management frameworks to those based in a specific, ideological orientation. Throughout the manuscript (see Line 24, Line 27, Lines 114, Line 441) we have tried to better target our critique of current management paradigms to those Western and Anglo-European approaches that are motivated by market-liberal logic. For more information please see more detailed responses to Reviewer 2 and Reviewer(s) 5 & 6.

The paper would benefit from further information about the authors and their motivations for doing this study - preferably in the Introduction section.

The following text has been inserted into Lines 699-706 of the revised manuscript to provide the motivation and background information requested by the reviewer: “*This research was conducted by a multi-disciplinary collective of students, academic researchers and community practitioners who acknowledge that their perspectives are shaped by diverse backgrounds in marine social science, ecology, anthropology, Native American and Indigenous Studies, and sustainability science. The team, motivated to conduct place-based investigations concerning equity in ocean access and benefits, initially coalesced around a research call (i.e., public funds administered through the California Drought, Water, Parks, Climate, Coastal Protection, and Outdoor Access for All Act of 2018) with resources specifically allocated to address the needs of underserved communities.*” While we agree with the reviewer’s comment and acknowledge the value and importance of positionality statements in contextualizing this

type of research, we ultimately feel the text is best suited for the Methods rather than the Introduction.

COMMENT: I am missing a section discussing the implications of the results on ocean management and policies - and also how this relates back to theories of ocean equity, environmental justice, access and human wellbeing. How you are applying these theories should be further expanded on in the theoretical framework, and then brought back when you discuss your important findings.

RESPONSE: In response to feedback provided by this Reviewer, and parallel requests made by Reviewer(s) 3&4 and Reviewer(s) 5 & 6, the final section of the Discussion has been expanded significantly in order to better ground the management recommendations in the empirical results, expand on their implications for policy within and beyond California, and better relating them back to theories of ocean equity, environmental justice, access, and human wellbeing. This text, on Lines 501-559 of the revised manuscript now reads:

*"To advance ocean equity, governments, funders, and practitioners must invest in creating, restoring, and/or sustaining meaningful connections to ocean spaces (**Table 3**). More specifically, our finding that knowledge represents one of the most consistent and influential barriers to ocean access suggests that targeted (and subsidized) Education and Outreach efforts should be prioritized. Compared to green space, the impact of knowledge on ocean access may be comparatively acute as many forms of engagement and participation require ocean literacy and water competence, a specific set of physical skills and safety knowledge often informally transmitted and unequally distributed across society (Wiltse 2014; Strand et al., 2023). While diverse forms of local ecological knowledge helped support resilient human-ocean relationships in California's not-too-distant past (McCovy et al., 1980; Erlandson et al., 2008), knowledge systems are subject to degradation when exposed to rapid and/or compounding process of social and environmental change (Frawley et al., 2019; Teixidor-Toneu et al., 2025). Efforts to build ocean literacy across underserved communities should be cognizant of such dynamics and seek to promote diverse, co-created and user accessible knowledge systems rather than reinforcing existing power dynamics by exclusively relying upon western language and science (Strand et al., 2022). Likewise, recruiting enforcement and management staff that reflect community demographics and providing cultural competency training through Workforce Development initiatives may help ensure that rules, regulations, and safety information are communicated effectively and inclusively.*

*Our findings suggest that the primary crisis of ocean equity may no longer be solely about subsistence and economic rights, but the fundamental human need to connect to, belong in, and access restorative blue spaces (Fleming et al., 2024). This aligns with the concept of 'realized access' which suggests that socio-personal dimensions of accessibility are as important as legal rights (Wang et al., 2015) and that cultural identity, social relationships and personal resources fundamentally mediate the ability to drive benefits from ocean resources (Seto et al., 2024). As compared to other regional research documenting barriers to urban greenspace access for minority and low-income populations (Wolch et al., 2005; Williams et al., 2020), fear of crime may not be a primary limiting factor in coastal access (i.e., most survey respondents reported feeling welcome in ocean and coastal spaces, though **Supplemental Figure 6** suggests this was somewhat less true for minority populations) yet personal barriers related to*

transportation access and geographic distance appeared comparably acute. Consequently, if the primary value of the coast for vulnerable communities lies in social cohesion, mental health, and spiritual renewal, then management bodies must invest in Coastal Infrastructure & Environmental Management which makes the use of local ocean areas for such purposes feasible, attractive, and affordable. In California specifically, agencies must prioritize more frequent and better integrated public transportation to pierce the coastal curtain and bridge the gap between isolated urban and/or urban communities and the coast. Where proximate shorelines are degraded or polluted, creative strategies may be required to remediate development, restore functional habitat, and transform neglected coastal fringes into community assets (Wolch et al., 2005).

In recent years many regional and national resource management agencies have made significant (if episodic) progress in advancing recognitional equity by acknowledging historical injustice and the plurality of knowledge and value systems relevant to ocean management. Yet, distributional outcomes are likely to remain elusive without corresponding interventions designed to enhance procedural equity via Funding & Grants and Participatory Governance. Equitable ocean outcomes require moving beyond consultation models that treat community input as a social license toward embracing efforts to redistribute resources and decentralize decision-making power (Crosman et al., 2022; Österblom et al., 2023). While the emerging focus on ocean equity is a positive development, environmental justice scholarship suggests that a sustainable and equitable future may ultimately require reckoning with the market-liberal logics and hierarchies through which bourgeois environmental organizations and the global ocean governance system derive their present authority (Cole & Foster, 2001; O'Neill et al., 2025). Though social and environmental goals have traditionally been pursued in isolation, integrated approaches can help address the root causes simultaneously fueling class exploitation, social inequality and environmental degradation (Frawley et al, 2019). In this respect, global environmental change presents not only an existential threat for vulnerable coastal populations, but also a rare opportunity for transformative change. With intentional design, coastal adaptation planning can be used as a lever to redress (rather than reproduce or exacerbate) historical inequalities (Hardy et al., 2017) and help build a future based upon universal ocean access, belonging, and wellbeing."

COMMENT: Context matters. The introduction of "historical legacies of discrimination and dispossession" shaping ocean access, as well as the information introduced in 362-368 such as the forced displacement of Black coastal communities, should be introduced up front in the paper, and not only in the discussion. Please expand on how these structural barriers were (or were not) informing this study.

RESPONSE: We take the reviewer's point that context matters and that more information is needed upfront regarding the socioeconomic processes and historical events that have shaped the demographics of coastal California over time. The following text has been added to Lines 44-59 of the Introduction where coastal California is first introduced as the study system:

Coastal California is uniquely suited to addressing this research gap. California's coastal and marine zones are of fundamental importance to the environmental and socioeconomic sustainability of the state, with coastal counties home to approximately 68% of the state's 38.9 million residents (Thorne et al., 2024). The region's diverse demographics have

been shaped over time by waves of migration, dispossession, and exclusion. During the Spanish, Mexican, and American colonial eras, indigenous societies rooted in sophisticated and reciprocal marine and terrestrial stewardship ethics were disrupted and forcibly displaced (Erlandson et al., 2008; Akins & Bauer 2021). The late 19th and early 20th centuries saw the arrival of Chinese, Japanese, Italian and Portuguese communities responsible for the development of commercial fisheries (Armentrout-Ma, 1981; McCovy 1986), followed by the Second Great Migration of the 1940s where booming wartime shipyards drew thousands of African American families from the Jim Crow South (Fusfeld & Bates, 1984; Kang 2024). Yet many of these populations were subsequently dispossessed or barred from coastal property ownership through treaty violations or discriminatory land-use policies and legislation (Aoki 1998; Moore et al., 2019; Jefferson 2020; Akins & Bauer, 2021). Most recently, a shift in the focus of the coastal economy from maritime industries to high-end tourism, luxury real-estate, and information technology has fueled rapid resettlement, establishing a "coastal curtain" where much of the shoreline is disproportionately white, wealthy, and older as compared to the state's diverse inland population (Reinman et al., 2016; Christen & King, 2017).

COMMENT: Furthermore, at the end of your Discussion, where you argue that "coastal adaptation planning can be used as a lever to redress historical inequalities", please expand on how and talk to the aspects only outlined in Table 3 at this stage. It would be great to see some specific recommendations following from your review in the main text and study and have a brief discussion attached to each.

RESPONSE: Please see response to previous comment. The Discussion of the revised manuscript has been expanded significantly in order to provide the specific examples requested by the reviewer and better ground them relevant theory and empirical evidence.

COMMENT: The results and methods would also benefit from further analysis of the limitations of the research approach, for example expanding on what you did to better access inland areas and how histories of gentrification/increase in prices, forced removals, barriers to ocean access would further impact people's access to live by the ocean and how this would have impacted your study "sample".

RESPONSE: In response to this comment, and parallel comments made by Reviewer(s) 5 & 6, a new section has been added to the Discussion (i.e., *4.3 Study Limitations and Next Steps for Research*) which explicitly addresses the limitations of the study. This text, found on Lines 467-487 of the revised version of the manuscript reads as follows:

It is important that our findings be interpreted in light of several limitations. Despite our intentional sampling efforts to reach disadvantaged and underrepresented communities in coastal counties across central California, the composition of our final sample resulted in the overrepresentation of some demographics (i.e., Asian & Native Hawaiian or Pacific Islander) and the underrepresentation of others (i.e., Hispanics and Latinos) as compared to California at-large. Likewise, though we attempted to mitigate such dynamics by disseminating the survey online and sampling inland community hubs (i.e., laundromats, community events, markets, etc.) in addition to coastal access points, field work focused on disadvantaged zip codes within coastal counties likely failed to capture many of the dynamics most relevant to California's

diverse (and by many metrics, most vulnerable) inland population. In addition, though we believe our research design was robust (as grounded both in relevant literature and the lived experience of the communities that provided data; see Methods) we acknowledge that a) our assessment of many nuanced and multidimensional constructs was ultimately constrained by the number, detail and presentation of the underlying prompts; and b) a degree of simplification was required to accommodate the breadth of the complete survey instrument. Finally, though our analysis of cross-sectional survey data serves as a valuable, baseline description of current conditions, additional research is required to definitively determine and evaluate causal pathways. Future research designed to address such aims would be well-served in considering longitudinal study design and/or experimental interventions, objective (rather than self-reported) measurements, and increased integration with qualitative inquiry (Biesbroek et al., 2017; Kleiber et al. 2024).

COMMENT: It would also be good to know why the specific survey prompts were used, and who these related to the theoretical framework.

RESPONSE: Please see response to subsequent comment made by Reviewer #2 describing the logic motivating the revision to an expansion of the information presented in what was previously labeled **Supplemental Table 1**.

Line-by-line comments

68-69 - include reference here

In the revised version of the manuscript, this statement is supported by the two references copied below:

-Allison, E.H., Kurien, J., Ota, Y., Adhuri, D.S., Bavinck, J.M., Cisneros-Montemayor, A., Fabinyi, M., Jentoft, S., Lau, S., Mallory, T.G. and Olukoju, A., 2023. The human relationship with our ocean planet. In *The blue compendium: from knowledge to action for a sustainable ocean economy* (pp. 393-443). Cham: Springer International Publishing.

-Costanza, R., 1999. The ecological, economic, and social importance of the oceans. *Ecological economics*, *31*(2), pp.199-213.

72 - relationship with “and access to”?

We thank the Reviewer for this clarifying suggestion and have made the requested edit.

78-81 - good!

We are thankful for the Reviewer’s positive assessment of this text.

94 - it would be good to elaborate a bit more on the California Coastal Act for people who are not familiar - and also what the “recent progress” refers to.

To provide the additional information and clarification requested by the Reviewer, the text on Line 67-69 of the revised version of the manuscript has been modified so that it now reads, *"Despite the progressive mandate of the California Coastal Act (which secured legal protection for public access in 1976) and other recent legislation (i.e., the Marine Life Management and Marine Life Protection acts), the regional mosaic of ocean health…"*

91-99 - great!

We are thankful for the Reviewer's positive assessment of this text.

119 - "modern development scholars" I am unsure these authors would characterize themselves as such - please explain what is meant here and why this is used. As mentioned above, I also think the social benefits are not necessarily poorly studied, although they are often overlooked and neglected.

Please see responses to a previous comment made by this Reviewer (in which we describe the logic driving a revision to Lines 35-43 of the Introduction to more clearly articulate the research gap addressed by the study and better acknowledge existing literature) and subsequent response to Reviewer 2 (in which we describe how the "Theoretical Framework" section has been restructured to better describe and motivate the linkages between theories of access, wellbeing, and equity).

176 - please expand a bit on why you think it is that individuals traveling longer distances were likely to reside in more vulnerable communities. In the introduction it would be great with more detail on the context and who you reached (and perhaps did not reach) with this survey.

In following text has been added to Line 183-184 of the revised version of the manuscript in order to better elucidate the connection advanced by the Reviewer: "*Consistent with coastal gentrification, individuals having to travel longer distances to access the ocean were likely to reside in more vulnerable communities…*". While we are hesitant to add too much interpretation to the results text, we hope that this brief phrase is sufficient to reinforce the role of historical, demographic change (described in detail in a new Introduction paragraph, see above) in shaping the observed patterns.

200 - the strongest correlation with frequency with people identifying as White - it would be good to hear more about what this means in the analysis - and the socio-economic and/or historical factors that might have impacted this.

Please see response to the comment above. We hope that by adding a new paragraph to the Introduction (more specifically the text on Lines 56-59 which reads, "*Most recently, a shift in the focus of the coastal economy from maritime industries to high-end tourism, luxury real-estate, and information technology has fueled rapid resettlement, establishing a "coastal curtain" where the shoreline is disproportionately white, wealthy, and older as compared to the state's diverse inland population*") that this connection becomes self-evident.

Table 1 - prompt 5. “I possess the required skills or knowledge” - firstly, how was this elaborated and conceptualised? It would be good to see how this was explained to respondents. Secondly, what about being able to contribute your own knowledge to ocean activities and/or decision-making, and feeling heard? It would be good to include a short paragraph on why these eight prompts were chosen.

We have added the following text to the methods section on Line 669-676 of the revised version of the manuscript to clarify that, “*The prompts included in each scale and sub-scale were iteratively developed and assigned using a) a review of relevant literature (see **Supplemental Table 1** and **Supplemental Table 2**), b) initial scoping discussions, designed to explore issues and dynamics of local relevance across our study area, conducted with community partner organizations and other local leaders (n = 10); and c) feedback obtained during survey piloting*.” As referenced in Line 584-586 of the revised version of the manuscript, readers seeking additional detail concerning the presentation of prompts can access the final survey instrument online where it has been attached as Supplemental Information. While we agree an analysis of agency in the contribution of knowledge and participation decision-making would be informative (as partially assessed in Survey Questions #20 & #21), we ultimately believe it is beyond the scope of the current manuscript where some degree of simplification was required for narrative clarity and cohesion.

246-248 - it might be good with a footnote briefly explaining ANOVA, post-hoc Turkey HSD and OLS

Following the Reviewer’s request for additional methodological detail, we have made two insertions in the methods text. Line 657-662 now reads, “*Differences between sub-scales were assessed using by grouping individual, complete responses (n= 1600 for Barriers prompts; n= 1568 for Wellbeing prompts), using a repeated measures ANOVA (which tests for the significant differences between the means of three or more groups, where the same subjects are measured under each condition) and a post-hoc Tukey HSD test (performed to identify exactly which pairs are different while controlling for the family-wise error rate) (Zar 1996).*” Line 680-681 now reads, “*we used Ordinary Least Squares (OLS) regression (a method to estimate the linear coefficients of our predictors by minimizing the sum of squared residuals between the observed and predicted values; see Zar 1996)*”.

304-306 - again, it would be good to know up front a bit more about the demographics and historical context determining who might live near or far away from the coast

Please see our response to a previous comment made by this Reviewer and the text insertions on Lines 44-59 of the Introduction.

323 - perhaps in one sentence restate how community vulnerability was conceptualised here

Following the reviewer’s suggestion the text on what is now Line 343-344 of the revised version of the manuscript has been modified so that it now reads, “*Community vulnerability (conceptualized here as the product of environmental exposure to pollution and socioeconomic sensitivity; see Methods)....”*

356 - historical legacies of discrimination and dispossession - this should be considered in the beginning of the paper

Please see our response to a previous comment made by this Reviewer and the text insertions on Line 44-59 of the Introduction.

362-368 - same as above, should be mentioned in the Introduction when introducing the specific context in which you are working in

Please see our response to a previous comment made by this Reviewer and the text insertions on Line 44-59 of the Introduction.

370 - “may be” - I think it is, no?

Following the Reviewer’s suggestion, we have revised the sentence now found on Line 416 of the revised version of the manuscript and replaced “may be” with “is”.

374 - “may limit” - it is quite clear that it does?

Following the Reviewer’s suggestion, we have revised the sentence now found on Lines 412 of the revised version of the manuscript and eliminated the word “may”.

377 - gender dimensions receiving less research attention - please be specific - less than what? Where? By whom?

This text now found on Line 419 of the revised version of the manuscript has been modified to provide the clarification requested by the Reviewer and add the two supporting references copied below. The text now reads, “*Although the gendered dimensions of ocean access and wellbeing have received less research attention as compared to terrestrial conservation and agricultural sectors…*”

-Cruz-Garcia, G.S., Sachet, E., Blundo-Canto, G., Vanegas, M. and Quintero, M., 2017. To what extent have the links between ecosystem services and human well-being been researched in Africa, Asia, and Latin America? *Ecosystem services*, 25, pp.201-212.

-Harper, S.J., Gurney, G.G., Darling, E., Mangubhai, S., Jupiter, S., Lestari, W.P., Holmes, K.E., Sumaryati, S., Sulisyati, R., Fox, M. and Ban, N.C., 2024. Gender differences in the perceived impacts of coastal management and conservation. npj Ocean Sustainability, 3(1), p.34.

378 - benefitting from ecosystems - but also ocean specifically?

The text on Line 419-421 of the revised version of the manuscript has been modified so that it now reads, “*...our results support the assertion that gender is a critical determinant of how people differentially access and benefit from ocean ecosystems (Quinn & Davis 1997; Kleiber et*

al., 2014)." The non-specific reference used to support this assertion previously has been replaced by two specific and ocean focused case studies (see below).

-Quinn, N.J., and Davis, M.T. 1997. The productivity and public health considerations of the urban women's daytime subsistence fishery off Suva Peninsula, Fiji. South Pacific J. Nat. Sci. 15: 63–92.

-Kleiber, D., Harris, L.M. and Vincent, A.C., 2015. Gender and small-scale fisheries: a case for counting women and beyond. *Fish and Fisheries*, *16*(4), pp.547-562.

382 - Again, the act should be further introduced when first mentioned

Please see response to the previous comment made by this reviewer regarding the clarifying text insertion that can be found on Line 67-69 of the revised version of the manuscript.

386 - please explain what is meant by recognitional justice here

We take the reviewer's point (and a subsequent point made by Reviewer 2) that introducing additional terminology at this stage of the manuscript is likely to serve only to muddy the waters. We have changed the anchoring concept of this phrase from "recognitional justice" to "recognitional equity", which is now formally defined in the Theoretical Framework section of the revised manuscript.

402 - expanding body of literature - also in the marine social sciences, ocean governance +++ literature

On Line 443-445 of the revised version of the manuscript, we have modified the text to include references to parallel and supporting arguments made by the disciplines suggested by the reviewer. This text now reads, "*This assertion is supported by an expanding body of public health (Fleming & Landrigan, 2024; Garrett et al., 2019) marine social science (Poe et al., 2014; Strand et al., 2022; Allison et al., 2023) and ocean governance (Bennett 2022; Österblom et al., 2023) literature, which suggests that broader and more inclusive management strategies are required to elevate the diverse forms of ocean engagement that support relational and subjective wellbeing.*"

407-408 - I do not think you are demonstrating that "such benefits are not broadly understood" - you seem to be overlooking a very broad set of literature and scholarship. See Ingersoll or other authors mentioned above.

On Line 440-441 of the revised version of the manuscript, we have modified the text highlighted by the reviewer to better articulate the scope and origin of the focus we are characterizing as narrow. This text now reads, *"Our findings suggest that the narrow focus of blue economy initiatives (and the Western management frameworks in which they are embedded) overlooks important wellness benefits and constrains the ocean's benefits to society."*

411 - specify that this is in California. Important to reflect on where you did your survey and what it tells you. There is definitely lessons to be drawn more broadly, and it would be great if you could bring in some literature looking at similar aspects in various contexts.

See response to a previous comment made by this Reviewer and a parallel comment made by Reviewer (s) 5 & 6. In revising Section 4.3, we have attempted to pull in additional examples from the greenspace literature and reflect upon the similarities and divergences presented in that work and our own.

415-419 - I would argue a lot of environmental justice approaches go beyond physical infrastructure and/or economic opportunity. Please provide references, and consider looking a bit deeper into the literature.

We thank the Reviewer for their attention to detail here and pointing out the erroneous framing of the previous text. In the revised text (now found on Lines 461-462), rather than critiquing EJ frameworks, we suggest that other management approaches could learn from them in seeking to advance more holistic and effective approaches to supporting human wellbeing. This text now reads, "*By drawing upon environmental justice frameworks that prioritize recognition and relational values (Scholsberg 2007; Poe et al., 2014; O'Neill et al., 2025), coastal management approaches designed to mitigate climate impacts or redress environmental degradation should move beyond efforts to provide physical infrastructure and/or economic opportunity to explicitly consider the social, cultural, and emotional pathways through which the ocean supports human wellbeing.*"

422-423 - California considered a world leader - According to whom and what metrics?

We have modified this text, now found on Line 490 of the revised manuscript, to clarify "*California considers itself a world leader…*"

Table 3 - Please include these in the main text and speak to them in more detail. Has this been implemented to some extent already, and in that case by whom? Who should be doing this, and where should the budget come from? What are actions or recommendations at different levels (personal, community, city) or tiers of government?

Please see response to previous comment made by this Reviewer. We have revised and expanded Section 4.4 significantly to parse the example recommendations in the main text.

434 - the survey results did not seem to speak much to women and non-binary people - can you expand here if they did?

Please see response to subsequent comment made by Reviewer(s) 5 & 6 regarding how gender is presented and unpacked in the analysis.

435 - how do you define ocean justice?

This text has been removed from the revised version of the manuscript.

442 - what do you mean by equity-centered approaches?

This text has been removed from the revised version of the manuscript.

446 - could you be more specific? How can and should coastal management and policies redress racial disparities when it comes to ocean access and benefits?

Please see response to previous comment made by this Reviewer. We have revised and expanded Section 4.4 significantly to parse the example recommendations in the main text.

454 - what might be a limitation of intercepting at coastal access points? Just thinking of access…

See previous response to a comment made by this Reviewer. The revised text includes a new discussion section (*4.3 Study Limitations and Next Steps for Research)* where this and other study limitations are discussed in-depth.

464-470 - it would be great to mention this up front in the paper

We would direct the Reviewer's attention to the manuscript's abstract where the research is presented as being developed using a community-engaged research approach. While we agree that it might be useful and informative to provide additional detail in this regard in the main text (indeed, this is the aspect of the research that we are most proud of!), we ultimately did not feel that it would be possible without restructuring and expanding the Introduction text significantly.

Reviewer #2

COMMENT: This paper examines the factors that mediate coastal access and the ocean's contributions to wellbeing with a focus on California. It is a well conceptualized study and well written paper that presents novel empirical results. The topic is important and will be of interest to a broad audience of sustainability and ocean professionals and researchers. Overall, I would recommend that this paper be accepted subject to minor revisions. Below are suggestions to improve the paper, noting that none of them require new analysis - just some additional content, restructuring of paragraphs, providing headers, potential visualization of results, and concluding with a strong focus on access and wellbeing.

RESPONSE: We are grateful to the Reviewer for their positive assessment of our work and believe that following their recommendations in this revision has strengthened the manuscript significantly. Below we describe our efforts to a) better focus the theoretical framework and more explicitly describe how it informed the experimental design; and b) iterate data visualizations to improve clarity and impact.

Good introduction.

COMMENT: Section 2.0 - Theoretical Framework - at the end of the first paragraph it says that it draws on theories of ocean equity, access and environmental justice, and human wellbeing. Each of these needs to be unpacked a little more - explain a bit about each topic and how the study/framework links to them. Especially, ocean equity - which is a related topic, but I don't really see how this is integrated into the framework as it is not in the Supplemental Table 1.

RESPONSE: We thank the Reviewer for providing this thoughtful feedback and for prompting us to reconsider how we are defining the various components of our theoretical framework and conceptualizing linkages between them. Following additional text insertions in the Introduction designed to introduce the emerging field of ocean equity and its theoretical and ontological basis (see Lines 35-43 and response to a previous comment made by Reviewer 1) we have a) restructured and rewritten Section 2.0, emphasizing that our framework was developed to operationalize and interrogate distributive ocean equity; and b) modified the conceptual figure which it references (i.e., Figure 1) to explicitly include and center this concept. The revised text of Section 2.0 of what is now Lines 94-144 reads:

2.0 Theoretical Framework

*Marine conservation and governance scholarship have generally lagged behind their terrestrial counterparts in addressing issues of access, equity, and environmental justice (Boone et al., 2009; Byrne & Wolch, 2009; Walker 2009; Jennings et al., 2012) and their examination through an intersectional lens of race, class, and gender (Kaijser & Kronsell 2014, Malin & Ryder, 2018). Evolving from landmark research documenting the disproportionate toxic exposure of communities of color in the Southwest US (Bullard, 1990), environmental justice is a dynamic, overarching framework that advances a future where all people have the right to live in healthy and sustainable environments. This vision is realized through three interconnected components of equity: fair distribution of environmental benefits and burdens (distributional equity), meaningful participation in decision-making (procedural equity), and recognition of diverse social values, vulnerabilities and capabilities (recognitional equity) (Schlosberg 2007; Walker 2009; Jennings et al, 2012). In contrast, widespread acknowledgement of the value of such concepts in ocean governance and marine resource management is more recent, and scholarly debate concerning how they are best defined and assessed continues (Crosman et al., 2022; de Vos et al., 2023; Bennett et al., 2025). This study operationalizes and interrogates ocean equity through a two-step analytical process: first, by investigating access barriers as the structural mechanisms mediating ocean engagement; and second by measuring perceived contribution to human wellbeing as the primary indicator of the resulting distributive outcomes (**Figure 1**). While equity is a complex and multidimensional process, considered both as a characteristic of society in which governance occurs and an outcome of governance (McDermottet al., 2013; Crosman et al., 2022), here we primarily concern ourselves with distributional ocean equity (Bennett 2022; Bennett et al., 2025) rather than procedural or recognitional components.*

Western governance models (i.e., the Public Trust Doctrine, the California Coastal Act) typically manage ocean and coastal spaces through a universalist lens, assuming that physical access to coastal spaces directly translates into societal benefits. Yet the right to enter an environment and the ability to derive benefits from it are not the same. Drawing on theories of access, we suggest that the ability to benefit from ocean and coastal spaces is mediated by a bundle of powers influenced by physical-environmental, personal, social, and knowledge factors

*(Ribot & Peluso, 2003; Wang et al., 2014; Seto et al., 2024). Thus we consider barriers to ocean access not as random inconveniences, but as structural processes (i.e., persistent institutional, economic and social arrangements that shape individual agency) responsible for mediating this ability and determining the distribution of ocean benefits across the California coast (**Supplemental Table 1**).*

*Just as access can not be reduced to the right to physical entry, considering the benefits provided by ocean and coastal spaces requires moving beyond the neoclassical economic indicators emphasized by many modern management frameworks. Global discourse surrounding the 'Blue Economy' increasingly frames the ocean as a frontier for development, emphasizing material outputs such as food security, energy, and employment (World Bank & UNDESA, 2017; Costanza, 1999; OPC 2020). Yet, as human relationships with the ocean are varied and complex, marine social scientists and development scholars have advocated for a broader conceptualization of social benefits that considers the ocean's contributions to cultural and social identity, occupational pride and self-respect, spirituality, and mental and bodily health (Weeratunge et al., 2014; Allison et al., 2023; White, 2010). The wellbeing framework (**Supplemental Table 2**) embraces the holistic world-view of alternative epistemologies (Ingersoll, 2016; Lobo & Parsons, 2023) in considering the subjective and relational benefits of ocean ecosystems alongside those material dimensions most consistently emphasized by modern management frameworks. By coupling a multidimensional view of human wellbeing with a structural analysis of ocean access, we aim to map potential pathways and mechanisms informing how ocean equity (and inequity) are experienced on the ground.*

COMMENT: Also, regarding the framework - I can't see how it links to your survey methods - so would suggest to provide a table in the main manuscript that shows a) each main topic, b) the sub-attributes of that main topic that are covered in the survey, c) the survey question related to that attribute, and d) the scale used. Similar tables are provided in Hicks et al 2014 (Social, institutional, and knowledge mechanisms mediate diverse ecosystem service benefits from coral reefs) and Bennett et al 2020 (Social equity and marine protected areas: Perceptions of small-scale fishermen in the Mediterranean Sea). This provides a great quick reference.

RESPONSE: We thank the reviewer for this suggestion and for providing useful example templates. In response to their request to better link the theoretical framework to the survey methods, we have revised the previous **Supplemental Table 1** into what is now presented as **Supplemental Table 1** (containing the barriers material) and **Supplemental Table 2** (containing the wellbeing material). In each table, an additional column ("Survey Prompts") has been added to indicate which prompts were used in the assessment of which dimensional sub-scale. The following text has been added to the legends of what is now Line 953-954 & Line 956-957 of the revised manuscript to provide the requested scale information, "Responses to survey prompts were measured on a 5-point likert scale (Strongly Disagree = 1; Disagree = 2; Neutral = 3; Agree = 4; Strongly Agree = 5)." Noting that some of this information contained within these supplemental tables is redundant with **Figure 4** and **Figure 6** (which have been revised to improve clarity and address a subsequent comment made by this Reviewer and Reviewer(s) 5 & 6), we feel that this material is best suited for the Supplement rather than the main text.

COMMENT: For the results, it could really use sub-headers for each section within the results - to clearly separate the topics and make it easier for the reader to follow.

RESPONSE: Following the Reviewer's suggestion, the results have been broken up into 4 sub-sections labeled using the following sub-headers: *Demographic Factors Influencing Ocean Engagement (3.1), Structural Barriers to Ocean Access (3.2), Distribution of Wellbeing Benefits (3.3), & Processes and Pathways Shaping Distributive Equity (3.4).*

COMMENT: - Small edit, line 308-309 - "...was associated with significantly lower wellbeing..." should be "was associated with significant lower contributions to wellbeing..." (as the topic is "contributions to wellbeing", not just "wellbeing"). But, consider this elsewhere in the way the results are written - it is not wellbeing, but the level to which the ocean is contributing to wellbeing.

RESPONSE: We have made the requested edit.

COMMENT: Figure 4 - Is the result for 'Distance' missing from Figure 4a at top left?

RESPONSE: As is referenced in the Figure legend, not all models include the same predictors as the structure of each model was determined by forward stepwise model selection using AIC criteria. Distance was dropped from the composite barriers model as adding it in as a predictor variable did not improve model fit. In order to avoid any similar confusion on the part of other readers, the figure legend on Line 278-279 of the revised version of the manuscript has been modified, providing Distance as an illustrative example, so that it now reads "*Note that not all models include the same predictors (e.g., Distance was not included in the composite model illustrated in panel* ***A****) as the structure of each was determined by forward, stepwise model selection using AIC criteria (see Methods)*." A similar edit (on Lines 356-357 of the revised version of the manuscript) has been made to the legend of **Figure 7**, which presents the wellbeing model and sub-models.

COMMENT: For a couple of the results tables in the paper, I was wondering whether there was a better way to visualize them. For consideration. However, I do understand that a combination of figures and tables is good.

RESPONSE: We agree that some of the results could be better visualized, particularly those presented in Table 1 and Table 2 of the previous version of the manuscript. Following this suggestion, and responding to a comment made by Reviewer(s) 5 & 6, we have revised these two tables and broken out the key result of each into a figure panel. In the revised version of the manuscripts, Figure 5B and Figure 6B use dot-whisker plots to illustrate the Item-Rest Correlation values of prompts associated with each scale dimension, highlighting which dimensions were most effective in measuring the core construct of composite scales. For more information concerning this revision and the text insertions with which it is associated, please see subsequent response to the comment made by Reviewers 5 & 6.

COMMENT: There are some long paragraphs throughout the results and elsewhere in the paper that are too long, and hard to follow because there is not clear topic sentence. Suggest to go through and break these up and give them more structure.

RESPONSE: Where possible, throughout the manuscript we have tried to break up compound sentences into simpler, shorter components and refine our use of topic sentences to better direct and retain the reader's attention.

COMMENT: For the discussion, most of it is good - but it is really weird to have the last paragraph suddenly being about "ocean justice" in policy and practice. Why introduce this new jargon, it does not help - and instead confuses this reader. I would keep this focused on the central topic of this paper - ACCESS. So, I would suggest to reframe this section to be about "Advancing ocean access and human wellbeing through policy and practice" - and really dig in on the path to increase the ocean's contributions to the wellbeing of all citizens through a focus on access for all people. It has been very focused - finish strong!

RESPONSE: We take the Reviewer's point that introducing new terminology at this point in the manuscript may serve only to muddy the waters. This final section has been renamed following the reviewer's suggestion. While the revised text does include a brief description of relevant environmental justice considerations, as requested by Reviewer 1 and Reviewer(s) 5 & 6 (see previous and subsequent responses), the primary focus of the revised manuscripts second to last paragraph is a return to theories of access and human wellbeing. We believe this framing to be very useful in better contextualizing and interpreting the study's empirical results. That text, on what is now labeled Lines 523-540 reads as follows:

"Our findings suggest that the primary crisis of ocean equity may no longer be solely about subsistence and economic rights, but the fundamental human need to connect to, belong in, and access restorative blue spaces (Fleming et al., 2024). This aligns with the concept of 'realized access' which suggests that socio-personal dimensions of accessibility are as important as legal rights (Wang et al., 2015) and that cultural identity, social relationships and personal resources fundamentally mediate the ability to drive benefits from ocean resources (Seto et al., 2024). As compared to other regional research documenting barriers to urban greenspace access for minority and low-income populations (Wolch et al., 2005; Williams et al., 2020), fear of crime may not be a limiting factor in coastal access (i.e., most survey respondents reported feeling welcome in ocean and coastal spaces) yet personal barriers related to transportation access and geographic distance appeared comparably acute. Consequently, if the primary value of the coast for vulnerable communities lies in social cohesion, mental health, and spiritual renewal, then management bodies must invest in Coastal Infrastructure & Environmental Management which makes the use of local ocean areas for such purposes feasible, attractive, and affordable. In California specifically, agencies must prioritize more frequent and better integrated public transportation to pierce the coastal curtain and bridge the gap between isolated and/or urban communities and the coast. Where proximate shorelines are degraded or polluted, creative strategies may be required to remediate development, restore functional habitat, and transform neglected coastal fringes into community assets (Wolch et al., 2005)."

Reviewer #2 (Remarks on code availability):

I am not qualified to review the code for this paper.

Reviewer #3 & #4

Date: October 24, 2025
Review of: “Racial and Economic Disparities in Coastal Access and Engagement Mediate the Ocean’s Contribution to Human Wellbeing”
For: Nature Communications

Recommendation: Accept with minor revisions

General Comments:

Thanks for the opportunity to review “Racial and Economic Disparities in Coastal Access and Engagement Mediate the Ocean’s Contribution to Human Wellbeing”. This paper investigates equity aspects of ocean access in parts of California, using a definition of that term that expands beyond spatial/physical dimensions. The authors furthermore underpin their investigation with a framework for exploring barriers to accessibility, and their implications for human wellbeing.

I think this is a valuable contribution, providing a rich source of data that allows the authors to pick apart a wide variety of complex factors associated with ocean access and well-being. The conclusions reinforce and embellish a variety of observations about the structural factors leading to inequitable outcomes for low-income households and vulnerable communities. As elaborated below, I think the authors should do more to explain the specific implications of their analysis, both for the broader literature, and for policy makers.

RESPONSE: We are grateful for the Reviewer(s)’ positive assessment of our work and the opportunity to refine and improve it through their insightful comments. Following parallel comments made by the other Reviewers, a significant focus of our revision has been to expand upon the significance of our results (both in terms of their theoretical contributions to related literature and the specific guidance that they provide for policymakers). Please find additional detail in the responses copied below.

A note about my own expertise. While I was largely able to follow the analytical steps outlined in this paper, I am not able to assess the soundness or validity of the statistical calculations reported in detail, and it is important that this be done by another reviewer.

I have a few high-level comments and questions, followed by some minor details that I noticed while reading.

COMMENT: Implications of the study. Overall, I think the authors could do more to explain how this study and analysis changes and/or advances our ability to actually address the systemic problems that they are reporting on. I appreciate that the paper lends analytical heft to existing criticisms of the material/economic framing of ocean benefits, but it would be great to see this unpacked in a couple of different ways:

Rather than just reporting how these results are consistent with other literature, the authors should explain what specific gaps or shortcomings in previous literature are addressed by this study’s results.

RESPONSE: We take the Reviewer(s) point that there is a need to better articulate the specific gaps the research was designed to address. On Line 35-43 of the revised and restructured Introduction we have devoted a paragraph to stating this gap and motivating the need to address it. This text now reads as follows:

"In recent years marine social scientists and commons scholars have advanced and refined theoretical frameworks describing the fundamental links between equity (defined broadly as the fair and just conditions and treatment of people), governance, and ocean sustainability (Crosman et al., 2022; Österblom et al., 2023; Bennett et al., 2025). Despite increasing recognition of the importance of equity considerations in the durability, legitimacy, and efficacy of coastal management, their assessment and operationalization remain an enduring challenge (Crosman et al., 2022). Empirical, place-based investigations concerning the structural factors mediating who is able to access and benefit from ocean ecosystems (and why) remain scarce, particularly across highly-developed, urbanized and/or multicultural coastlines characterized by complex and incompletely understood patterns of use and proximity."

In addition, throughout the text (and in the Discussion in particular) we have tried to pull in additional citations describing previous empirical work and reflect upon to what extent our mirror or diverge from theirs. Acknowledging the research gap articulated above while following a suggestion made by Reviewer(s) 5&6, many of these new references are based on terrestrial conservation and greenspace literature (where engagement with issues of equity, access, and environmental justice has to-date been more extensive). Two examples of such text, found on and Lines 505-508 and Lines 528-532 of the revised version of the manuscript are copied below.

"Compared to green space, the impact of knowledge on ocean access may be comparatively more pronounced as many forms of engagement and participation require ocean literacy and water competence, a specific set of physical skills and safety knowledge often informally transmitted and unequally distributed across society (Wiltse 2014; Strand et al., 2023)."

"As compared to other regional research documenting barriers to urban greenspace access for minority and low-income populations (Wolch et al., 2005; Williams et al., 2020), fear of crime may not be a primary limiting factor in coastal access (i.e., most survey respondents reported feeling welcome in ocean and coastal spaces, though ***Supplemental Figure 6*** *suggests this was somewhat less true for minority populations) yet personal barriers related to transportation access and geographic distance appeared comparably acute."*

COMMENT: The policy recommendations in Table 3 are very straightforward, and would have seemed quite sensible and important if put forward on their own (i.e., without this study backing them up). Are there any new/different policy recommendations that this analysis points to, which might not have been on the table otherwise?

RESPONSE: Please see response to previous comment made by Reviewer 1 & Reviewer(s) 5 & 6. We acknowledge that in the previous version of the manuscript the recommendations presented in **Table 3** were presented in a cursory manner which served to limit

their usefulness. In the revised version of the manuscript we have significantly expanded and restructured the text which presents this Table 3 (i.e. Section 4.4. on Lines 501-559), trying to more clearly identify and describe the experimental results to which each recommendation is linked and how their potential utility can be contextualized by referring to other relevant literature.

COMMENT: Finally, I'd like the authors to address the relevance of these results beyond California. Specifically, is there anything unique about California that would suggest caution in extending these results to other areas, or even extending this very same analytical approach? The importance of this question is perhaps reinforced by the major differences found between southern and northern locations.

RESPONSE: We agree that addressing the generalizability of our findings is of critical importance. In response to this comment and parallel suggestions made by Reviewer 1 (concerning the importance of providing relevant historical and socioeconomic context upfront) and Reviewer(s) 5 & 6 (concerning the need to more clearly address the limitations of the study) we have added two new sections to the manuscript. In the revised version of the manuscript, Lines 44-59 describe the historical context shaping our investigation while Lines 467-487 of the Discussion (i.e., 4.3 Study Limitations and Research Next Steps) discuss the methodological constraints limiting our power of inference. In this text, we attempt to acknowledge that while the specific patterns quantified by our analysis in Central California are a product of the unique, multicultural nature of our study population (and the geographically distinct waves of migration, dispossession, and exclusion by which it was shaped), the underlying mechanisms through which structural barriers function to constrain access and wellbeing (and the analytical approach we have developed to identify and interrogate them) are of broad theoretical relevance. Indeed, we argue that the differences found between our study's Northern and Southern locations ultimately serve to demonstrate the framework's high sensitivity to local context and complexity, a necessary trait for extending this approach to other diverse global contexts.

Specific Comments/Edits

Line 199 -- Typo in "Norther"

We have made the requested edit.

Line 215 -- "Choose" should be "chose"

We have made the requested edit.

Line 296 -- Clarify what you mean by "which are the focus of most management and policy efforts"

Though the text on the previous versions of manuscript has eliminated following some restructuring, a parallel insertion made on Line 131-133 of the theoretical framework section clarifies, "*Just as access cannot be reduced to the right to physical entry, considering the*

benefits provided by ocean and coastal spaces requires moving beyond the neoclassical economic indicators emphasized by many modern management frameworks…"

Line 464 -- Missing word: in-depth "in" the context of…

We have made the requested edit.

Reviewer #5 & #6

COMMENT: The paper addresses a significant and important area of research and contributes novel findings. The work can feed into global debates on ocean access and wellbeing and provides important contributions to discussions of barriers to ocean use, having practical implications and potential for impacts to address inequalities. We congratulate the authors on identifying a key area for further research and using a large and diverse sample which pays attention to representation. The paper is generally well written and easy to follow.

RESPONSE: We are grateful for the Reviewer(s) positive assessment of our work and believe that our efforts to address their feedback have strengthened the manuscript significantly. In particular, we would like to thank the Reviewer(s) for encouraging us to dig deeper into terrestrial conservation and greenspace literature, which we feel has been a significant benefit to strengthening our theoretical framework and better contextualizing our contribution within broader scholarship concerning equity and environmental justice. Please find more detail regarding the specific revisions undertaken in the responses copied below.

COMMENT: The paper starts with a clear introduction to the knowledge gaps that exist on barriers that mediate access and benefits from ocean ecosystems and the need for further research in this area. Research to date has typically focused on the barriers to access and benefits related to green space and the intersectionality related to this, and so it is novel to see this research focused on oceans, which is particularly important as it has been demonstrated that access to oceans and seas can have significant benefits for wellbeing. Whilst the introduction is well written it would be good to acknowledge some of the research that has been done on green space, to highlight that access and barriers to environmental spaces are not felt evenly across publics and whilst there is increasing recognition of this for green space there has been little/no attention to this for oceans/seas. Furthermore, terms such as structural barriers, compounding, cascading, and justice/climate justice, environmental justice, recognitional justice, ocean justice - would benefit from clearer definitions, especially if they play a central role in the argument.

RESPONSE: Please see previous comment to Reviewer #2. In the revised manuscript the Theoretical Framework text has been rewritten to provide more of the definitions, theory, and context provided by the Reviewers. With reference to better acknowledging the foundational work of environmental justice, green space, and terrestrial conservation scholars, we direct the reviewer to previously and subsequently discussed text insertions made on Lines 95-113, Lines 396-398, Lines 417-419, Lines 461-462, and Lines 528-533 as supported by the references copied below.

-Boone, C.G., Buckley, G.L., Grove, J.M. and Sister, C., 2009. Parks and people: An environmental justice inquiry in Baltimore, Maryland. Annals of the association of American geographers, 99(4), pp.767-787.
-Bullard, R.D., 2018. *Dumping in Dixie: Race, class, and environmental quality*. Routledge.
-Cole, L. W., & Foster, S. R. (2001). From the Ground Up: Environmental Racism and the Rise of the Environmental Justice Movement. New York University Press.
-Jennings, V., Johnson Gaither, C. and Gragg, R.S., 2012. Promoting environmental justice through urban green space access: A synopsis. Environmental justice, 5(1), pp.1-7.
-Kaijser, A. and Kronsell, A., 2014. Climate change through the lens of intersectionality. Environmental politics, 23(3), pp.417-433.
-Malin, S.A. and Ryder, S.S., 2018. Developing deeply intersectional environmental justice scholarship. Environmental Sociology, 4(1), pp.1-7.
-McDermott, M., Mahanty, S. and Schreckenberg, K., 2013. Examining equity: a multidimensional framework for assessing equity in payments for ecosystem services. Environmental science & policy, 33, pp.416-427.
-O'Neill, B.F., Schneider, M.J. and Lozano, A.G., 2025. Toward a critical environmental justice approach to ocean equity. Environmental Justice, 18(2), pp.90-99
-Schlosberg, D., 2007. Defining environmental justice: Theories, movements, and nature. OUP Oxford
-Wolch, J., Wilson, J. P., & Fehrenbach, J. (2005). Parks and Park Funding in Los Angeles: An Equity-Mapping Analysis. Urban Geography, 26(1), 4–35.
-Williams, T. G., Logan, T. M., Zuo, C. T., Liberman, K. D., & Guikema, S. D. (2020). Parks and safety: a comparative study of green space access and inequity in five US cities. Landscape and Urban Planning, 201, 103841.
-Walker, G., 2009. Beyond distribution and proximity: exploring the multiple spatialities of environmental justice. Antipode, 41(4), pp.614-636.

COMMENT: The paper uses the state of California, USA as a case study. California is a state known for biodiversity, cultural significance and a leader in the protection and management of ocean environments, and so represents a very appropriate case study. The study administered 1691 survey to learn about the factors mediating ocean use and benefits with a focus on wellbeing. The work has paid attention to include typically underrepresented demographics, as would be expected from a study with these objectives, however we recognise the difficulties in doing this in practice and the authors should be congratulated for this. Those who identified as Hispanic or Latino seem slightly less represented than the California population (29.1% of the survey compared to compared to 39.4% within the population) and whilst I acknowledge the difficulties of ensuring proportional representation the difference should be acknowledged and discussed within the paper. There is no discussion of limitations of the work within the paper and this should be addressed.

RESPONSE: See previous response to a comment made by Reviewer #1. The revised text includes a new discussion section (*4.3 Study Limitations and Next Steps for Research)* where this and other study limitations are discussed in-depth.

COMMENT: The paper develops a framework to conceptualise the barriers to ocean access and the ocean's contributions to human wellbeing. This, and how it relates to the survey instrument developed, is an important contribution, as this can be adapted for use in other regions and the significance and potential for transferability could be highlighted more within the paper, i.e. the results are of use for all, not just to California, and more discussion of potential implications outside of California would be good. Expanding the discussion on theories of ocean equity, access, environmental justice, and human wellbeing used in the framework (either in the theoretical framework section or the supplementary info if the word count does not allow) and moving figure 1 to within the theoretical framework section would improve the paper.

RESPONSE: We thank the reviewer for this suggestion and prompting us to reflect on how the presentation of the Theoretical Framework could be revised to enhance its significance and transferability. Consistent with this comment, and parallel comments made by Reviewer #1 we have rewritten and restructured the introduction to better acknowledge the foundational contributions of previous literature concerning ocean equity and the social and cultural benefits of human relationships with the ocean to our study; b) clearly articulate the specific research gap that our study was designed to address. Consistent with this comment and parallel comments made by Reviewer #2 we have a) restructured and rewritten Section 2.0 better described how we are defining the concepts most essential to our study and conceptualizing the linkages between them (i.e., structural barriers to access, wellbeing benefits, and distributional equity) and b) modified the conceptual figure which it references (i.e., **Figure 1**) to explicitly include and center distributional equity.

COMMENT: The results of the survey are well analysed and generally clearly presented, demonstrating how geographic, gender, race, and socioeconomic predictors intersect to influence ocean access and contributed to human wellbeing which is segmented into material, subjective, and relational wellbeing. Figure 3, is well presented. However, is it striking that female has been removed (why not male!), I understand that that is because "variables contained redundant information". Therefore, it would be more accurate to represent this as binary (male/female) and trans/non-binary, it would also save a redundant line in the figure. This description is also necessary for other figures when female has been omitted e.g. Fig 5 and Supplemental Figure 4.

RESPONSE: We agree with the reviewer that the presentation of the gender variables in the referenced Figures can be improved. While we originally opted to present "Male" rather than "Female" as the effects in some of the models were more marginally more pronounced (i.e., 0.069 *** vs. -0.56** in the composite Barriers model), upon further consideration we have opted to 1) Alternate which of the two collinear variables are presented in each figure (i.e., **Figure 5** presents the final models which include the "Male" dummy variable and **Figure 7** presents the final models including "Female" dummy variable); and 2) Include alternate versions of what are now labeled **Figure 5**, **Figure 7**, and **Supplemental Figure 6** in the Supplement (i.e., **Supplemental Figure 4**, **Supplemental Figure 5, and Supplemental Figure 7**) which present alternate versions of the analyses w/ the omitted collinear variable. We thank the reviewer for prompting us to address this issue as we feel that the revised text now contains substantially more information for those readers wishing to consider the analysis through the lens of gender.

COMMENT: In Fig 3 Native Hawaiian or pacific islander has no results shown, is this because the sample is too small? If so this should be discussed and/or perhaps mention in the description.

RESPONSE: In **Figure 3** no results are shown for Native Hawaiian or Pacific Islander because none of the correlations exceeded the significance thresholds used to delineate which associations were the strongest and most significant (i.e. $p < 0.05$). While it is true that the sample size for this group was among the smallest of all the racial and ethnic demographic groups included in our analysis (i.e., 3.1% of the sample, as described in Line 152 of the revised version of the manuscript), we are not able to determine whether the null results for Native Hawaiian or Pacific Islander in Figure 3 are a product of a limited sample or the diversity of individuals and demographic characteristics within that sample. When looking at all the correlation values regardless of significance, the highest value is 0.08 (Native Hawaiian or Pacific Islander::Community Vulnerability).

COMMENT: Table 1 is very important for the paper's findings and needs careful explanation. The paper states that knowledge factors are the "most influential in mediating differences in the overall barriers score". However, the figure description states that "smaller mean values are associated with greater reported barriers", yet physical environment barriers have a smaller mean (3.81 compared to 3.89). This needs checking or the information used for interpreting the figure clarified. The sentence "mean item total correlation coefficients suggest that knowledge factors (0.7 ± 0.01), were most influential in mediating differences in the overall barriers score" is also confusing because it uses 0.7 ± 0.01 and my understanding is that this figure is only for prompt 8, if this is correct is should be explicitly stated, or the correct figure used. It would also be useful to highlight in the text the prompts which are contributing most significantly to the results as these are then referred to in the discussion. Similarly, for table 2.

RESPONSE: We acknowledge that Tables 1 & 2 and the presentation of the metrics contained within them were confusing in the previous version of the manuscript and thank the reviewer for prompting us to revise them for clarity, consistency and impact. We agree that the results as previously presented may have been muddled by presenting too many score and scale quality/reliability metrics in the main text and failing to clearly convey which each interpretation was based upon.

In the revised version of the manuscript, these Tables have been reformatted as Figures that present the mean (+/- S.D.) scale values for each prompt and sub-scale, as well as dot plots which illustrate the Item-Rest correlation values of individual prompts (points) points and sub-scale means +/- S.D (diamonds). Below we provide the revised Figure and Legend of what was previously presented as Table 1 in order to illustrate this approach.

A)

	Sub-Scale	Prompt	#	Prompt Mean (+/- S.D)	Sub-Scale Mean (+/- S.D)
Barriers to Ocean Access α = 0.83, ωt= 0.88	Social α = 0.54, ωt= 0.70	I feel welcome at beaches and coastal areas	2	4.33 (0.78)	4.10 (0.64)
		I feel protected by law or rule enforcers (e.g. police, rangers, security guards)	9	3.56 (1.04)	
	Personal α = 0.63, ωt= 0.68	I am interested in ocean experiences or activities	1	4.42 (0.80)	4.14 (0.72)
		I can afford or have access to necessary tools, gear, or equipment (e.g., fishing pole, surfboard)	6	3.74 (1.11)	
		I have access to and can afford transportation to the beach or coasts (e.g., bus, car)	3	4.25 (0.88)	
	Physical-Environ. α = 0.58, ωt= 0.59	The amenities/infrastructure I need or want are available	4	3.53 (1.10)	3.81 (0.75)
		I feel safe from environmental factors (i.e. sharks, waves, wind, pollution, etc.)	7	3.63 (1.02)	
	Knowledge α = 0.72, ωt= 0.73	I possess the required skills or knowledge to participate in ocean activities (i.e., swimming, fishing, surfing)	5	3.98 (1.04)	3.89 (0.80)
		I understand the associated rules and regulations (e.g., fishing rules, parking rules)	8	4.04 (0.92)	

B)

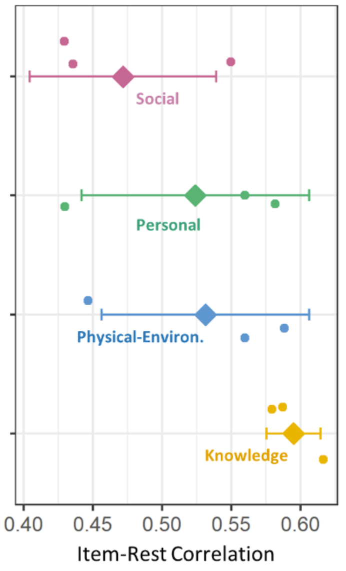

Figure 4. *Consistency and quality of survey prompts used to measure the composite scale of barriers to ocean access and its social, personal, physical-environmental, and knowledge dimensions. All prompts were measured using a 5-point Likert scale that was subsequently transformed into numerical values (i.e., strongly disagree = 1, strongly agree = 5). Panel (**A**) presents the i) Cronbach's alpha (α) and McDonald's omega (ωt) metrics that were used to measure the internal consistency of the entire scale (and each dimensional sub-scale; ii) the individual survey prompts contributing the composite scale (n=9) and each sub-scale (n=4; with color coding meant to indicate where prompts were considered in multiple sub-scales) and iii) the mean scale value (i.e., score) calculated for each prompt and sub-scale (n = 1600). Given the framing of the prompts, smaller mean values are associated with greater reported barriers. Panel (**B**) summarizes the Item-Rest correlation values for the prompts comprising each sub-scale, illustrating both the values of individual prompts (points) points and sub-scale means +/- S.D (diamonds). Larger Item-Rest correlation values indicate dimensions that were most effective at measuring the composite scale's core construct. Individual Item-Total & Item-Rest correlation values measuring the quality of each prompt in measuring the composite barrier scale and sub-scales can be found in **Supplemental Table 4**.*

Note that in this revision we have opted to present Item-Rest correlations graphically in the main text results rather than Item-Total correlations as they are considered to be a more accurate measurement of item/prompt quality. In order to clarify the logic behind presenting and interpreting Item-Rest correlation values the following text has been added to Lines 257-259 of the main text. *"…mean item-rest correlation values (i.e., the correlation between an item and the rest of the scale excluding that item, used here to evaluate which items were most effective in measuring the scale's core construct, see Methods)."*

Likewise, in order to provide a more exhaustive and detailed explanation, the following text has been added to Lines 670-676 of the methods. *"The quality of each prompt and subscale were assessed using Item-Total correlations (the correlation between and item's score and the sum of all item scores, including the item itself), Item-Rest correlations (the correlation between the item and the total score of the scale excluding that item) and their corresponding means and standard deviations. These metrics are useful in providing measurements of discrimination as*

items and/or prompts with the highest mean values are not necessarily the most informative if they are measuring a general concept that everyone agrees with."

In order to provide the specificity requested by the Reviewer concerning which prompts are contributing most significantly to the results, we have made two additional text insertions. With reference to the composite barriers scale, Line 262-265 of the revised version of the manuscript now reads, "*Indeed, across all 9 prompts, the first and second highest item-rest correlation values (i.e., understanding rules and regulations (0.61) & feeling safe from environmental factors (0.59); see* ***Supplemental Table 4)*** *were associated with knowledge dimension prompts.*" With reference to the composite wellbeing-scale, Line 316-318 of the revised version of the manuscript now reads, "*Across all 9 prompts, two of the three prompts with the highest item-rest correlation values (i.e., help me to feel part of a community (0.646), help me build relationships with friends and family (0.632)) were associated with the relational dimension."*

COMMENT: More careful attention needs to be paid to the discussion and how this relates to the results presented. The opening line states "Our findings reveal how racial identity, socioeconomic status, gender, and geography interact to shape ocean access and wellbeing". However, gender is not found to be significant in the results. Again it is stated "Although the gendered dimensions of ocean access and wellbeing have received less research attention, our results support the assertion that gender is a critical determinant of how people differentially benefit from ecosystems", I should imagine gender would be an important determinant but I do not see this reflected in the results that are presented and if this is a key finding the results need to highlight this.

RESPONSE: Perhaps this confusion can be attributed to uncertainty surrounding the way gender was defined as a demographic variable. Text has been inserted on Lines 606-608 of the Methods section to clarify that, "*Data obtained from demographic questions asking respondents to report race was transformed into a series of binary dummy variables to accommodate "Select all that apply" responses in which more than one category was chosen (i.e., a unique variable for 'Hispanic or Latino", in which responses including this category were marked as 1 and responses not including this category were marked as 0). Gender was assessed and coded similarly, though in this instance related variables were mutually exclusive (following the logic that individuals who selected more than one category could, by definition, be characterized as non-binary).*"

By way or further explanation, our results show that gender is a significant factor in predicting barrier to ocean access (indeed, identifying as Male was one of the strongest (0.069) and most significant ($p < 0.001$) predictors of all analyzed variables, as attributed to personal, physical-environmental, and (most acutely) knowledge factors. While gender did not significantly influence perceived contribution to human wellbeing when considering the composite scale, it did have a significant impact on the material wellbeing sub-scale (of all the predictors assessed, identifying as Male was the only predictor associated with greater perceived contribution to human wellbeing across any of the wellbeing dimensional subscales).

Note that an illustration error in the previous version of the manuscript (i.e., the inadvertent omission of the "***" significance asterisks next to "Trans or Non-binary" in what is now labeled **Figure 5A**), which may have contributed to this confusion, has been corrected. Though "Trans or Non-binary" was one of the more significant predictors in the composite barriers model, we choose to limit our analysis to Male vs. Female rather than Male/Female vs Trans/Non-binary given that skewed sample size of the latter comparison (n = 34 for Trans/Non-binary) limited our power of inference.

To reinforce this result, and better contextualize it within recent, relevant literature, Line 418-428 of the revised Discussion text now reads as follows, "*Although the gendered dimensions of ocean access and wellbeing have received less research attention as compared to terrestrial conservation and agricultural sectors (Cruz-Garcia et al., 2017; Harper et al., 2024) our results, support the assertion that gender is a critical determinant of how people differentially access and benefit from ecosystems*[37]*. Gender emerged in our analysis as one of the strongest predictors of barriers to ocean access, with females reporting significantly more barriers* ***(Supplemental Figure 4****) and fewer material benefits than male respondents (****Figure 7)****. Structural processes associated with political relations of ownership and control, differentiated social vulnerability, and gendered household roles*[37,38] *all likely interact in shaping varying levels of access and activity driving such variation. Note that while strong associations were also observed among transgender and non-binary respondents, we hesitate to draw broad inferences due to the limited (n=34) and geographically constrained sample of this demographic."*

COMMENT: Additionally, it is stated that "More recently, restrictive housing covenants and lending practices32, uneven investment in community infrastructure and environmental education33, and accelerating coastal gentrification1,34 continue to shape who feels safe, connected to, and welcome in ocean areas across the state". The safe and connected part of this sentence needs a reference, particularly as the results presented here (table 1 and post-hoc Tukey HSD) show that social barriers were not significant. I don't doubt that there has not been attention paid to social justice issues in housing practices etc. but more careful phrasing is needed to ensure distinction between suggestion for causes and clear results from the study.

RESPONSE: We agree with the Reviewer that the previous version of the sentence emphasized a dynamic that was not specifically highlighted by the results. The text on Lines 407-410 of the revised version of the manuscript has been modified so that it now reads, "*More recently, restrictive housing covenants and lending practices (Rothstein 2017), uneven investment in community infrastructure and environmental education (Finney 2014), and accelerating coastal gentrification (Reineman et al., 2016; Ounanian & Howells, 2024) continue to shape who is able to access and benefit from ocean areas across the state.*"

In addition, additional text has been added to Line 528-532 of the revised version of the manuscript to reflect on the impact (or lack thereof) of safety concerns as a barrier to ocean access, "*As compared to other regional research documenting barriers to urban greenspace access for minority and low income populations (Wolch et al., 2005; Williams et al., 2020), fear of crime may not be a primary limiting factor in coastal access (i.e., most survey respondents reported feeling welcome in ocean and coastal spaces, though* ***Supplemental Figure 6*** *suggests*

this was somewhat less true for minority populations) yet personal barriers related to transportation access and geographic distance appeared comparably acute."

COMMENT: You also state that significant barriers reported by Black and Asian American respondents, [..] are the result of structural processes embedded in racialized histories" – while this is highly likely to be true it is important to show how that was found based on the results, or to present this as a likely cause for the results you found. Greater engagement with existing literature on intersectionality in environmental studies could help here.

RESPONSE: We take the Reviewer's point that the language concerning causality should be softened as this inference goes beyond the scope of the manuscript's empirical results. Following their suggestion, the text on Line 397-403 of the revised version of the manuscript has been modified to clarify that this is an interpretation based upon and aligning with the frameworks advanced by intersectional environmental scholarship: "*Interpreted through the lens of intersectional environmental studies (Kaijser & Kronsell 2014, Malin & Ryder, 2018) significant barriers reported by Black and Asian American respondents, particularly knowledge (e.g., understanding rules and possessing necessary skills) and personal (e.g., lack of interest or affordability) barriers, reflect structural processes embedded in racialized histories. Existing literature cautions that what may appear as disinterest among such communities often functions as protective disengagement from spaces where they have experienced discrimination or marginalization (Wiltse 2014; Lee et al., 2023; Moulton 2024).*"

COMMENT: I enjoyed your discussion on overlooked benefits of relational and subjective ocean wellbeing. However, on line 411 it states, "By impeding ocean access", it may be that there is not an intentional impediment but more because of a lack of consideration. I think this should also be discussed because typically environment and social goals have been considered in isolation and results, such as yours, demonstrate that unless understood as intersecting then action for admirable environmental goals may have adverse and unequal social implications. A short discussion of how your results demonstrate the need to consider environmental and social aspects together, especially in coastal areas that will need to adapt to rising sea levels and changing climates, would demonstrate more clearly the significance of your work outside of California.

RESPONSE: We acknowledge and accept the Reviewer(s) comment concerning the value of additional reflection concerning the historical division between social and environmental goals, the value of integrated approaches, and the urgency and opportunity climate change considerations add to such dynamics. In the revised version of the manuscript, Lines 548-556 have been modified to present a concise synthesis of this argument which reads, "*While the emerging focus on ocean equity is a positive development, environmental justice scholarship suggests that a sustainable and equitable future may ultimately require reckoning with the market-liberal logics and hierarchies through which bourgeois environmental organizations and the global ocean governance system derive their present authority (O'Neill et al., 2025). Though social and environmental goals have traditionally been pursued in isolation, integrated approaches can help address the root causes simultaneously fueling class exploitation, social inequality and environmental degradation (Frawley et al, 2019). In this respect, global*

environmental change presents not only an existential threat for vulnerable coastal populations, but also a rare opportunity…..”

COMMENT: Your concluding sentence “Climate change presents not only an existential threat for vulnerable coastal populations, but also a rare opportunity for transformative change. With intentional design, coastal adaptation planning can be used as a lever to redress (rather than reproduce or exacerbate) historical inequalities51 and help build a future based upon universal ocean access, belonging, and wellbeing” is excellent and I applaud you on the research you are doing to contribute to this.

RESPONSE: We are grateful to the Reviewer(s) for their positive assessment of our effort.

In summary this paper addresses an important and novel area of research with a large data set of results. However, there are a few things to address before the paper is suitable for publication.

Specifically:

COMMENT: Line 1: Consider editing the title e.g. Uncovering racial and economic disparities in ocean access and contribution to wellbeing -this would highlight the transferability of the approach to other areas

RESPONSE: We thank the reviewer for this suggestion but would respectfully assert a preference for the Title used in the previous version of the manuscript.

COMMENT: Re-frame the introduction around the global significance of the work, i.e. need for greater research and investigation of coastal barrier as an environmental justice issue in general, then state the use California as a case study, i.e. the novelty and importance that these issues are researched and that California is a case study that will be used to do this but results are relevant for elsewhere to, e.g. the framework and survey instrument.

RESPONSE: Please see response to previous comments made by this Reviewer and Reviewer #1 which described the logic driving the restructuring of the Introduction and supporting text insertions.

COMMENT: Acknowledge past work on intersectionality even if this is only in alternative environments i.e. green space.

COMMENT: To better acknowledge past work on intersectionality and green space, the initial sentences of Section 2.0 (Theoretical Framework) on Line 95-105 of the revised text now reads,

“Marine conservation and governance scholarship has generally lagged behind its terrestrial counterparts in addressing issues of access, equity, and environmental justice (Boone et al., 2009; Byrne & Wolch, 2009; Walker 2009; Jennings et al., 2012) and their examination through an intersectional lens of race, class, and gender (Kaijser & Kronsell 2014, Malin & Ryder, 2018). Evolving from landmark research documenting the disproportionate toxic

exposure of communities of color in the Southwest US (Bullard, 1990), environmental justice is a dynamic, overarching framework that advances a future where all people have the right to live in healthy and sustainable environments. This vision is realized through three interconnected components of equity: fair distribution of environmental benefits and burdens (distributional equity), meaningful participation in decision-making (procedural equity), and recognition of diverse social values, vulnerabilities and capabilities (recognitional equity) (Schlosberg 2007; Walker 2009; Jennings et al, 2012). With widespread acknowledgement of the value of such concepts in the ocean sciences more recent…"

COMMENT: Expanding the discussion on theories shaping the theoretical framework and demonstrate how this relates to the survey instrument.

RESPONSE: Please see response to a previous comment made by Reviewer #2; the Theoretical Framework section has been restructured and rewritten while **Supplemental Table 1** (now **Supplemental Table 1** & **2**) have been revised to more clearly reflect the motivating theory and its connection to individual survey prompts.

COMMENT: Fig 3, 5 and Supplemental Figure 4 change male to binary (female/male)

RESPONSE: Please see response to previous comment made by the Reviewer(s).

COMMENT: Check the description and interpretations used in table 1. Consider highlighting the most significant prompts in table 1 and 2.

RESPONSE: Please see response to a previous comment made by the Reviewer(s). We have reformatted the information that was presented in what were previously labeled as Tables 1 & 2 and added text designed to identify and describe those prompts that were most effective in measuring the core construct of the composite scales. In addition, we have added parenthetical descriptions to Line 252-254 and Line 306-310 of the revised version of the manuscript to clarify how sub-scale means are to be interpreted. Line 252-254 now reads, "*physical-environmental factors were assessed as representing the largest barriers to ocean access while personal factors were the smallest (i.e., sub-scale means in **Figure 4A,** where smaller means represent larger barriers)"*. Line 306-310 now reads, "*material wellbeing factors (e.g., income, employment, food security, etc.) scored lower than subjective and relational wellbeing factors (i.e., sub-scale means in **Figure 6A**, where larger values represent greater perceived contribution to human wellbeing)."*

COMMENT: Improve the connection between the Results and Discussion sections to ensure clarity between key and novel findings and suggested causes for these to help guide the reader through the interpretation of the findings and highlight the contribution made by this work

RESPONSE: Please see previous response to the Editor, Reviewer #1, and Reviewer(s) 3 & 4. The Discussion section has been restructured in order to elaborate upon the recommendations provided in what was previously labeled Table 3 and better justify them as motivated by the study's specific, empirical findings.

COMMENT: Add discussion of limitations of the work and ideas for further work, e.g. other contexts, research to advance overcoming barriers etc.

RESPONSE: See previous response to a comment made by Reviewer #1. The revised text includes a new discussion section (*4.3 Study Limitations and Next Steps for Research).*

COMMENT: A reminder that all error bars should be defined in the corresponding figure legends and should include a description of any error bars.

RESPONSE: In the legends of Figure 5 and 7 we have clarified that, *"error bars indicate 95% confidence intervals"*.

Minor comments:

Line 72: Add supporting reference to the following statement "Geography, gender, class, and race - and the interactions among them - all function to constrain or enhance people's relationship with the ocean".

In the revised version of the text this statement has been supported by two references whose complete citations are copied below.

-Saunders, F., Gilek, M., Ikauniece, A., Tafon, R.V., Gee, K. and Zaucha, J., 2020. Theorizing social sustainability and justice in marine spatial planning: Democracy, diversity, and equity. *Sustainability*, *12*(6), p.2560.

- Crosman, K.M., Allison, E.H., Ota, Y., Cisneros-Montemayor, A.M., Singh, G.G., Swartz, W., Bailey, M., Barclay, K.M., Blume, G., Colléter, M. and Fabinyi, M., 2022. Social equity is key to sustainable ocean governance. NPJ Ocean Sustainability, 1(1), p.4.

Line 72-73: "Though recent research has advanced scientific understanding of the links between marine governance and environmental sustainability" the link between this and the identified gap could be made more explicit.

See response to previous comment made by Reviewer #1. The Introduction of the manuscript has been restructured to more clearly identify and describe the research gap the study is intended to address.

Line 79: Suggest adding what are the major coastal management frameworks that prioritise ecological metrics over social indicators.

In the first paragraph of the revised version of the manuscript, we have added the descriptors "Western" and "Anglo-European" to more clearly delineate the scope of our critique.

Line 99- consider adding, or introduce new inequalities

We respectfully assert a preference for this sentence as it was previously structured.

Line 102: Consider adding definition of key terminologies, in particular, ocean access and wellbeing, compounding, cascading, justice (climate justice, environmental justice, recognitional justice, ocean justice)

Please see previous response to Reviewer #1 and Reviewer #2. The Theoretical Framework section of the revised version of the manuscript has been rewritten to provide the definitions requested and more clearly elucidate the connections between concepts.

Line 146: Figure 1, given that the text refers to Figure 1 in the Theoretical Framework section and not mentioned in the Results section consider moving it

Figure 1 has been moved up in the revised version of the Text.

Lines 158: Figure 2, add scales to the maps

Figure 2 has been reformatted to improve legibility and provide the scale information requested by the reviewer.

Lines 160-161: Figure2, move letter (A) "across California at-large (A) (remove here) and the three focal regions where administration efforts were concentrated: (A) (add here) the Greater Monterey Bay (B) […].

In the reformatted version of **Figure 2**, the insets and complete map have been integrated into the same image rather than being referenced as individual panels.

Line 199: typo NortherN region, missing N

We have fixed the identified typo in the revised version of the manuscript.

229 physical environment repeated twice?

We believe the text was correct as previously written. The first reference to physical-environmental describes it as one of four dimensional sub-scales while the second reference to physical-environmental identifies it as the sub-scale assessed as representing the largest overall barrier to ocean access.

Line 246: typo subS-scales, remove S

We have fixed the identified typo in the revised version of the manuscript.

Line 233: Table 1, it would benefit from a better explanation of the composite scale and how multiple dimensions were created.

We would direct the Reviewer to what are now Lines 642-649 of the methods text which clarify, "*Long-format, likert-scale matrix questions (each containing 9 prompts with which respondents were asked to indicate their level of agreement using the (5) categories Strongly*

Disagree, Disagree, Neither agree nor disagree, Agree, or Strongly Agree) were used to assess barriers to ocean access and the contribution of the ocean to human wellbeing. For each parameter (i.e., Barriers and Ocean Wellbeing) a composite index was derived by finding the mean response value per respondent following the transformation of the likert-scale into numerical values (i.e., Strongly Agree = 5, Agree = 4, etc.). Dimensional subscales for each parameter (see ***Table 1****) were subsequently constructed following the same procedure*."

Line 247: Tukey's Honestly Significant Difference (HSD) – could be spelled out since is the first time being mentioned

We have modified the text, now found on Line 255 of the revised version of the manuscript, so that it now reads, "t*hough a post-hoc Tukey Honestly Significant Difference (HSD) test….*"

Line 313: Figure 5, where can we find the regression tables?

As referenced in Line 696-698 of the Methods section of the revised version of the manuscript, the final regression tables for all models and sub-models can now be accessed online via the github repository linked in the Data & Code availability section.

Line 361: in the statement "Significant barriers reported by Black and Asian American respondents, [..] are the result of structural processes embedded in racialized histories" – while one may agree please ensure careful phrasing to ensure distinction between suggestion for causes and clear results from the study.

Please see response to a previous comment made by this reviewer.

Line 352: While one may infer the meaning of structural barriers and you provide examples a definition could guide the reader.

On Lines 121-123 of Theoretical Framework section of the revised version of the manuscript, what is meant the term "structural" is explicitly defined in the following text: "*Thus we consider barriers to ocean access not as random inconveniences, but as structural processes (i.e., persistent institutional, economic and social arrangements that shape individual agency) responsible for mediating this ability and determining the distribution of ocean benefits across the California coast…*"

Line 398: "previous literature suggests that […] subsistence goals" please provide references of previous studies.

In the revised version of the text this statement has been supported by two references whose complete citations are copied below.

Morales-Nin, B., Arlinghaus, R. and Alós, J., 2021. Contrasting the motivations and wildlife-related value orientations of recreational fishers with participants of other outdoor and indoor recreational activities. *Frontiers in Marine Science*, *8,* p.714733.

Frawley, T.H., Krauss, M., Kruesopon, P.S., Meykadeh, R., Triviño, T., Gee, E., Selgrath, J., Crowder, L. and Seary, R., 2025. From Shorelines to Social Media: Mixed-Methods Insights into Urban Fishing Practices, Policy Gaps and Culture in the Digital Age. *EcoEvoRxiv*.

Lines 718-719: Supplemental Table 1. It would be helpful to have the key references in relation to each definition and subcategory rather than "barriers to ocean" as a whole

Given the substantial number of additional references already added to the manuscript during the revision, in this instance we feel it is most appropriate to direct readers to the authors who originally synthesized the literature in developing these frameworks rather than attempting to reproduce their efforts here ourselves.

Lines 718-719: Supplemental Table 1, unless in the authors to guidelines, move table captions to the top of the table

The table caption has been moved to the top of the table.

Lines 722-723: Supplemental Table 2, unless in the authors to guidelines, move table caption to the top of the table

The table caption has been moved to the top of the table.

Lines 731-732: order of bar plots is not mirroring what is in the caption. A) racial-ethic categories, B) annual household income, C) frequency of use of ocean and coastal areas as opposed to "A) annual household income, B) frequency of use of ocean and coastal areas, and C) racial-ethnic categories."

We thank the Reviewer(s) for their careful eye in identifying mismatch between the order in which the panels were presented and accompanying legend text. We have corrected this error in the revised version of the manuscript.

Dear Reviewers,

We are grateful for your thoughtful engagement with the revised manuscript and for the minor, constructive comments offered in this second round of review. We are pleased that all Reviewers found their initial concerns addressed effectively and comprehensively in the previous round of revisions, and have carefully considered and responded to the remaining minor points raised by Reviewers #2 and #5 in additional edits to the manuscript. For more details, please see the specific replies to individual comments presented below.

REVIEWER COMMENTS

Reviewer #2 (Remarks to the Author):

Overall, the authors have done a fantastic job addressing all of the reviewer comments including those from me. I recommend it be accepted subject to a few minor revision below.

A couple of minor points that I think should still be addressed:

COMMENT: 1) Ocean equity - While I do see the topic of access and human wellbeing related to ocean equity (and many of the other theories mentioned in the paper), I think these topics can be briefly introduced and the link made in the introductory text to situate the topic. But, it seems like a stretch to include "distributional ocean equity" in the theoretical framework for the paper (i.e., figure 1) as this topic is not really in the survey. The survey is about ocean access and human wellbeing. I would suggest to remove "distributional ocean equity" from the figure, it is a distraction from the main topics of the paper (i.e., access and wellbeing) which stand on their own.

RESPONSE: We appreciate the Reviewer's continued engagement with the theoretical foundation of the manuscript. We acknowledge that in trying to address Reviewer #1's request to formalize the manuscript's conceptualization of "ocean equity", we may have gone too far in attempting to use our indicators as a means of operationalizing the measurement of "distributional ocean equity". Following the Reviewers suggestion, in this revision we have edited the text and figures so that equity is understood as a normative motivation for the study and a lens through which to interpret results rather than a directly quantified construct. Accordingly, reference to "Distributional Ocean Equity" has been removed from **Figure 1** and the following edits have been made to the text to support this re-framing and ensure consistent, internal logic:

1. Language describing the objective of and motivation for the study in the Introduction (i.e., Lines 88-89 of the revised text has been changed from, "*we investigate the complex, multidimensional relationships between ocean access and benefits across California's central coast, using perceived contribution to human wellbeing as a proxy for distributional ocean equity*" to "*we investigate the complex, multidimensional*

relationships between ocean access and benefits across California's central coast, and identify variation in relevant patterns and processes across different demographic groups."

2. Modification of the text which concludes the first paragraph of the Theoretical Framework section (i.e., Lines 114-118).

Original Text: "*This study operationalizes and interrogates ocean equity through a two-step analytical process: first, by investigating access barriers as the structural mechanisms mediating ocean engagement; and second by measuring perceived contribution to human wellbeing as the primary indicator of resulting distributive outcomes (**Figure 1**). While equity is a complex and multidimensional process, considered both as a characteristic of society in which governance occurs and an outcome of governance*[3,49]*, here we primarily concern ourselves with distributional ocean equity*[15,50] *rather than procedural or recognitional components.*"

Modified Text: "*Building upon this evolving scholarship, our study examines two distinct but interrelated dimensions of the human-ocean relationship which we argue are fundamental for understanding how ocean benefits are distributed across diverse coastal populations: the structural barriers that mediate access to ocean and coastal spaces and the ocean's contributions to human wellbeing (**Figure 1**).*"

3. Results subheading 3.4, previously labeled "*Processes and Pathways Shaping Distributive Equity*" has been changed to "*Processes and Pathways Shaping the Distribution of Ocean Benefits*".

4. The opening sentence of Section 4.4 (i.e., Line 511-512) of the revised version of the manuscript has been modified to remove reference to "*distributional ocean equity*" as a theoretical construct and replace it with "*the equitable distribution of ocean benefits*" as a normative goal.

COMMENT: 2) Survey items - I appreciated that the authors have put the general survey items for each of ocean access and human wellbeing in the Supplemenatary materials. However, it would still be good to include a short introduction to the general survey items in the body of the text - alongside an introduction/references to the theory that they are meant to support. (E.g., Drawing on human well-being theory (ref, ref, ref), we developed survey indictors for .... the following aspects of human wellbeing ... which we asked participants to score as follows... Similarly, for access, we created indicators related to the X, Y, Z, mechanisms that mediate access...etc.) Or, you might use an abbreviated table to bring readers up to speed. Without introducing this, it is hard for the reader to follow the methods and theoretical framework that guides the analysis.

RESPONSE: We take the Reviewer's point that a short introduction to the general survey items in the body of the text would be a useful addition to the Supplementary Materials added in the previous round of revision in helping the readers understand and follow the mechanics of the analysis. The following text insertions have been made to the 2nd and 3rd paragraphs of the Theoretical Framework section.

On Lines 137-142 of the revised text, we introduce the barriers indicators: "*Informed by previous literature (Ribot & Peluso, 2003; Byrne & Wolch, 2009; Wang et al., 2015; Seto et al., 2024), we measure barriers to ocean access through nine Likert-scale survey prompts operationalized across four dimensions (see **Supplemental Table 1**):* personal *(e.g., transportation, proximity, affordability),* social *(e.g., welcomeness, belonging, social marginalization),* physical-environmental *(e.g., amenities, pollution, exposure to hazardous conditions), and* knowledge *(e.g., understanding of rules and regulations, possession of necessary skills, familiarity with access points).*

On Lines 151-157 of the revised text, we introduce the wellbeing indicators: *Drawing on theories of human wellbeing (White et al., 2010; Weeratunge et al., 2014; Allison et al., 2023), we measure the ocean's perceived contribution to wellbeing through nine Likert-scale survey prompts operationalized across three dimensions (see **Supplemental Table 2**):* material *(e.g., food, income, physical health),* relational *(e.g., community, cultural identity, intra-household relations), and* subjective *(e.g., mental health, spirituality, aesthetic experience). By coupling this multidimensional view of human wellbeing with a structural analysis of ocean access, we aim to map how the ocean's benefits vary across California's diverse coastal populations.*

Reviewer #3 (Remarks to the Author):

COMMENT: I appreciate the authors' thorough consideration and responses to the reviewer comments. For my part, the points I raised in my initial review have been addressed, and I have no remaining concerns about the manuscript.

RESPONSE: We are grateful to Reviewer #3 for their thoughtful engagement across both rounds of review and are pleased to hear that the revisions fully address their concerns.

Reviewer #5 (Remarks to the Author):

COMMENT: Thank you to the authors for their careful and thorough consideration of our comments on the manuscript, I can see from the response document and the manuscript that these have been very well taken into consideration.

I particularly appreciate the effort taken with the introduction and theoretical framework to strengthen and contextualise the contribution, as well as the clarification of definitions that have been added.

I thank the authors for the clarifications on the presentation of the gender variables in the figures and appreciate the effort taken to provide more information for those readers wishing to consider the analysis through the lens of gender.

I also feel that the reformatting of table 1 as a figure has improved the presentation of the results.

There has been some softening of the language concerning causality between results and likely causes and the additional discussion of these is now excellent. The introduction of the Policy implictions of the work and the Study Limitations and Next Steps for Research section is also welcomed. I was very pleased to see the introduction of a paragraph on the motivation for the study, although I think this would be better placed in the introduction, so that it can provide context for the reviewer. Overall, the manuscript has been improved by the responses to the reviewer comments and I only have a few remaining minor things to consider before publication.

RESPONSE: We thank Reviewer #5 for their generous and detailed assessment of the revision. The three remaining minor items have been addressed as described below.

COMMENT: First, the mention of Jim Crow South may not make sense to those not familiar with US history, as Nature Communications has an international audience this should be re-written to explain e.g. systemic racial segregation enforced in the south of the country.

RESPONSE: On Lines 53-54 of the Revised manuscript, a parenthetical has been added to this sentence referencing the Jim Crow South to explain it as, “a system of legally codified racial segregation and disenfranchisement in the southern United States”

COMMENT: Second, could a reference for California Environmental Protection Agency’s CalEnviroScreen index be added?

RESPONSE: The complete reference for the CalEnviroScreen index has been added to the manuscript (as reference #40), and corresponding citations have been added to the revised manuscript on Lines 91 and 635.

COMMENT: Finally, consider moving the motivation statement to the introduction.

RESPONSE: We thank the Reviewer for this suggestion and agree on reflection that the motivation statement functions more effectively in the Introduction, where it can orient readers to the study's origins before they encounter the research questions. In the revised version of the manuscript this text has been moved from the conclusion of the methods section to the beginning of the final paragraph of the Introduction (i.e., Lines 77-84).
